# The exposure-lag-response association between solar radiation components and meibomian gland dysfunction in Shanghai, China

**DOI:** 10.3389/fpubh.2026.1797475

**Published:** 2026-03-06

**Authors:** Han Zhao, Zile Yu, Wushuang Wang, Yun Yang, Tong Lin

**Affiliations:** 1 Department of Ophthalmology, Second Xiangya Hospital of Central South University, Changsha, Hunan, China; 2 Hunan Clinical Research Center of Ophthalmic Disease, Changsha, Hunan, China; 3 Eye Center of Xiangya Hospital, Central South University, Changsha, Hunan, China; 4 Department of Ophthalmology, Eye & ENT Hospital, Fudan University, Shanghai, China; 5 NHC Key Laboratory of Myopia and Related Eye Diseases, Key Laboratory of Myopia and Related Eye Diseases, Chinese Academy of Medical Sciences, Shanghai, China; 6 Shanghai Key Laboratory of Visual Impairment and Restoration, Shanghai, China

**Keywords:** air pollutions, DLNM model, meibomian gland dysfunction, Shanghai, solar radiation, time series

## Abstract

**Objective:**

Meibomian gland dysfunction (MGD) is a prevalent cause of evaporative dry eye disease with significant public health implications. This study investigated the potential impact of solar radiation (SR) on the incidence of MGD in Shanghai, China.

**Methods:**

Daily information was collected in Shanghai, China, from January 1, 2017, to December 31, 2023, including air pollution, outpatient visits for MGD, meteorological data, and SR data. A distributed lag nonlinear model was used to explore the relationships between SR and MGD, taking into account potential nonlinear exposure-response relationships and lag effects. Additionally, the study used weighted quantile sum and quantile-based g-computation models to examine whether SR components independently affected MGD.

**Results:**

The present study identified 64,038 records of outpatient visits for MGD. Exposure to global horizontal irradiance, diffuse horizontal irradiance, and direct normal irradiance was associated with an increased risk of MGD outpatient visits at the lags of 0–3 days, 7–14 days, and 0–15 days, respectively. Furthermore, the study identified subgroup-specific effects of SR, which differed by age, gender, and season.

**Conclusion:**

These results underscore the importance of considering environmental factors in understanding the prevalence of MGD. The study was conducted in Shanghai, China, and provides valuable insights into the potential risk factors for MGD in this region.

## 1 Introduction

Meibomian gland dysfunction (MGD) is an important cause of evaporative dry eye disease (DED), which has become a global public health problem (1). The reported incidence and prevalence of MGD varies widely by ethnicity. In the past decade, the incidence of MGD among Chinese adults has exceeded 69% (2), whereas it ranges from 3.5 to 19.9% among Caucasians (1, 3). The tear film defends healthy eyes from pathogens, environmental damage, and other kinds of stress by acting as an external and immunological barrier (4, 5). Meibum produced by the meibomian glands constitutes the outer layer of the tear film and stabilizes the film, reduces surface tension, and prevents evaporation of aqueous tears (6). MGD may be characterized by terminal duct blockage, meibomian gland dropout, orifice plugging, and abnormal meibum secretion, which can lead to hyperosmolarity, disruptions in the tear film, clinically noticeable inflammation, and symptoms of discomfort (7).

Studies report that DED is related to a greater mental burden and a poorer health-related quality of life (8, 9). In addition to causing a significant mental burden, MGD has a great economic cost. According to the Tear Film and Ocular Surface Society’s (TFOS) Dry Eye Workshop (DEWS) II report, MGD accounts for 60% of DED cases (10). The medical care system in the United States was expected to spend $3.8 billion in direct financial costs annually as a result of DED treatment. From the perspective of society, the average cost of treating DED has been calculated at $11,302 for each patient (11), suggest the urgency of investigating the potential causes of MGD episodes.

Scientific research has revealed a connection between solar radiation (SR) exposure and human diseases. For example, a multicenter study in the United States found that daily SR exposure could decrease the risk of hypertensive disorders of pregnancy (12), and a previous epidemiological study reports that increased SR exposure is correlated with solar dermatitis (13). Some studies indicate that SR has a beneficial impact on the onset and course of schizophrenia (14). Booij et al. (15) report that the dopamine transporter has an obvious seasonal variation among Parkinson’s disease patients, with a greater expression in spring/summer. Other studies reveal SR exposure’s association with ocular diseases. For instance, greater sunlight duration has been linked to a higher prevalence of cataracts and macular degeneration (16, 17), and a systematic review reports that long-term exposure to SR radiation raises the risk of pterygium (18). Although the exact mechanism underlying these relationships is unclear, SR has the potential to interact with biological tissues through photochemical and thermal effects, mainly through light in the ultraviolet (UV) and infrared wavelengths, respectively (19).

The severity and duration of SR are expected to increase due to global warming (20, 21). An epidemiological study in Norway found seasonal fluctuations in various DED assessment factors. For instance, patients have a smaller tear meniscus height during winter than in the other seasons (22). In addition, a study drawing from the National Veterans Administration database reports that the incidence of DED was lower during summer than in winter or spring (23). However, the impact of SR on MGD has not been measured in many studies to date, and it remains uncertain whether disease burden is directly related to SR.

In addition to SR, air pollution is a broadly acknowledged significant risk factor for MGD (24–26). For example, an increase in the concentration of O_3_ and SO_2_ has been linked to an increase in the level of tear pro-inflammatory cytokine and serious signs of MGD (24). Moreover, an elevated value of the air quality index (AQI) is a risk factor for the occurrence of MGD (26). These data lead us to hypothesize that air pollution and SR may exert a combined influence on the incidence of MGD.

When evaluating exposure-response relationships, it is important to consider the time pattern of the effects, which frequently lag in cases of environmental exposure. To accurately characterize potential nonlinear exposure-response relationships along with lag effects (often known as exposure-lag-response relationships), a modeling framework was established called the distributed lag nonlinear model (DLNM) (27). This study used a distributed lag model to explore the relationships between SR and MGD and evaluated the differing subgroup-specific effects of SR by age, gender, and season. Additionally, we used two statistical models—the weighted quantile sum (WQS) regression model and the quantile-based g-computation (qgcomp) model—to examine whether any of the three SR components independently affected MGD in the participants in our research.

## 2 Materials and methods

### 2.1 Study area

**Figure 1:**
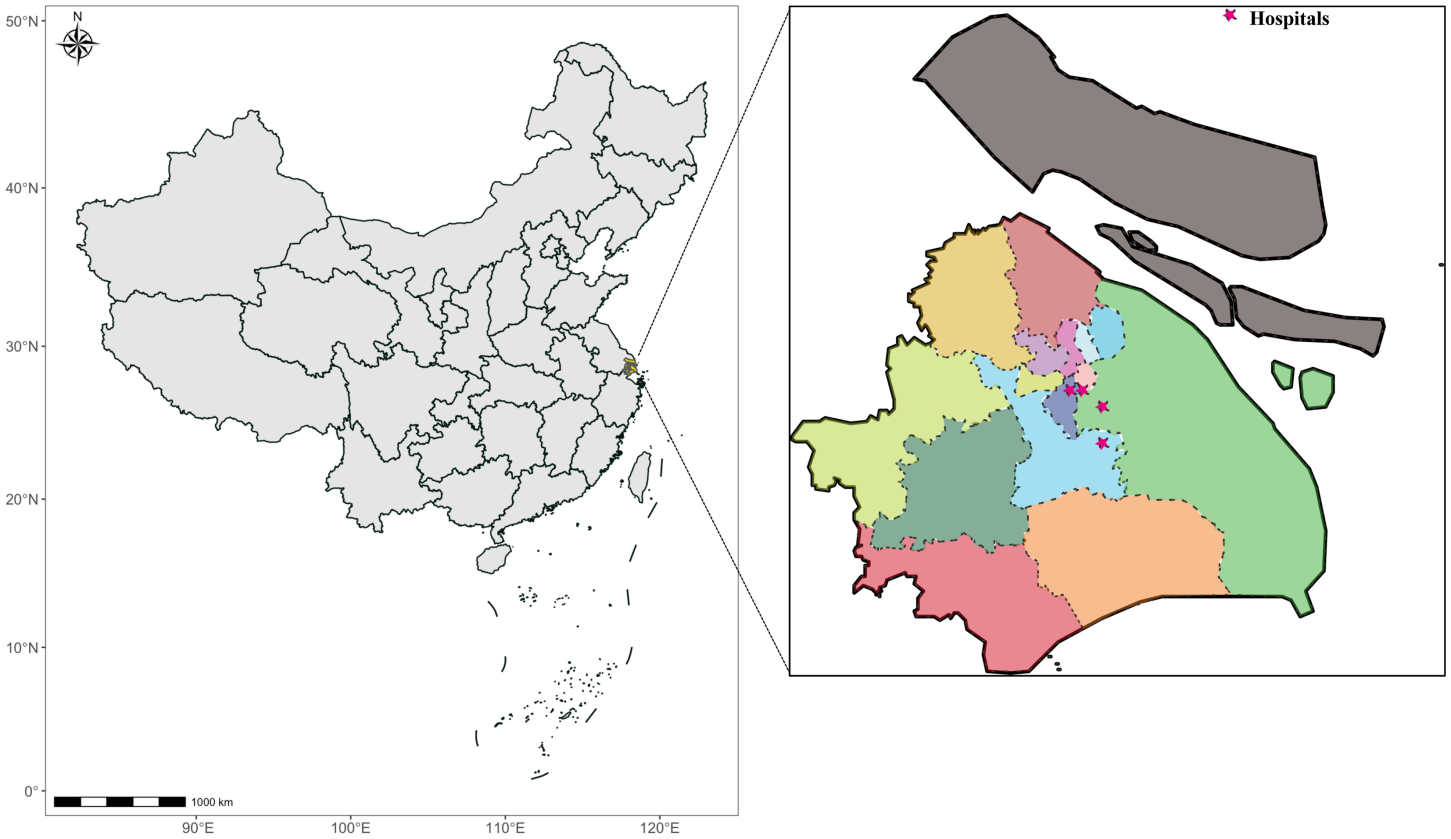
Geographic location map of Shanghai city and the hospital.

The study was conducted in Shanghai, China, located on the alluvial plain of the Yangtze River delta by the Pacific Ocean between longitudes 120°52′ and 122°12′ and latitudes 30°40′ and 31°53′ on China’s east coast at the edge of the Asian continent (Figure 1). With an average elevation of 2.19 meters above sea level, Shanghai has a subtropical monsoon climate and a river network that consists mainly of the Huangpu River (the main waterway through the city) and its tributaries, the Suzhou, Yangtze, and Dianpu rivers. Shanghai is the largest economic center in China and an important international financial center, with an administrative area of 6340.5 square kilometers (km^2^) and a resident population of 24,772,000.

### 2.2 Data collection

To inform the retrospective study, the information technology (IT) department of the Eye, Ear, Nose, and Throat Hospital of Fudan University, the largest ophthalmic hospital in Shanghai, provided MGD data from outpatient visits between January 1, 2017, and December 31, 2023. The *International Classification of Diseases, 10th Revision* (ICD-10) was used to determine MGD according to definition code H02.500×008. Patients who were revisiting were excluded from this study. For outcome ascertainment, we used the primary diagnosis field from outpatient records. Each visit-date was counted as one observation, and only visits with MGD as the primary diagnosis were included. To avoid potential bias from repeated measures, we defined ‘revisiting’ patients as those with any prior MGD-related outpatient visit recorded during the study period (2017–2023). Only the first-ever MGD visit for each patient was included in the analysis; all subsequent visits were excluded, ensuring independence of observations. The outpatients’ details were anonymous and archived, including patient number, date of birth, date of visit, gender, and residential street address, and the hospital’s ethics committee approved the research procedures. No participants were contacted, and no medical record data were made available to the general public.

The SR data were obtained from the European Centre for Medium-Range Weather Forecasts (ECMWF), including data on diffuse horizontal irradiance (DHI), direct normal irradiance (DNI), and global horizontal irradiance (GHI). DHI is defined as the radiation that reaches the Earth’s surface in diffuse form as a result of sunlight being scattered by clouds, gas molecules, dust, and the like as it passes through the atmosphere. DNI is defined as the amount of SR received per unit area in the direction perpendicular to the ground. The GHI value refers to the total SR received on the horizontal surface of the ground. The DHI and DNI are components of the GHI.

GHI=DNI×cos(solar zenith angle)+DHI

Other meteorological factors were acquired from the Shanghai Environmental Protection Agency, include daily average temperature (°C), relative humidity (%), wind speed (m/s), precipitation (mm/24-h), and air pressure (hPa). We also obtained data from the Shanghai Environmental Protection Agency on six standard ambient air pollutants, including particulate matter of less than 2.5 μm or 10 μm (PM_2.5_ or PM_10_), nitrogen dioxide (NO_2_), carbon monoxide (CO), sulfur dioxide (SO_2_), and 8-h mean ozone (O_3_). The daily data of ambient air pollutants and meteorological factors were averaged for analysis. The initial data were used for data cleaning and filtering to assure accuracy and reasonableness before further study.

### 2.3 Statistical analysis

The characteristics of daily outpatient visits for MGD, the level of SR, the levels of meteorological factors, and the concentrations of ambient air pollutants in Shanghai City from 2017 to 2023 were summarized by means, standard deviations, and percentiles. Plots were created showing the daily meteorological and ambient air pollutant parameters, the SR, and the MGD over the same period. Correlations were identified between MGD, SR, ambient air pollutants, and meteorological data, and correlation statistical analysis techniques were used to explore the relationship between dominating components.

We used a quasi-Poisson generalized linear model (GLM) to determine the lag days of SR (DHI, DNI, and GHI) and assess the relationship between SR and outpatient visits for MGD, measured as cumulative relative risk (RR). Due to its ability to determine both delayed impacts and nonlinear exposure-response correlations simultaneously, the distributed lag nonlinear model (DLNM) has commonly been used to examine relationships between environmental variables and health outcomes (28, 29). According to Gasparrini et al., DLNMs effectively eliminate collinearity across lag exposures and reduce the likelihood of overestimating the immediate effects of exposure, enabling a more precise assessment of the real single effects (27). Furthermore, by adding the single impacts across a series of lag days, cumulative effects can be calculated. The final DLNM model was expressed as follows:

$$\begin{array}{l} \log[E_{Y}]=\alpha +\text{cbSolar radiatio}n_{t,\;\mathrm{lag}}+\mathrm{ns}(\text{Mean temperatur}e_{t},\mathrm{df}=3)\\ +\mathrm{ns}(\text{Relative humidit}y_{t},\mathrm{df}=3)+\mathrm{ns}(\text{Precipitatio}n_{t},\mathrm{df}=3)\\ +\mathrm{ns}(\mathrm{Air}\;\text{pressur}e_{t},\mathrm{df}=3)+\mathrm{ns}(\text{Wind spee}d_{t},\mathrm{df}=3)\\ +\mathrm{ns}(PM_{2.5,\;t},\mathrm{df}=3)+\mathrm{ns}(PM_{10,\;t},\mathrm{df}=3)+\mathrm{ns}(SO_{2,\;t},\mathrm{df}=3)\\ +\mathrm{ns}(NO_{2,\;t},\mathrm{df}=3)+\mathrm{ns}(CO_{t},\mathrm{df}=3)+\mathrm{ns}(O_{3,\;t},\mathrm{df}=3)\\ +\mathrm{ns}(\mathrm{Tim}e_{t},\mathrm{df}=7\times 6)+\mathrm{\beta DO}W_{t}+\text{γHolida}y_{t} \end{array}$$

where *E_Y_* is the daily count of outpatient visits for MGD at calendar day *t* (1, 2, 3, …, 2556), *α* represents the intercept, *cb* is the cross-basis matrix, and *lag* represents lag days. To maintain coherence with earlier research, we used a natural cubic spline (ns) with 7 degrees of freedom (df) annually for time so as to optimize for long-term and seasonality patterns. Based on earlier research, a lag structure with a maximum lag of 21 days was employed to capture the single and cumulative lag effects between exposure to SR and MGD (14, 30). Additionally, we included dummy variables in the model for day of the week (DOW) and holidays (Holiday); *β* and *γ* represent the coefficients of DOW and Holiday. We used Akaike’s information criteria for quasi-Poisson regression to calculate the df for exposure to SR and the lag days.

Three variables (gender, age, and season) were used to perform stratification analyses. There were two gender groups: male and female. Age was coded as 0–18 years, 19–60 years, and over 60 years. Season was divided into two categories: the warm season (April to September) and the cold season (October to March). The statistical significance of all subgroup characteristics was evaluated at a 95% confidence level using the following formula:

$$(\hat{Q_{1}}-\hat{Q_{2})}\pm 1.96\sqrt{{\left(S\hat{E_{1}}\right)}^{2}-{\left(S\hat{E_{2}}\right)}^{2}}$$

where $\hat{Q_{1}}$ and $\hat{Q_{2}}$ are the effect estimates of each group and $S\hat{E_{1}}$ and $S\hat{E_{2}}$ are the standard errors of each group.

To evaluate the robustness of our findings, multiple sensitivity analyses were conducted by changing the df for time (6–8), ambient air pollutants (2–4), and meteorological factors (2–4). We also calculated the results by adjusting the maximum lag day to 23 days. To assess whether collinearity among air pollutants influenced our solar radiation effect estimates, we performed leave-one-out sensitivity analyses. The main DLNM model was refitted multiple times, each time excluding one air pollutant.

To investigate the relationships among concurrent exposure to DHI, DNI, and GHI, we used the WQS regression model, a recently developed machine learning methodology for large-scale datasets. In the environmental epidemiology field, the WQS regression model is widely used to investigate the effects on certain outcomes of numerous exposures to environmental substances (31, 32). In brief, the WQS regression model initially generates a weighted linear index that characterizes the total burden of the components of interest by grouping all the analytes into quantiles. The relative intensity of the weights that the model allocates to each variable can subsequently be used to evaluate the contribution of each environmental variable to the overall index’s impact, enabling the identification of significant variables in the mixture. The WQS index is determined by the empirical weight of each factor, which is a number between 0 and 1. All weights are restricted to adding up to 1. Based on the GHI, DHI, and DNI quartiles, we created the WQS index of SR exposure for this research. The WQS was calculated as follows:

$$\mathrm{WQS}=\sum_{i=1}^{c}{\bar{w}}_{i}q_{i}$$

where *c* is the number of exposed variables, *w* is the weights of the exposure variables, and *q* is the quantile score.

Using the qgcomp model, we also evaluated how all three combined SRs affected MGD outpatient visit. In estimating the impacts of a combination of all exposures, qgcomp produces reliable conclusions about individual contributions in both positive and negative directions. This method combines the flexibility of g measurement with the comfort and simplicity of using weighted quantile regression. We set *q* = 4, meaning each solar radiation variable was transformed into quartiles based on its distribution across the study period. This quartile discretization allows the model to capture potential non-linear effects of the exposure mixture while maintaining interpretability. Because our outcome was daily counts of outpatient visits, we used a quasi-Poisson family with a log link function to account for overdispersion commonly observed in count data. The overall mixture effect was estimated as the sum of the positive (or negative) coefficients when all exposures were increased by one quartile simultaneously (33).

All our statistical analyses were performed using R software (ver. 4.3.2 for Windows) with the R packages dlnm, nlme, gWQS, qgcomp, and ggplot2. A *p*-value of <0.05 was defined as statistically significant.

## 3 Results

### 3.1 Descriptive statistics

Tables 1, 2 present a summary of the descriptive statistics for Shanghai’s daily outpatient visits for MGD, meteorological variables, and air pollutants from 2017 to 2023. During the research period, we evaluated the records of 64,038 cases of MGD with an average age of 49.70 years (range: 1–91). Outpatient visits for MGD numbered 25.05 cases daily on average, with middle-aged patients (18–60 years old) and females constituting a greater portion of daily admissions than the other demographic groups. The mean daily concentrations of PM_2.5_ (μg/m^3^), PM_10_ (μg/m^3^), SO_2_ (μg/m^3^), NO_2_ (μg/m^3^), and CO (μg/m^3^) were 33.92, 47.45, 12.86, 37.51, and 0.86, respectively. The 8-h mean O_3_ (μg/m^3^) was 84.66. The means of daily meteorological variables, including temperature (°C), relative humidity (%), wind speed (m/s), air pressure (hPa), and precipitation (mm/24 h) were 18.59, 76.54, 3.69, 1016.08, and 3.18, respectively. The means of daily GHI (W/m^2^), DNI (W/m^2^), and DHI (W/m^2^) were 187.64, 142.10, and 97.90, respectively.

**Table 1:** Description summary of daily outpatient visits for meibomian gland dysfunction in Shanghai, 2017–2023.

Variables	Sum	Mean	SD	Minimum	*P*_25_	Median	*P*_75_	Maximum
Daily outpatient visits	64,038	25.05	34.77	0	0	8	36	166
Gender								
Female	43398	16.98	24.13	0	0	5	23	117
Male	20640	8.08	11.03	0	0	3	12	58
Age (year)								
<18 y	837	0.33	0.70	0	0	0	0	5
18–60 y	43,364	16.97	23.12	0	0	6	25	113
>60 y	19,835	7.76	11.82	0	0	2	11	63
Season								
Warm season (April to September)	31,485	24.58	33.80	0	0	10	35	166
Cold season (October to March)	32,553	25.53	35.74	0	0	7	36	155

SD, standard deviation; *P*_25_, 25th percentile; *P*_75_, 75th percentile.

**Table 2:** Description summary of daily air pollutant concentration and meteorological factors in Shanghai, 2017–2023.

Variables	Mean	SD	Minimum	*P*_25_	Median	*P*_75_	Maximum
Air pollutant concentration (24-h average)							
PM_2.5_ (μg/m^3^)	33.92	24.05	0	17	27	43	191
PM_10_ (μg/m^3^)	47.45	27.14	0	29	41	61	308
SO_2_ (μg/m^3^)	12.86	18.96	1	5	7	10	119
NO_2_ (μg/m^3^)	37.51	21.12	0.5	23	33.5	48	119
CO (μg/m^3^)	0.86	1.10	0	0.5175	0.63	0.8	11
Air pollutant concentration (8-h average)							
O_3_ (μg/m^3^)	84.66	40.66	0	57	79	106	295
Meteorological factors							
Mean temperature (°C)	18.59	8.74	−4.5	11	19	26	35.5
Relative humidity (%)	76.54	10.36	39	70	78	84	98
Wind speed (m/s)	3.69	1.42	1	3	3	4	14
Air pressure (hPa)	1016.08	9.12	987	1008	1017	1023	1042
Precipitation (mm/24h)	3.18	8.38	0	0	0.115	2.1325	117.86
Solar radiation							
GHI (W/m^2^)	187.64	109.12	10.09	107.31	171.71	254.14	664.67
DNI (W/m^2^)	142.10	128.73	0.27	23.63	123.59	224.99	690.01
DHI (W/m^2^)	97.90	49.34	9.97	62.86	90.52	122.98	296.70

SD, standard deviation; *P*_25_, 25th percentile; *P*_75_, 75th percentile; PM_2.5_ and PM_10_, particulate matter less than 2.5 and 10 μm; O_3_, ozone; NO_2_, nitrogen dioxide; CO, carbon monoxide; SO_2_, sulfur dioxide; GHI, global horizontal irradiance; DHI, diffuse horizontal irradiance; DNI, direct normal irradiance.

**Figure 2:**
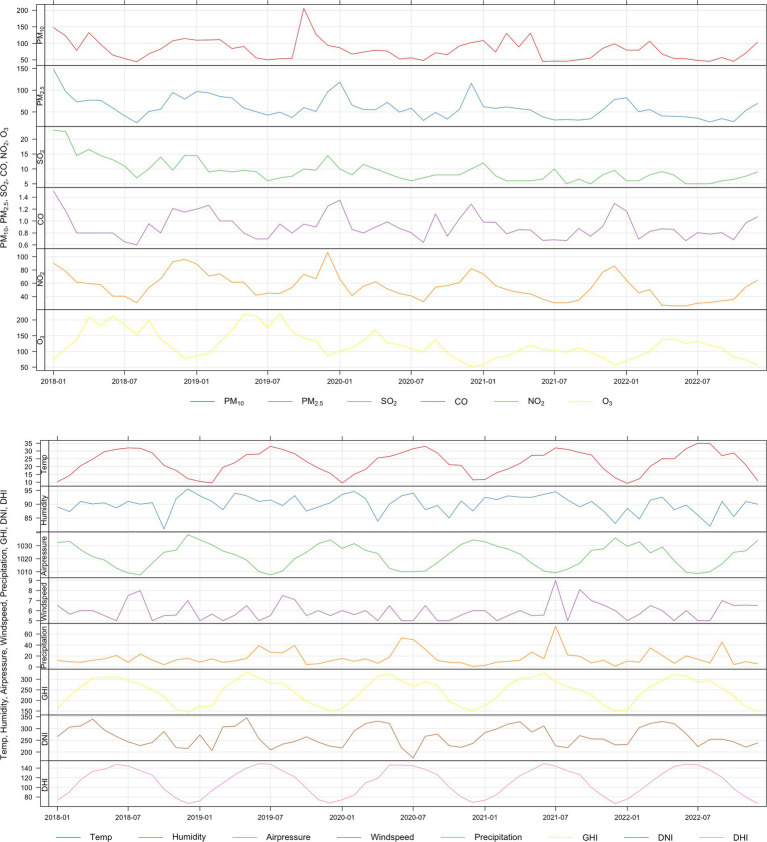
Time series plot showing the temporal fluctuation of daily outpatient visits for meibomian gland dysfunction, air pollutants, and meteorological variables in Shanghai, China, from January 1, 2017, to December 31, 2023.

Supplementary Figure 1 displays boxplots of daily DHI, DNI, and GHI. There was a notable seasonal trend in the number of days with sunshine and the amount of SR on earth; these data peaked and decreased in the summer and winter, respectively. Figure 2 shows the concentrations of air pollutants, the meteorological variables, and the temporal changes of MGD cases over the duration of the research. MGD consistently peaked in the cold season, particularly in November and December. Additionally, there was a modest increase in the prevalence of MGD and air pollutants at the same time, particularly for NO_2_, PM_2.5_, and PM_10_.

### 3.2 Correlation analysis

Supplementary Figure 2 and Table 3 show the Spearman’s rank correlation coefficients between meteorological variables and air pollutants. Importantly, there were significant correlations between the meteorological variables, ranging from −0.85 to 0.74. By contrast, there were weak or no associations between any of the three air pollutants and the meteorological variables. Notably, GHI and DNI were negatively correlated with precipitation (GHI: *r* = −0.57; DNI: *r* = −0.71) and humidity (GHI: *r =* −0.41; DNI: *r =* −0.65) but positively correlated with PM_10_ (GHI: *r =* 0.27; DNI: *r =* 0.41) and SO_2_ (GHI: *r =* 0.28; DNI: *r =* 0.32). DHI showed a positive correlation with mean temperature (*r =* 0.64) and a negative correlation with air pressure (*r =* −0.59).

**Table 3:** The Spearman’s correlation coefficients of daily meteorological factors and air pollutants in Shanghai, 2017–2023.

Variables	PM_2.5_	PM_10_	SO_2_	CO	NO_2_	O_3_	Temp	Humidity	Air pressure	Precipitation	Wind speed	GHI	DNI	DHI
PM_2.5_	1													
PM_10_	0.74	1												
SO_2_	0.5	0.55	1											
CO	0.62	0.51	0.45	1										
NO_2_	0.56	0.48	0.39	0.39	1									
O_3_	0.1	0.17	0.18	−0.02	−0.14	1								
Temperature	−0.26	−0.24	−0.2	−0.28	−0.3	0.31	1							
Humidity	−0.12	−0.4	−0.3	−0.08	−0.06	−0.21	0.27	1						
Air pressure	0.15	0.23	0.19	0.19	0.24	−0.24	−0.85	−0.47	1					
Precipitation	−0.26	−0.45	−0.47	−0.16	−0.17	−0.13	0.17	0.64	−0.32	1				
Wind speed	−0.29	−0.2	−0.09	−0.25	−0.4	−0.06	−0.07	−0.03	0.03	0.1	1			
GHI	0.12	0.27	0.28	−0.02	−0.12	0.44	0.43	−0.41	−0.3	−0.57	−0.1	1		
DNI	0.21	0.41	0.32	0.08	0.05	0.33	0.08	−0.65	0.07	−0.71	−0.11	0.85	1	
DHI	−0.04	−0.03	0.09	−0.09	−0.26	0.29	0.64	0.07	−0.59	−0.13	−0.08	0.65	0.24	1

Statistically significant (*p* < 0.05) were labeled in bold font. PM2.5 and PM10, particulate matter less than 2.5 and 10 μm; O_3_, ozone; NO_2_, nitrogen dioxide; CO, carbon monoxide; SO_2_, sulfur dioxide; GHI, global horizontal irradiance; DHI, diffuse horizontal irradiance; DNI, direct normal irradiance.

### 3.3 Effects of daily GHI, DHI, and DNI on MGD

**Figure 3:**
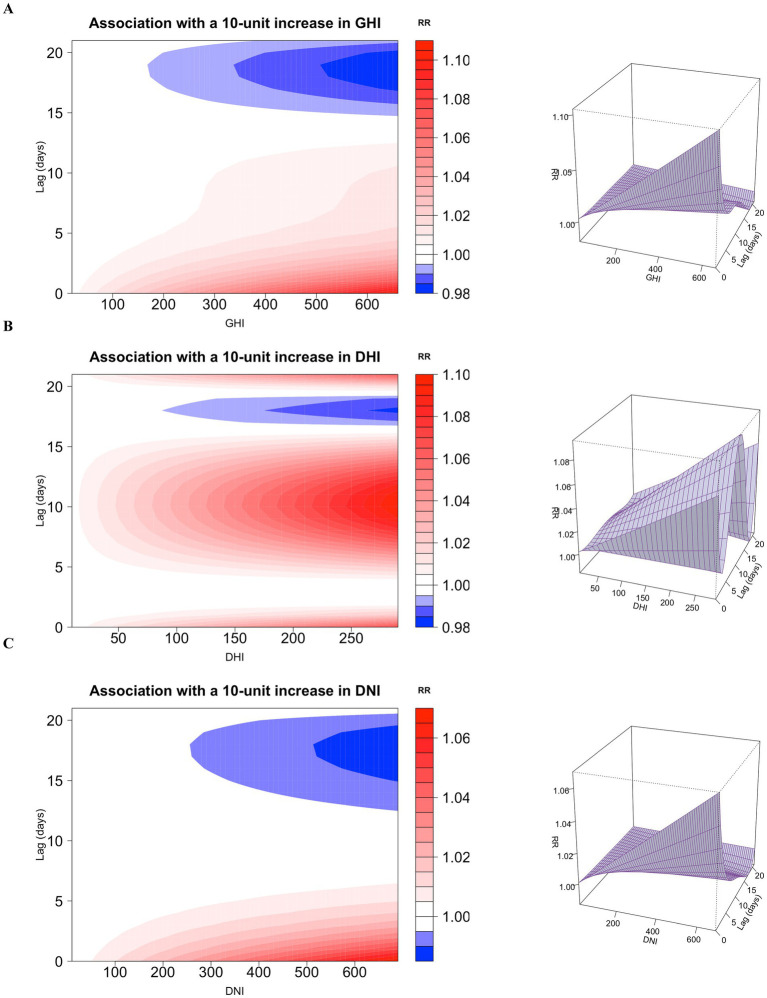
Contour plot and 3D plot illustrating the correlation between GHI **(A)**, DHI **(B)**, and DNI **(C)** and outpatient visits for meibomian gland dysfunction. GHI, global horizontal irradiance; DHI, diffuse horizontal irradiance; DNI, direct normal irradiance.

**Figure 4:**
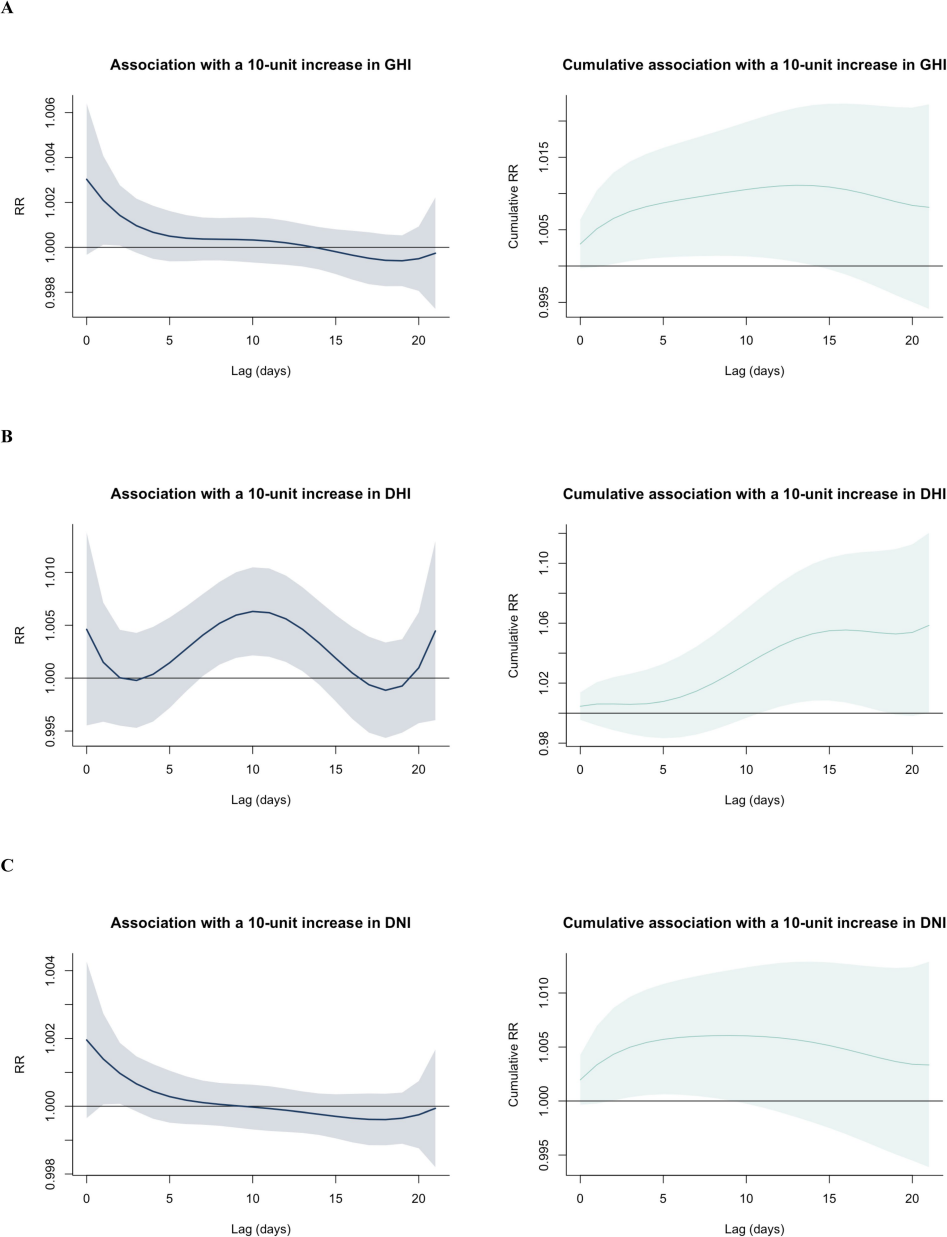
Single-day lag risks and cumulative risks of outpatient visits for meibomian gland dysfunction associated with GHI **(A)**, DHI **(B)**, and DNI **(C)**. The estimated difference in GHI, DHI, and DNI during the lag period is shown by the solid black curve in the panels arranged from top to bottom. The 95% confidence intervals (CIs) for relative risk are represented by the gray or green ribbon. GHI, global horizontal irradiance; DHI, diffuse horizontal irradiance; DNI, direct normal irradiance.

To provide a comprehensive overview of the exposure-lag-response relationship, we established 3D plots and contour plots of GHI, DHI, and DNI (Figure 3). The contour plot provides an obvious representation of the RR values over the whole range. Figure 4 shows the single-lag effects of GHI, DHI, and DNI on MGD throughout a 0–21 day lag. Significant associations are seen between GHI and DNI exposure and cases of MGD, mostly within a single lag of 1 to 2 days. DHI is associated with an increased risk of MGD. The effects tend to decrease or become statistically insignificant as the lag lengthens to 14 days. As a result, in subsequent research, we concentrated on illustrating the cumulative effect of GHI, DHI, and DNI with 0–21 day delays (Figure 4 and Supplementary Table 1), finding significant positive correlations between GHI, DHI, and DNI levels and outpatient visits for MGD with lags of 2–14 days, 11–18 days, and 2–9 days, respectively.

Table 4 displays quantitative estimations of the impact of both extremely low and extremely high levels of SR on outpatient visits for MGD across various subgroups and lag days. Overall, the estimated cumulative RRs for a lag of 0–21 days were 1.01 (95% CI: 0.99–1.02) and 1.25 (95% CI: 0.85–1.85) for extremely low and extremely high levels of GHI exposure, respectively, 1.05 (95% CI: 1.00–1.11) and 2.18 (95% CI: 1.00–4.74) for extremely low and extremely high levels of DHI exposure, respectively, and 1.01 (95% CI: 1.01–1.01) and 1.10 (95% CI: 0.84–1.44) for extremely low and extremely high levels of DNI exposure, respectively. Compared to other categories, middle-aged individuals may be more vulnerable to the negative effects of extremely high levels of GHI, DHI, and DNI exposure at 0–21 days, with respective estimated RRs of 1.76 (95% CI: 1.14–2.71), 3.47 (95% CI: 1.45–8.31), and 1.39 (95% CI: 1.03–1.87). In the season category, no statistically significant correlation was found between extremely low and extremely high levels of SR and cases of MGD.

**Table 4:** Extremely low and high level of solar radiations on the cumulative relative risk of meibomian gland dysfunction over different lag days stratified by gender, age, and season.

Variables		Extremely low level of solar radiation (1st)			Extremely high level of solar radiation (99th)		
		Lag 0–7 days	Lag 0–14 days	Lag 0–21 days	Lag 0–7 days	Lag 0–14 days	Lag 0–21 days
GHI	Total	**1.01 (1.00–1.02)**	**1.01 (1.00–1.02)**	1.01 (0.99–1.02)	**1.30 (1.04–1.63)**	**1.36 (1.00–1.84)**	1.25 (0.85–1.85)
	Gender						
	Male	**1.01 (1.00–1.02)**	**1.01 (1.00–1.03)**	**1.02 (1.00–1.03)**	1.31 (0.99–1.73)	**1.58 (1.08–2.30)**	1.61 (0.99–2.61)
	Female	**1.01 (1.00–1.02)**	**1.01 (1.00–1.02)**	1.01 (0.99–1.02)	**1.38 (1.07–1.78)**	**1.45 (1.03–2.04)**	1.32 (0.85–2.06)
	Age (years)						
	<18	1.02 (0.99–1.05)	**1.04 (1.00–1.09)**	1.04 (0.99–1.09)	1.95 (0.78–4.90)	**3.81 (1.11–13.11)**	3.15 (0.67–14.87)
	18–60	1.01 (1.00–1.02)	**1.02 (1.01–1.03)**	1.02 (1.00–1.03)	**1.48 (1.15–1.91)**	**1.75 (1.25–2.46)**	**1.76 (1.14–2.71)**
	>60	1.00 (0.99–1.01)	1.00 (0.99–1.01)	0.99 (0.98–1.01)	1.09 (0.81–1.45)	0.98 (0.66–1.44)	0.83 (0.50–1.37)
	Season						
	Warm season	1.00 (0.98–1.03)	1.00 (0.96–1.04)	0.98 (0.93–1.03)	1.05 (0.73–1.50)	0.97 (0.54–1.73)	0.76 (0.34–1.69)
	Cold season	1.00 (0.98–1.01)	1.00 (0.98–1.01)	0.98 (0.96–1.01)	1.02 (0.74–1.39)	0.96 (0.62–1.48)	0.65 (0.37–1.14)
DHI	Total	1.01 (0.99–1.04)	**1.05 (1.01–1.09)**	1.05 (1.00–1.11)	1.22 (0.82–1.81)	**2.03 (1.12–3.67)**	**2.18 (1.00–4.74)**
	Gender						
	Male	1.02 (0.99–1.06)	1.05 (1.00–1.10)	1.05 (0.99–1.12)	1.41 (0.86–2.31)	**2.17 (1.04–4.55)**	2.25 (0.86–5.92)
	Female	1.02 (0.99–1.05)	**1.06 (1.01–1.11)**	1.06 (1.00–1.12)	1.33 (0.85–2.09)	**2.33 (1.18–4.59)**	**2.44 (1.01–5.93)**
	Age (years)						
	<18	1.00 (0.90–1.11)	1.03 (0.87–1.21)	1.04 (0.84–1.28)	0.96 (0.19–4.83)	1.50 (0.13–17.32)	1.70 (0.07–42.42)
	18–60	1.03 (1.00–1.06)	**1.08 (1.03–1.13)**	**1.09 (1.02–1.15)**	**1.62 (1.04–2.54)**	**3.22 (1.65–6.30)**	**3.47 (1.45–8.31)**
	>60	1.00 (0.96–1.03)	1.01 (0.96–1.06)	1.00 (0.94–1.07)	0.93 (0.56–1.54)	1.10 (0.51–2.35)	1.08 (0.40–2.92)
	Season						
	Warm season	0.99 (0.93–1.05)	1.01 (0.91–1.11)	0.97 (0.86–1.11)	0.89 (0.55–1.44)	1.05 (0.50–2.19)	0.81 (0.30–2.19)
	Cold season	1.06 (1.00–1.12)	1.08 (0.99–1.19)	1.07 (0.94–1.22)	1.94 (0.98–3.80)	2.57 (0.87–7.59)	2.21 (0.49–9.92)
DNI	Total	**1.01 (1.01–1.01)**	**1.01 (1.01–1.01)**	**1.01 (1.01–1.01)**	**1.19 (1.01–1.39)**	1.17 (0.95–1.44)	1.10 (0.84–1.44)
	Gender						
	Male	1.00 (1.00–1.00)	1.00 (1.00–1.00)	1.00 (1.00–1.00)	1.18 (0.97–1.44)	**1.30 (1.00–1.68)**	1.33 (0.95–1.86)
	Female	1.00 (1.00–1.00)	1.00 (1.00–1.00)	1.00 (1.00–1.00)	**1.26 (1.06–1.51)**	1.26 (0.99–1.60)	1.19 (0.88–1.62)
	Age (years)						
	<18	1.00 (1.00–1.00)	1.00 (1.00–1.00)	1.00 (1.00–1.00)	1.73 (0.92–3.25)	**2.75 (1.18–6.40)**	2.55 (0.88–7.39)
	18–60	1.00 (1.00–1.00)	1.00 (1.00–1.00)	1.00 (1.00–1.00)	**1.29 (1.08–1.53)**	**1.38 (1.09–1.74)**	**1.39 (1.03–1.87)**
	>60	1.00 (1.00–1.00)	1.00 (1.00–1.00)	1.00 (1.00–1.00)	1.10 (0.90–1.35)	1.01 (0.77–1.33)	0.90 (0.63–1.28)
	Season						
	Warm season	1.00 (1.00–1.00)	1.00 (1.00–1.00)	1.00 (1.00–1.00)	1.09 (0.81–1.48)	0.97 (0.60–1.58)	0.79 (0.40–1.55)
	Cold season	1.00 (1.00–1.00)	1.00 (1.00–1.00)	1.00 (1.00–1.00)	0.97 (0.77–1.23)	0.88 (0.63–1.22)	0.66 (0.43–1.01)

GHI, global horizontal irradiance; DHI, diffuse horizontal irradiance; DNI, direct normal irradiance.

### 3.4 Subgroup analysis

**Figure 5:**
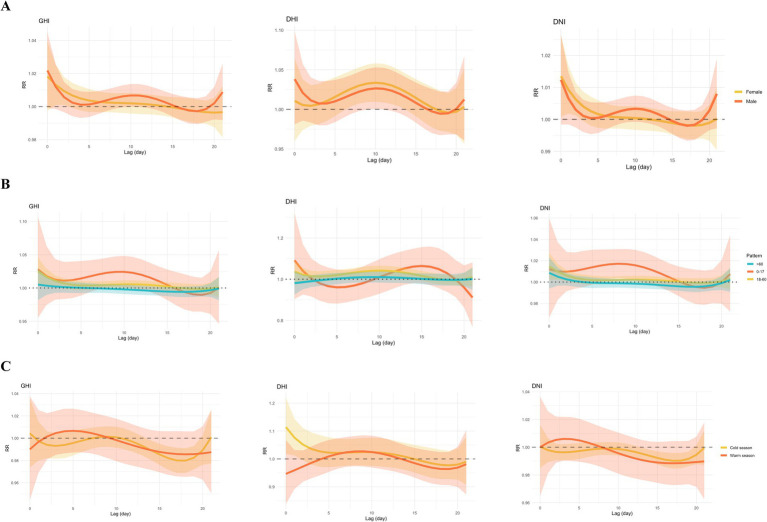
Single-day lag risks of outpatient visits for meibomian gland dysfunction associated with solar radiation stratified by gender **(A)**, age **(B)**, and season **(C)**. The 95% confidence intervals (CIs) for relative risks are represented by the colored ribbon. GHI, global horizontal irradiance; DHI, diffuse horizontal irradiance; DNI, direct normal irradiance.

We found that gender modified the associations between GHI, DHI, DNI, and MGD, with more significant effects in females than in males (Figure 5 and Supplementary Figure 3). In the female group, only DHI the maximum health effects were found on the exposure day 10 and then declined with the N-shape lag structures, whereas the lags show a decreased trend in GHI and DNI. The associations between GHI, DHI, and DNI and MGD appeared stronger in middle-aged individuals than in other groups; there were no statistically significant differences between GHI, DHI, and DNI with MGD in older individuals (Supplementary Table 2). In younger individuals, increments in the levels of GHI and DNI were positively associated with the RR of MGD with lags of 8–10 days and 6–10 days, respectively. In middle-aged individuals, increments in the levels of GHI, DHI, and DNI were positively associated with the RR of MGD with lags of 0–2 days, 6–14 days, and 0–2 days, respectively (Supplementary Table 3). According to the findings of the seasonal study, GHI and DNI are substantially associated with a decrease in outpatient visits for MGD during the cold season. Increments in the levels of GHI and DNI were negatively associated with the RR of MGD with lags of 14–19 days and 13–19 days, respectively. In general, there was a statistically significant difference in the incidence of outpatient visits for MGD between the warm and cold seasons (Supplementary Table 4).

### 3.5 Sensitivity analysis

In the sensitivity study, the relationships between SR and MGD were also robust when adjusting the df for time, air pollution factors, meteorological data, and other factors. When air pollutants and meteorological variables are changed by 2–4 df, time is changed by 6–8 df, and maximum lag days are changed by 21–23 days, and the fitted model remains robust as shown in Supplementary Figures 4–7 and Supplementary Tables 5–7. The leave-one-out sensitivity analyses showed that the estimated cumulative RRs for SR remained stable when any single air pollutant was excluded from the model (Supplementary Figure 8). As a consequence, the sensitivity study demonstrated that the models offered reliable findings and suited the data well.

### 3.6 Multiple SR exposures and MGD risk analysis

**Figure 6:**
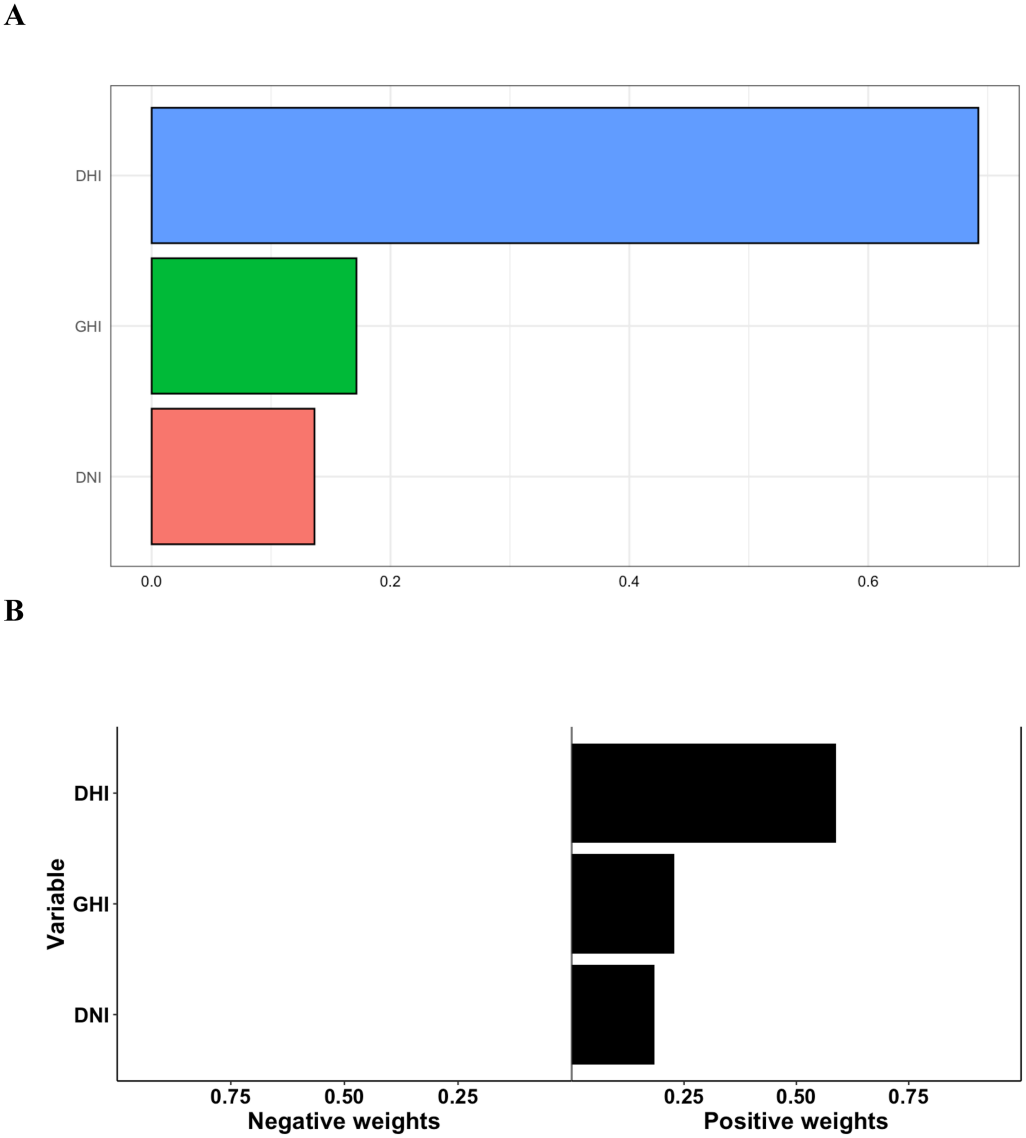
Estimated risks and weighted risk values of GHI, DHI, and DNI levels in relation to outpatient visits for meibomian gland dysfunction as calculated using the generalized weighted quantile sum regression **(A)** and g-computation on exposure quantiles **(B)**.

We used WQS regression models to investigate the association between SR combinations and the risk of MGD. A notable association was found between the risk of MGD and a quartile rise in the WQS index when assessing the positive association between the SR combinations and MGD (RR: 4.31, 95% CI: 3.26–5.36). The WQS index was dominated by DHI (0.69), followed by GHI (0.17) and DNI (0.14) as demonstrated in Figure 6A and Supplementary Figure 9. Figure 6B displays the relationship that the qgcomp model identified between the risk of MGD and combined exposure to SR. GHI, DHI, and DNI were given positive weights, and the component for DHI has the greatest absolute estimated weight (estimated weight = 0.59), whereas the component for DNI has the lowest absolute estimated weight (estimated weight = 0.18).

## 4 Discussion

The present research investigated the association between outpatient visits for MGD and exposure to SR and its components in Shanghai City from January 1, 2017, to December 31, 2023. The relationship between MGD and SR in this study varied depending on which SR indicator was taken into account. Our findings imply that increased SR exposure may have the reverse effect on MGD. Compared to other groups, females and middle-aged patients may be more vulnerable to changes in natural SR exposure, and vulnerability may increase in the cold season. The findings advance our knowledge of how sunshine affects health and offer crucial insights for clinicians and authorities looking to prevent MGD.

To our knowledge, this is the first research that demonstrates a correlation between SR exposure and MGD in a Chinese population. We established the beginning date of each case so as to correctly elucidate the impact of SR exposure on MGD, and we eliminated patients who were outpatient visit repeatedly. Furthermore, the model estimated the 21-day lagged impact of each environmental element at each decile spacing value using daily personal MGD data. The modeling approach also accounted for the periodic effects of seasonal variables on the incidence of MGD and its non-linear associations. To synthesize the overall cumulative data, we constructed a DLNM model to assess the exposure-lag-response relationships between climatic conditions and MGD onset.

Vitamin D metabolism is the potential biological mechanism linking SR exposure to MGD. A study in Japan found that the prevalence of MGD in the Japanese adult population may be negatively associated with vitamin D consumption (34). Vitamin D, an anti-inflammatory substance, has a negative association with levels of pro-inflammatory cytokines, including interleukin-6 (IL-6) and tumor necrosis factor *α* (TNF-α) (35), which were significantly increased in the tears of patients with MGD (36). Additionally, topical use of vitamin D may significantly reduce the symptoms and signs of MGD-related dry eye (37). At the same time, exposure to environment sunlight may affect the hypothalamic suprachiasmatic nuclei, which are responsible for controlling the circadian rhythm. Liu et al. found that melatonin reduces the pro-inflammatory cytokine response in the LPS-induced meibomian gland epithelial cells model, which is one of the main regulators of circadian rhythm (38). Previous studies have linked exposure to SR and UV radiation (UVR) to inflammatory and systemic immune biomarkers in older populations (39).

In a population-based study (*N* = 5,899) of residents of urban and rural regions across Bashkortostan, Russia, rural dwellers had a higher incidence of mental disorders than urban populations (40). A study in Ghana found that participants in rural areas were associated with a greater risk of poor meibomian gland health status, suggesting that working and living environments may affect people’s lifestyles (41). Overexposing the body to UVR can damage the eyes (42). Although the exact mechanism by which SR affects MGD is unidentified, data suggest that UVR can influence humoral immunity as well as the cellular immunological reaction and weaken resistance to pathogens of various organs. UVR may influence humoral immunity by inhibiting B cells’ intrinsic activity (43).

This study has several strengths. First, to the best of our knowledge, it is the largest study of the association between exposure to GHI, DHI, and DNI and the risk of MGD, with observations over 5 years in Shanghai. Second, this study’s enormous sample size guaranteed sufficient statistical power. Third, we used the WQS regression model and qgcomp model simultaneously to evaluate the joint effect of GHI, DHI, and DNI on the risk of MGD. Some limitations should also be considered when evaluating our findings. First, we lacked access to individual-level environmental exposure information, as the method employed in the present research depended on using the mean values of ambient air pollutants and meteorological factor measurements acquired from stationary monitoring sites as proxies for individual exposure. The relationship between PM_2.5_ and systemic inflammation diminished when environmental data were used, which suggests that using ambient pollution concentration as a proxy for individual exposure may not be reliable (44). To increase the reliability of the findings, more relevant research should be performed. Second, patients’ individual-level data are missing, such as social and economic status, occupation, behavioral habits, and level of education, which could impact the effects of SR; additionally, we did not collect other potential confounding factors that may be associated with MGD outpatient visits. Third, this epidemiological study on Shanghai’s population can provide only a group-level relationship, as it adopted a cross-sectional design study, which is unable to determine causal associations. Therefore, further in-depth studies are needed to investigate causal connections and underlying biological mechanisms. Fourth, a certain degree of bias could exist in the exposure estimate for the entire population due to the geographically unequal distribution of the population and air quality stations.

We propose this as a preliminary quantitative framework that captures both the non-linear dose–response pattern and the temporal dynamics of SR effects on MGD. While this model is derived from our current dataset and requires external validation with multi-center data and individual-level exposure assessment, it provides a foundation for understanding the complex relationship between SR and ocular surface health. Our findings imply a relationship between an elevated SR and an increased risk of outpatient visits for MGD, particularly for females and middle-aged adults and during the cold season. Among the three evaluated SR components, DHI was found to be important in increasing the risk of MGD. Our study raises awareness of unusual and natural environmental exposure; nevertheless, to validate our findings and elucidate the underlying biological mechanisms, more research using biomarker data in various situations and populations is required.

## Data Availability

The original contributions presented in the study are included in the article/Supplementary material, further inquiries can be directed to the corresponding author.
