# Supplementary material for: The exposure-lag-response association between solar radiation components and meibomian gland dysfunction in Shanghai, China
Source: Front Public Health. 2026 Mar 6;14:1797475. doi: 10.3389/fpubh.2026.1797475 (PMC13002615; doi:10.3389/fpubh.2026.1797475)
Supplement: Supplementary file 1 [file Table_1.docx]

Supplementary Material

# Supplementary Data

# For the exposure-response dimension, we employed a natural cubic spline with internal knots positioned at equidistant percentiles (25th, 50th, and 75th) of the solar radiation distribution, corresponding to 4 degrees of freedom (df). The df were selected by minimizing the Akaike Information Criterion (AIC) under a Poisson model, considering candidate df values ranging from 3 to 6.

# For the lag response dimension, we employed a natural cubic spline with internal knots spaced equidistantly on the logarithmic scale of the lag space (to provide greater flexibility at shorter lags), corresponding to 4 degrees of freedom (df). Based on prior studies [1, 2] and our preliminary analysis, the maximum lag was set to 21 days. The df for the lag dimension was also selected via AIC, with candidate values ranging from 3 to 6.

# The final model selected based on AIC employed df = 4 for the exposure response and df = 4 for the lag response dimension. This parameterization was consistently applied to all three solar radiation components (DHI, DNI, and GHI).

[1] S. Gu, R. Huang, J. Yang, S. Sun, Y. Xu, R. Zhang, Y. Wang, B. Lu, T. He, A. Wang, G. Bian, and Q. Wang, Exposure-lag-response association between sunlight and schizophrenia in Ningbo, China. Environ Pollut 247 (2019) 285-292.

[2] B. Vyssoki, N.D. Kapusta, N. Praschak-Rieder, G. Dorffner, and M. Willeit, Direct effect of sunshine on suicide. JAMA psychiatry 71 (2014) 1231-7.

# Supplementary Figures and Tables

## Supplementary Figures

**

**

**Supplementary Figure 1.** Boxplots showing the monthly global solar radiation in Shanghai city, during January 01, 2017 and December 31, 2023.

**
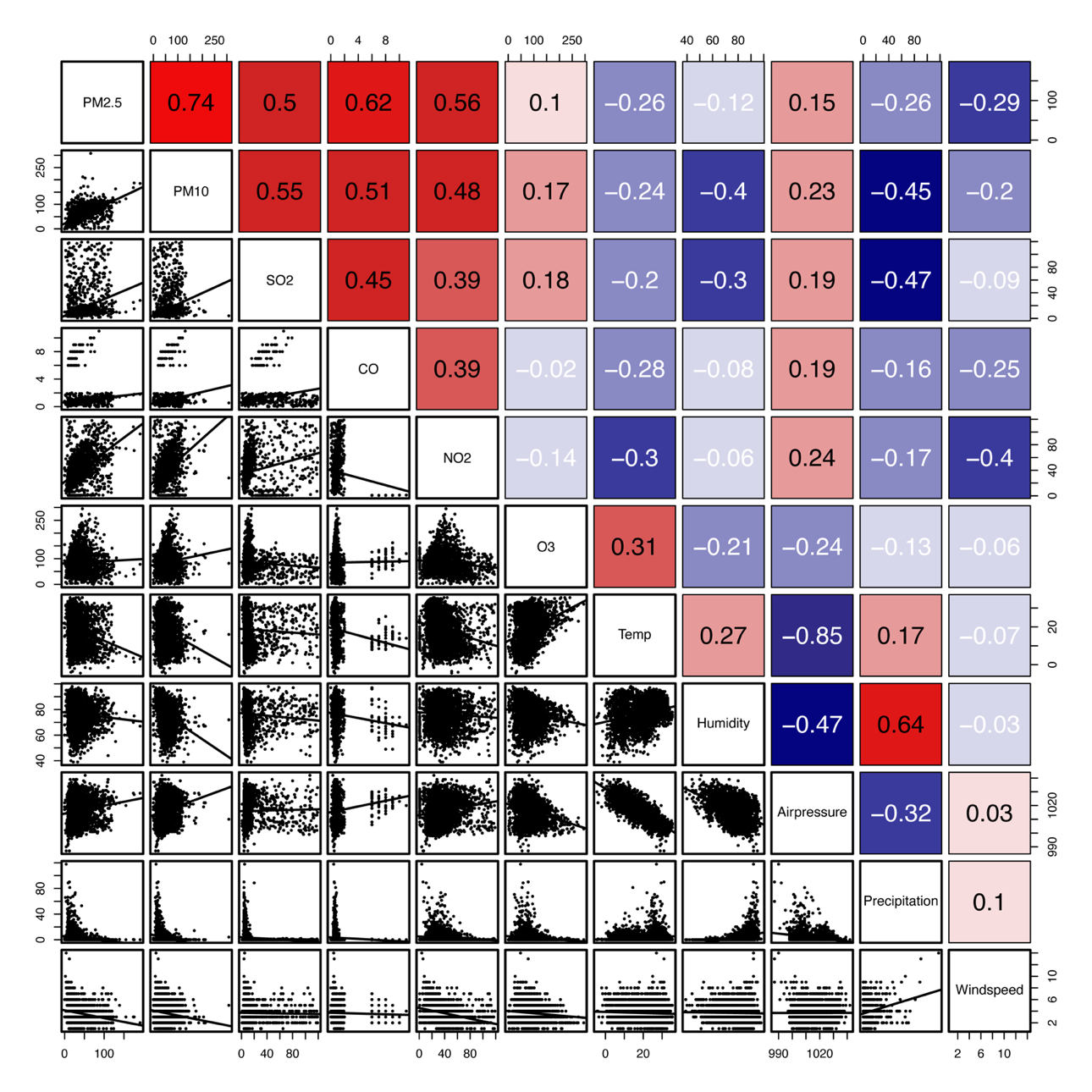
**

**Supplementary Figure 2.** Spearmans correlation coefficients between air pollutants and meteorological variables in Shanghai city, China, from January 01, 2017 to December 31, 2023.

**
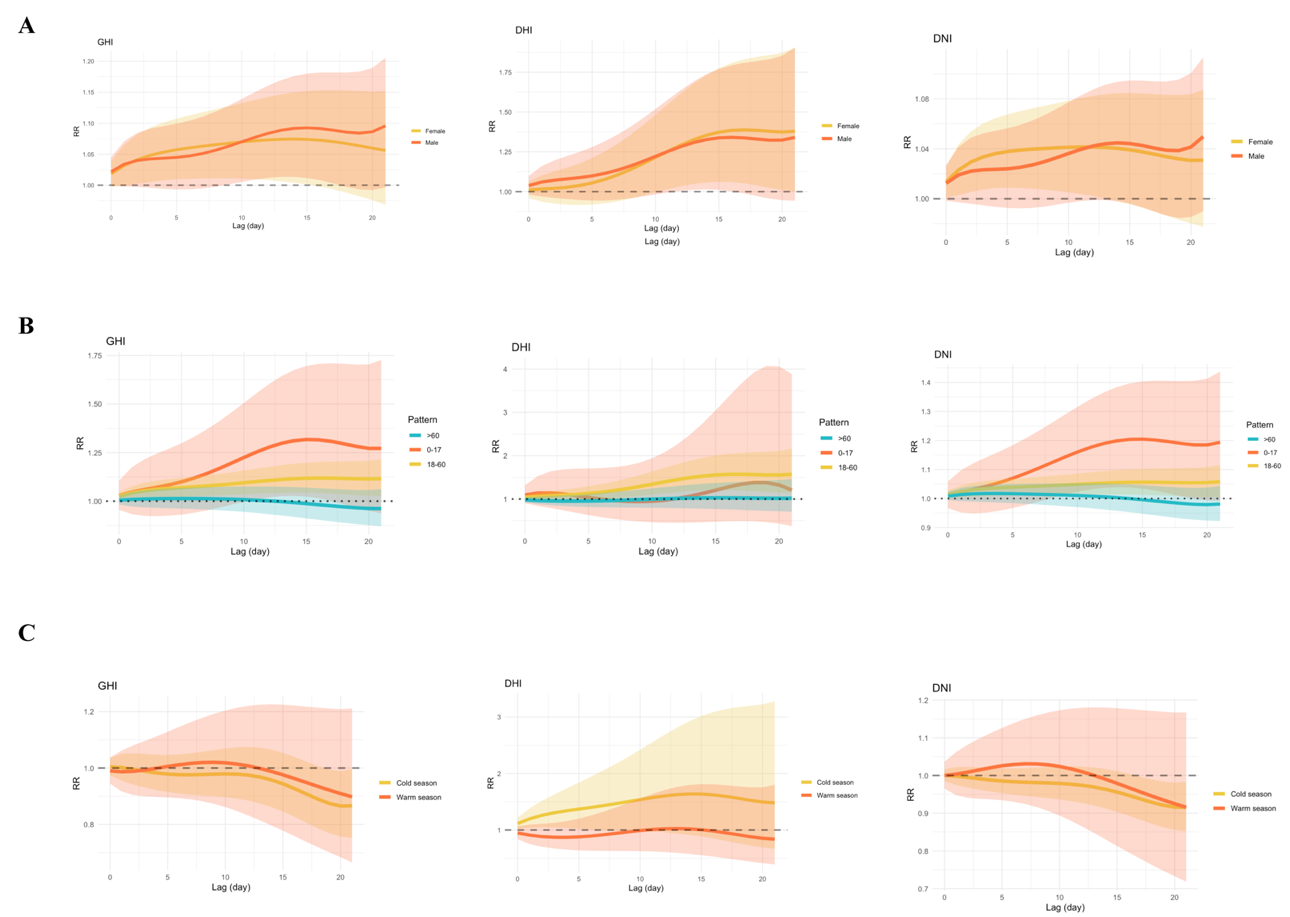
**

**Supplementary Figure 3.** Cumulative risks of outpatient visits for meibomian gland dysfunction associated with solar radiation stratified by gender (A), age (B) and season (C). The 95% confidence intervals (CIs) for relative risks are represented by the color ribbon. GHI: global horizontal irradiance; DHI: diffuse horizontal irradiance; DNI: direct normal irradiance.

**
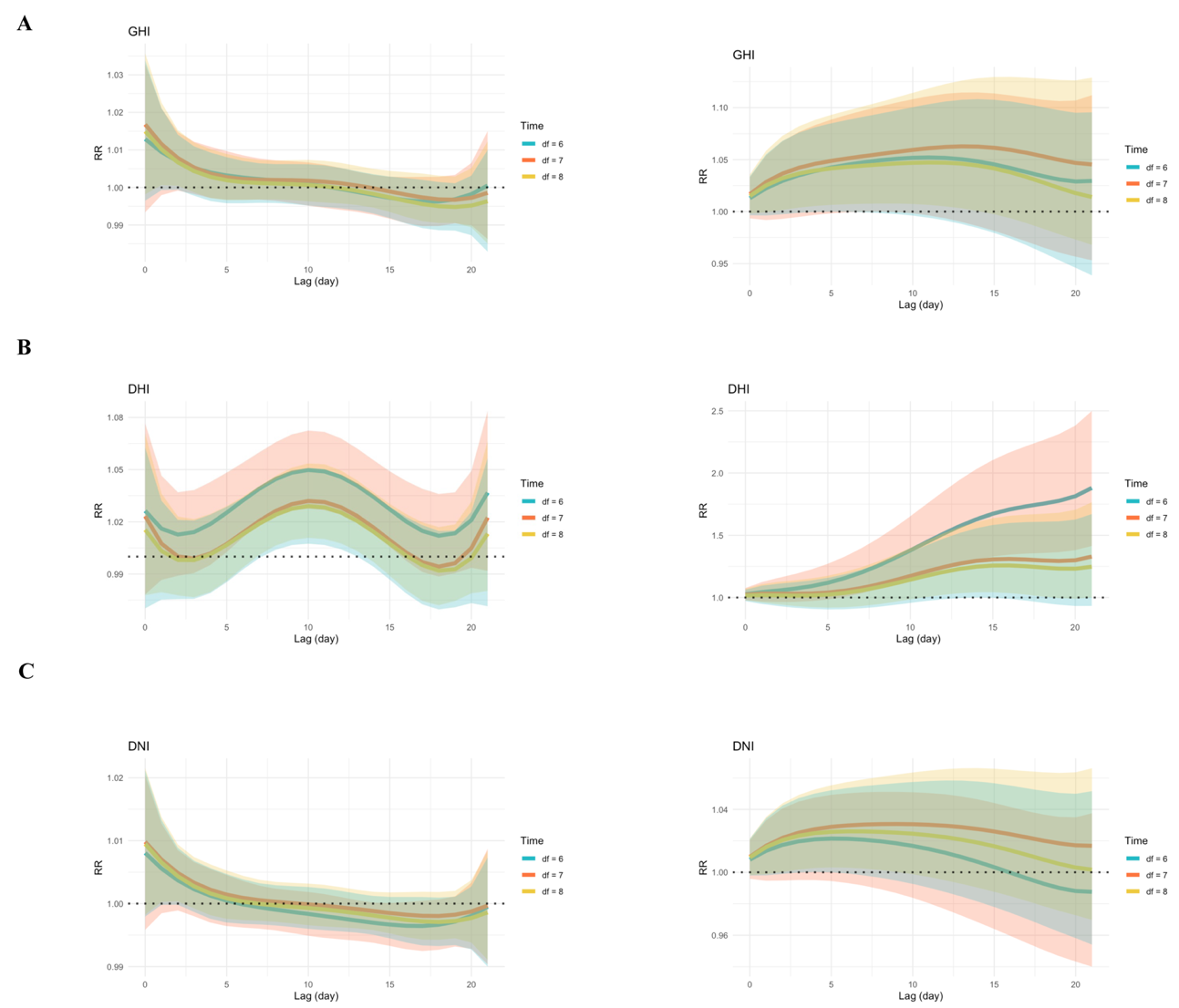
**

**Supplementary Figure 4.**The effects of GHI (A), DHI (B), and DNI (C) on meibomian gland dysfunction in the sensitivity analyses about changing the df (6-8) for time. GHI: global horizontal irradiance; DHI: diffuse horizontal irradiance; DNI: direct normal irradiance.

**
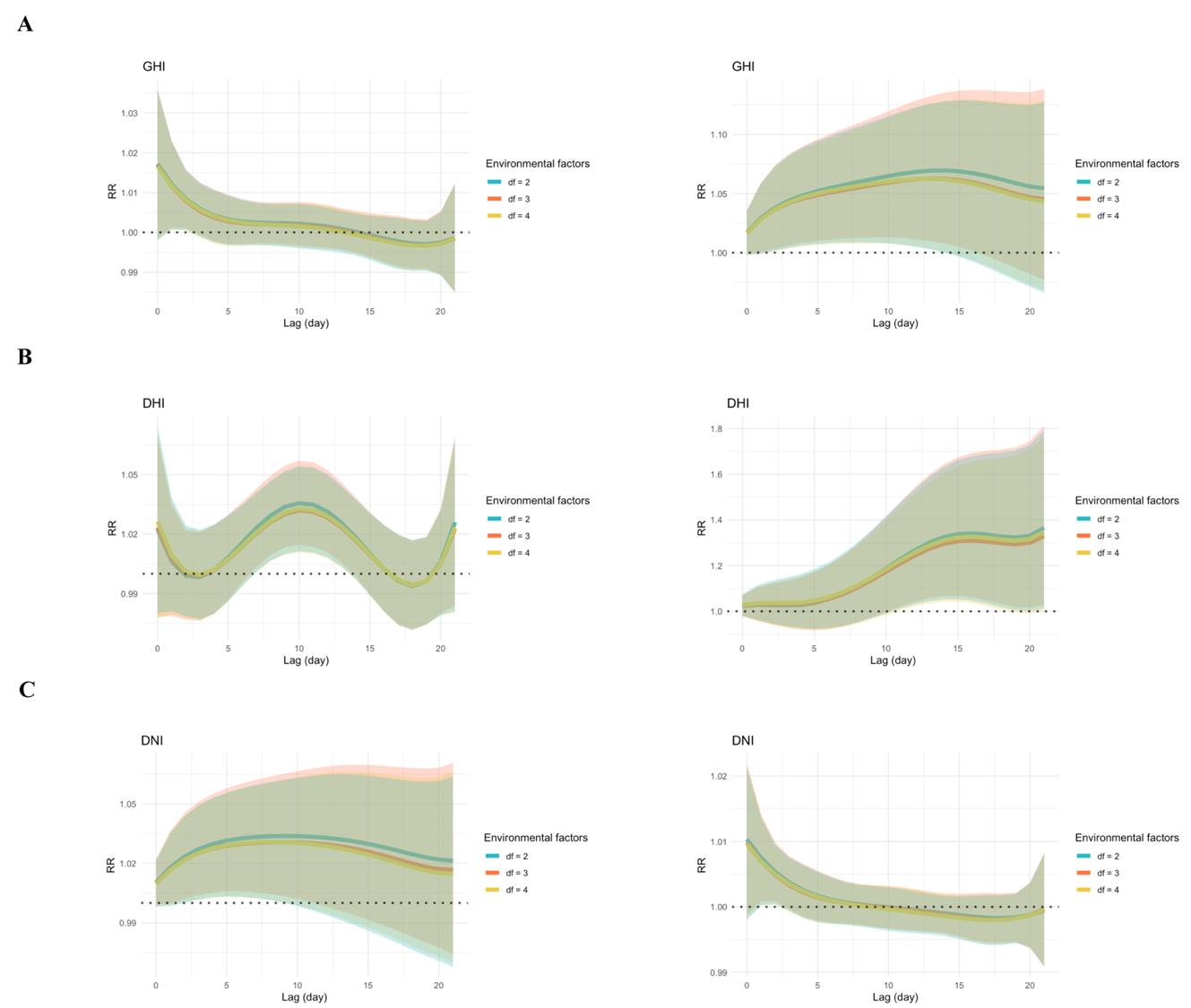
**

**Supplementary Figure 5.**The effects of GHI (A), DHI (B), and DNI (C) on meibomian gland dysfunction in the sensitivity analyses about changing the df (2-4) for meteorological factors. GHI: global horizontal irradiance; DHI: diffuse horizontal irradiance; DNI: direct normal irradiance.

**
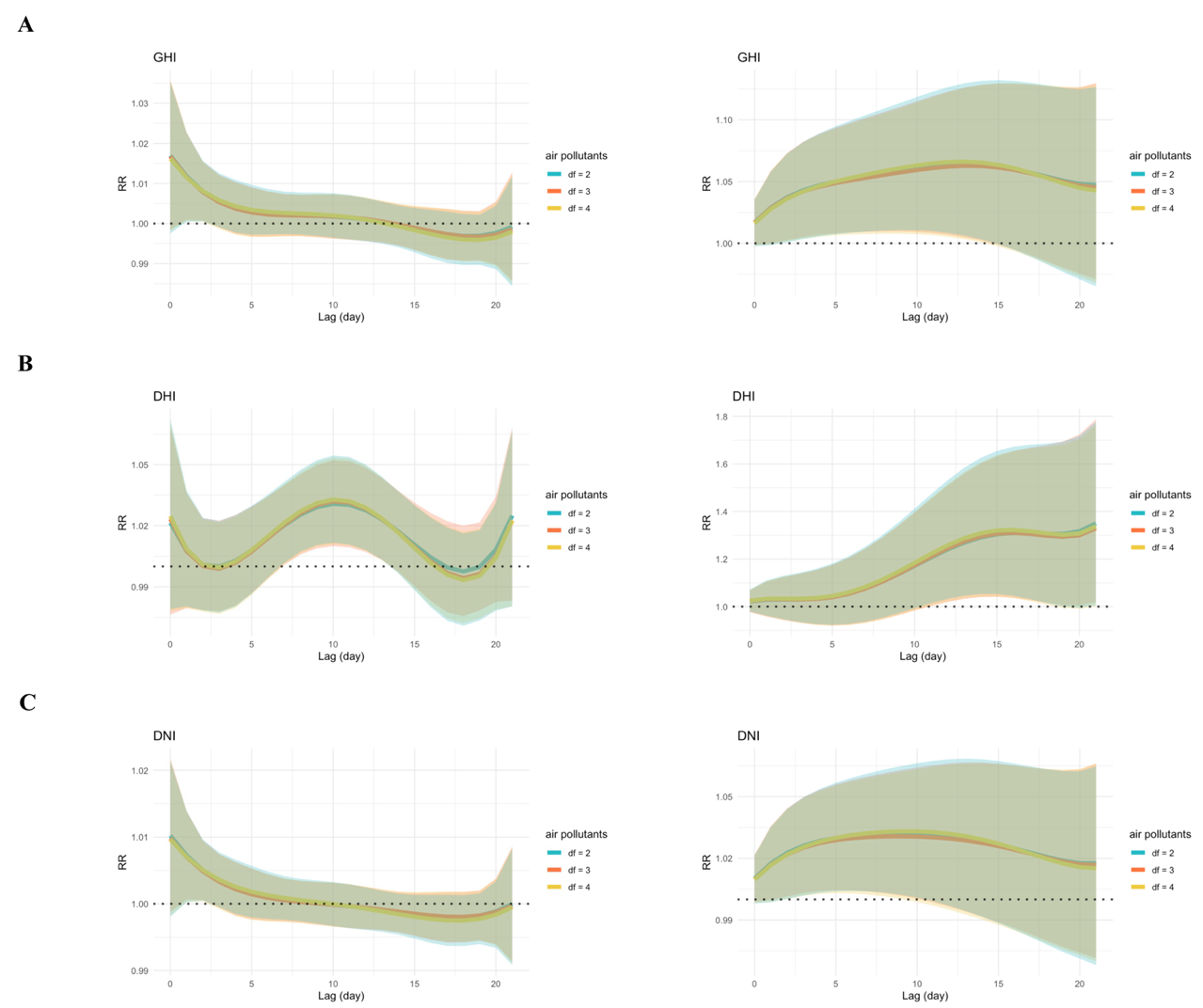
**

**Supplementary Figure 6.** The effects of GHI (A), DHI (B), and DNI (C) on meibomian gland dysfunction in the sensitivity analyses about changing the df (2-4) for air pollutants. GHI: global horizontal irradiance; DHI: diffuse horizontal irradiance; DNI: direct normal irradiance.

**
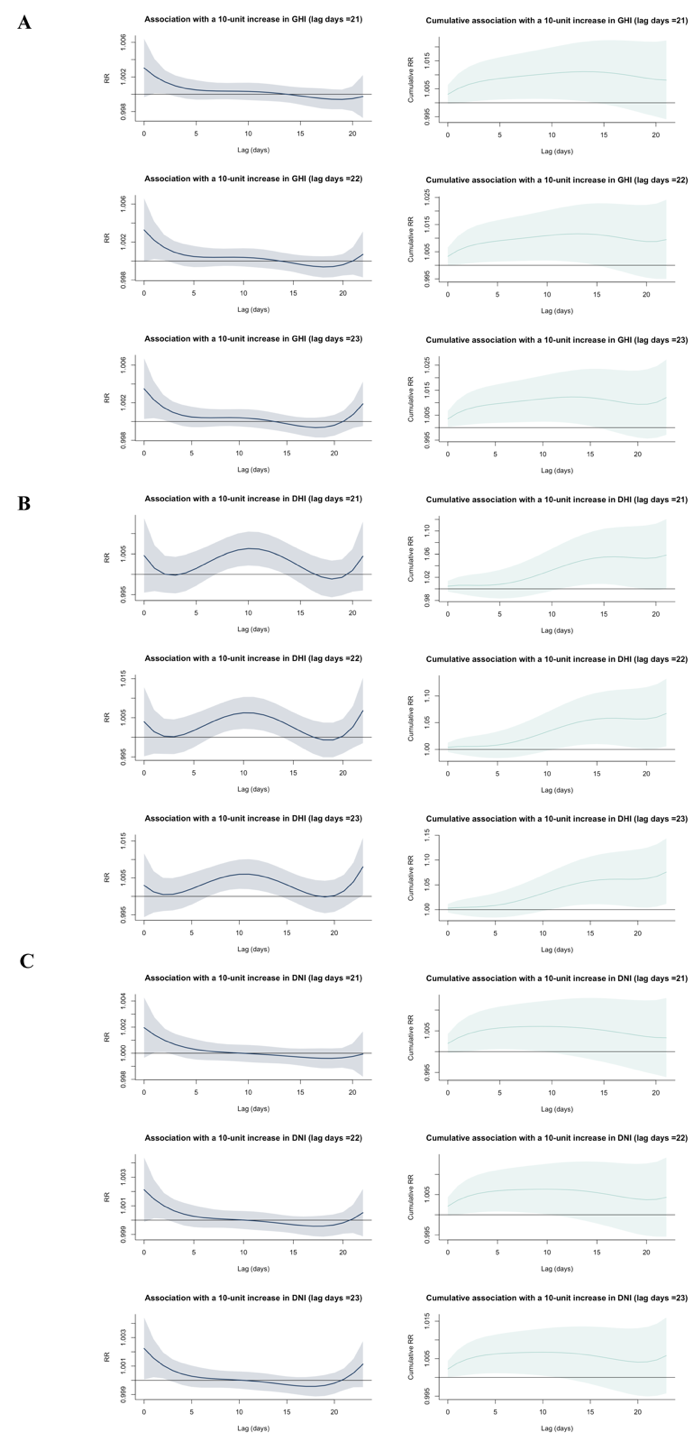
**

**Supplementary Figure 7.**The effects of GHI (A), DHI (B), and DNI (C) on meibomian gland dysfunction in the sensitivity analyses about changing the maximum lag days (21-23 days). GHI: global horizontal irradiance; DHI: diffuse horizontal irradiance; DNI: direct normal irradiance.


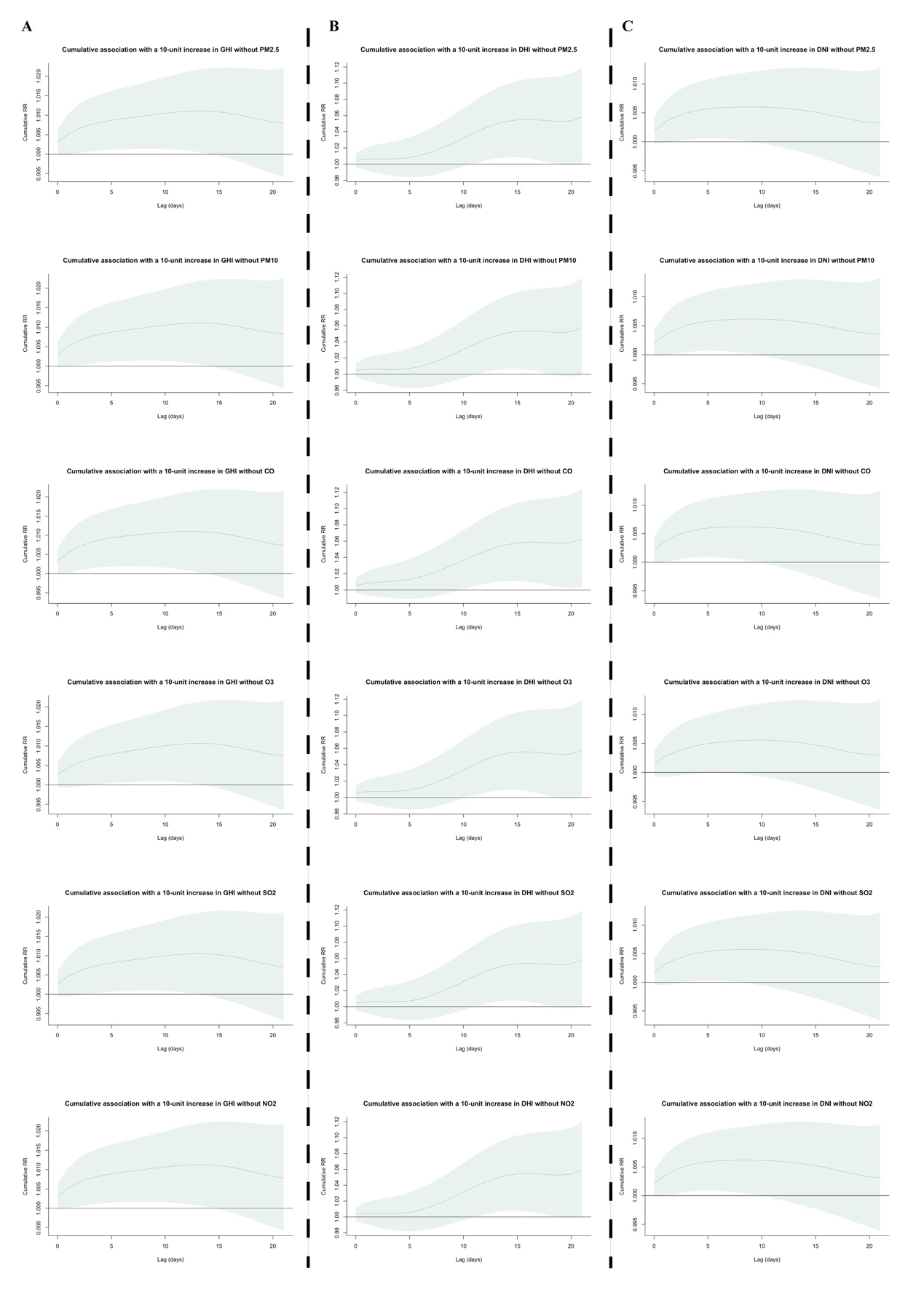


**Supplementary figure 8.** The effects of GHI (A), DHI (B), and DNI (C) on meibomian gland dysfunction in the leave-one-out sensitivity analysis. GHI: global horizontal irradiance; DHI: diffuse horizontal irradiance; DNI: direct normal irradiance.

**
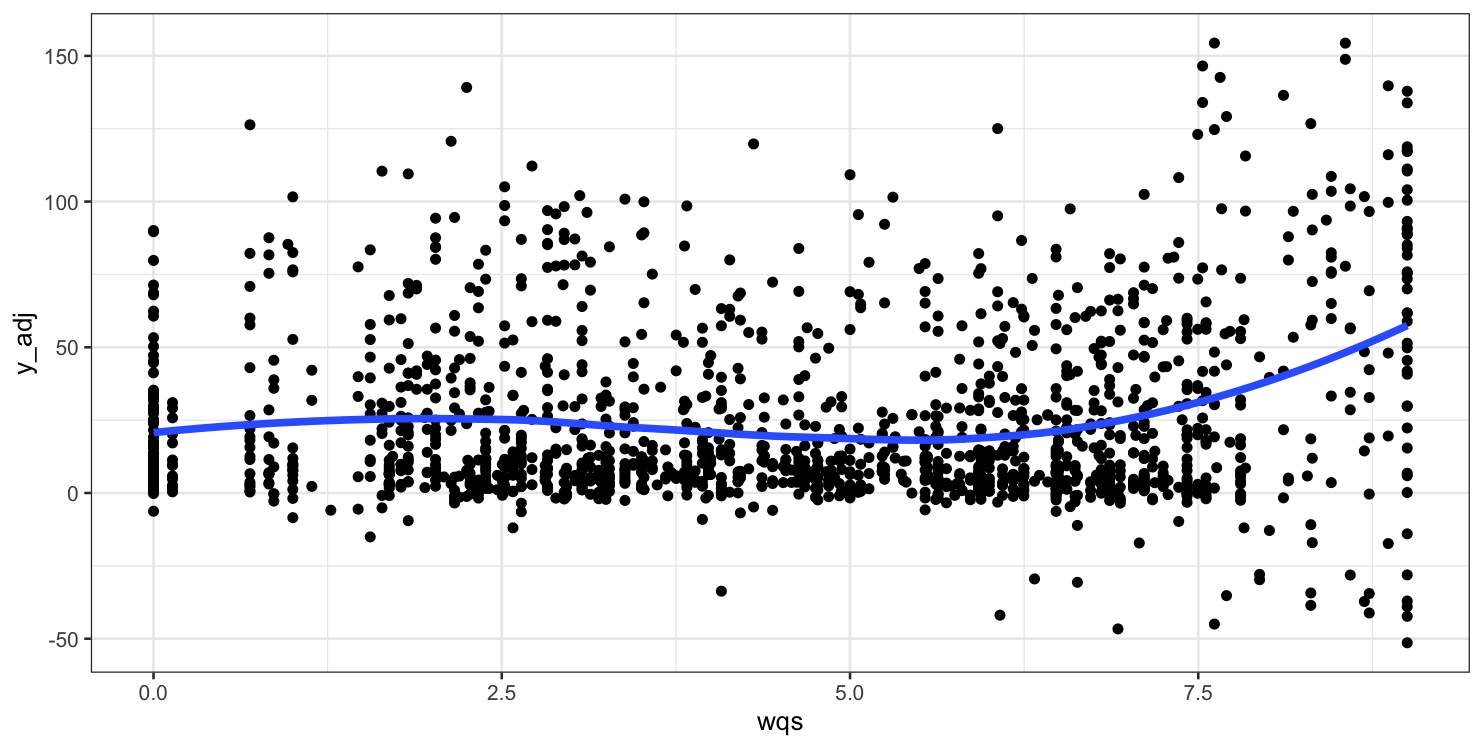
**

**Supplementary figure 9.** The plot reveals the relationship between the gWQS index and the outcome. We observe a linear and positive relationship between the mixture and the variable in a positive direction.

TABLE OF CONTENTS

**Supplementary Table 1.** Lag-specific relative risks and cumulative risks in outpatient visits for meibomian gland dysfunction associated with GHI, DHI, and DNI in the model.

**Supplementary Table 2.** Lag-specific relative risks and cumulative risks in outpatient visits for meibomian gland dysfunction associated with GHI, DHI, and DNI in different gender.

**Supplementary Table 3.** Lag-specific relative risks and cumulative risks in outpatient visits for meibomian gland dysfunction associated with GHI, DHI, and DNI in different age.

**Supplementary Table 4.** Lag-specific relative risks and cumulative risks in outpatient visits for meibomian gland dysfunction associated with GHI, DHI, and DNI in cold and warm seasons.

**Supplementary Table 5.** The sensitivity analysis of changing df for time (6–8) demonstrated the impacts of solar radiation on outpatient visits for meibomian gland dysfunction.

**Supplementary Table 6.** The sensitivity analysis of changing df for meteorological factors (2–4) demonstrated the impacts of solar radiation on outpatient visits for meibomian gland dysfunction.

**Supplementary Table 7.** The sensitivity analysis of changing df for air pollutants (2–4) demonstrated the impacts of solar radiation on outpatient visits for meibomian gland dysfunction.

**Supplementary Figure 1.** Boxplots showing the monthly global solar radiation in Shanghai city, during January 01, 2017 and December 31, 2023.

**Supplementary Figure 2.** Spearman‘s correlation coefficients between air pollutants and meteorological variables in Shanghai city, China, from January 01, 2017 to December 31, 2023.

**Supplementary Figure 3.** Cumulative risks of outpatient visits for meibomian gland dysfunction associated with solar radiation stratified by gender (A), age (B) and season (C). The 95% confidence intervals (CIs) for relative risks are represented by the color ribbon.

**Supplementary Figure 4.**The effects of GHI (A), DHI (B), and DNI (C) on meibomian gland dysfunction in the sensitivity analyses about changing the df (6-8) for time. GHI: global horizontal irradiance; DHI: diffuse horizontal irradiance; DNI: direct normal irradiance.

**Supplementary Figure 5.**The effects of GHI (A), DHI (B), and DNI (C) on meibomian gland dysfunction in the sensitivity analyses about changing the df (2-4) for meteorological factors. GHI: global horizontal irradiance; DHI: diffuse horizontal irradiance; DNI: direct normal irradiance.

**Supplementary Figure 6.**The effects of GHI (A), DHI (B), and DNI (C) on meibomian gland dysfunction in the sensitivity analyses about changing the df (2-4) for air pollutants. GHI: global horizontal irradiance; DHI: diffuse horizontal irradiance; DNI: direct normal irradiance.

**Supplementary Figure 7.**The effects of GHI (A), DHI (B), and DNI (C) on meibomian gland dysfunction in the sensitivity analyses about changing the maximum lag days (21-23 days). GHI: global horizontal irradiance; DHI: diffuse horizontal irradiance; DNI: direct normal irradiance.

**Supplementary figure 8.** The plot reveals the relationship between the gWQS index and the outcome. We observe a linear and positive relationship between the mixture and the variable in a positive direction.

**Supplementary Table 1.** Lag-specific relative risks and cumulative risks in outpatient visits for meibomian gland dysfunction associated with GHI, DHI, and DNI in the model.

|  | Lag days | Single-day lag RR (95%CI) | | | Lag days | Cumulative -day lag RR (95%CI) | | |
| --- | --- | --- | --- | --- | --- | --- | --- | --- |
|  |  | RR | LCI | UCI |  | RR | LCI | UCI |
| GHI | 0 | **1.00** | **1.00** | **1.01** | 0-0 | **1.00** | **1.00** | **1.01** |
|  | 1 | **1.00** | **1.00** | **1.00** | 0-1 | **1.01** | **1.00** | **1.01** |
|  | 2 | **1.00** | **1.00** | **1.00** | 0-2 | **1.01** | **1.00** | **1.01** |
|  | 3 | **1.00** | **1.00** | **1.00** | 0-3 | **1.01** | **1.00** | **1.01** |
|  | 4 | 1.00 | 1.00 | 1.00 | 0-4 | **1.01** | **1.00** | **1.02** |
|  | 5 | 1.00 | 1.00 | 1.00 | 0-5 | **1.01** | **1.00** | **1.02** |
|  | 6 | 1.00 | 1.00 | 1.00 | 0-6 | **1.01** | **1.00** | **1.02** |
|  | 7 | 1.00 | 1.00 | 1.00 | 0-7 | **1.01** | **1.00** | **1.02** |
|  | 8 | 1.00 | 1.00 | 1.00 | 0-8 | **1.01** | **1.00** | **1.02** |
|  | 9 | 1.00 | 1.00 | 1.00 | 0-9 | **1.01** | **1.00** | **1.02** |
|  | 10 | 1.00 | 1.00 | 1.00 | 0-10 | **1.01** | **1.00** | **1.02** |
|  | 11 | 1.00 | 1.00 | 1.00 | 0-11 | **1.01** | **1.00** | **1.02** |
|  | 12 | 1.00 | 1.00 | 1.00 | 0-12 | **1.01** | **1.00** | **1.02** |
|  | 13 | 1.00 | 1.00 | 1.00 | 0-13 | **1.01** | **1.00** | **1.02** |
|  | 14 | 1.00 | 1.00 | 1.00 | 0-14 | **1.01** | **1.00** | **1.02** |
|  | 15 | 1.00 | 1.00 | 1.00 | 0-15 | **1.01** | **1.00** | **1.02** |
|  | 16 | 1.00 | 1.00 | 1.00 | 0-16 | 1.01 | 1.00 | 1.02 |
|  | 17 | 1.00 | 1.00 | 1.00 | 0-17 | 1.01 | 1.00 | 1.02 |
|  | 18 | 1.00 | 1.00 | 1.00 | 0-18 | 1.01 | 1.00 | 1.02 |
|  | 19 | 1.00 | 1.00 | 1.00 | 0-19 | 1.01 | 1.00 | 1.02 |
|  | 20 | 1.00 | 1.00 | 1.00 | 0-20 | 1.01 | 1.00 | 1.02 |
|  | 21 | 1.00 | 1.00 | 1.00 | 0-21 | 1.01 | 0.99 | 1.02 |
| DHI | 0 | 1.00 | 1.00 | 1.01 | 0-0 | 1.00 | 1.00 | 1.01 |
|  | 1 | 1.00 | 1.00 | 1.00 | 0-1 | 1.00 | 1.00 | 1.01 |
|  | 2 | 1.00 | 1.00 | 1.00 | 0-2 | 1.00 | 0.99 | 1.01 |
|  | 3 | 1.00 | 1.00 | 1.00 | 0-3 | 1.00 | 0.99 | 1.01 |
|  | 4 | 1.00 | 1.00 | 1.00 | 0-4 | 1.00 | 0.99 | 1.01 |
|  | 5 | 1.00 | 1.00 | 1.00 | 0-5 | 1.00 | 0.99 | 1.02 |
|  | 6 | 1.00 | 1.00 | 1.00 | 0-6 | 1.01 | 0.99 | 1.02 |
|  | 7 | **1.00** | **1.00** | **1.00** | 0-7 | 1.01 | 0.99 | 1.02 |
|  | 8 | **1.00** | **1.00** | **1.01** | 0-8 | 1.01 | 0.99 | 1.03 |
|  | 9 | **1.00** | **1.00** | **1.01** | 0-9 | 1.01 | 1.00 | 1.03 |
|  | 10 | **1.00** | **1.00** | **1.01** | 0-10 | 1.02 | 1.00 | 1.03 |
|  | 11 | **1.00** | **1.00** | **1.01** | 0-11 | **1.02** | **1.00** | **1.04** |
|  | 12 | **1.00** | **1.00** | **1.01** | 0-12 | **1.02** | **1.00** | **1.04** |
|  | 13 | **1.00** | **1.00** | **1.00** | 0-13 | **1.02** | **1.00** | **1.05** |
|  | 14 | **1.00** | **1.00** | **1.00** | 0-14 | **1.03** | **1.00** | **1.05** |
|  | 15 | 1.00 | 1.00 | 1.00 | 0-15 | **1.03** | **1.00** | **1.05** |
|  | 16 | 1.00 | 1.00 | 1.00 | 0-16 | **1.03** | **1.00** | **1.05** |
|  | 17 | 1.00 | 1.00 | 1.00 | 0-17 | **1.03** | **1.00** | **1.05** |
|  | 18 | 1.00 | 1.00 | 1.00 | 0-18 | **1.03** | **1.00** | **1.05** |
|  | 19 | 1.00 | 1.00 | 1.00 | 0-19 | **1.03** | **1.00** | **1.05** |
|  | 20 | 1.00 | 1.00 | 1.00 | 0-20 | 1.03 | 1.00 | 1.06 |
|  | 21 | 1.00 | 1.00 | 1.01 | 0-21 | **1.03** | **1.00** | **1.06** |
| DNI | 0 | **1.00** | **1.00** | **1.00** | 0-0 | **1.00** | **1.00** | **1.00** |
|  | 1 | **1.00** | **1.00** | **1.00** | 0-1 | **1.00** | **1.00** | **1.00** |
|  | 2 | **1.00** | **1.00** | **1.00** | 0-2 | **1.00** | **1.00** | **1.00** |
|  | 3 | **1.00** | **1.00** | **1.00** | 0-3 | **1.00** | **1.00** | **1.01** |
|  | 4 | **1.00** | **1.00** | **1.00** | 0-4 | **1.00** | **1.00** | **1.01** |
|  | 5 | **1.00** | **1.00** | **1.00** | 0-5 | **1.00** | **1.00** | **1.01** |
|  | 6 | **1.00** | **1.00** | **1.00** | 0-6 | **1.00** | **1.00** | **1.01** |
|  | 7 | **1.00** | **1.00** | **1.00** | 0-7 | **1.00** | **1.00** | **1.01** |
|  | 8 | **1.00** | **1.00** | **1.00** | 0-8 | **1.00** | **1.00** | **1.01** |
|  | 9 | **1.00** | **1.00** | **1.00** | 0-9 | **1.00** | **1.00** | **1.01** |
|  | 10 | **1.00** | **1.00** | **1.00** | 0-10 | **1.00** | **1.00** | **1.01** |
|  | 11 | **1.00** | **1.00** | **1.00** | 0-11 | **1.00** | **1.00** | **1.01** |
|  | 12 | **1.00** | **1.00** | **1.00** | 0-12 | 1.00 | 1.00 | 1.01 |
|  | 13 | **1.00** | **1.00** | **1.00** | 0-13 | 1.00 | 1.00 | 1.01 |
|  | 14 | **1.00** | **1.00** | **1.00** | 0-14 | 1.00 | 1.00 | 1.01 |
|  | 15 | **1.00** | **1.00** | **1.00** | 0-15 | 1.00 | 1.00 | 1.01 |
|  | 16 | 1.00 | 1.00 | 1.00 | 0-16 | 1.00 | 1.00 | 1.01 |
|  | 17 | 1.00 | 1.00 | 1.00 | 0-17 | 1.00 | 1.00 | 1.01 |
|  | 18 | 1.00 | 1.00 | 1.00 | 0-18 | 1.00 | 1.00 | 1.01 |
|  | 19 | 1.00 | 1.00 | 1.00 | 0-19 | 1.00 | 1.00 | 1.01 |
|  | 20 | 1.00 | 1.00 | 1.00 | 0-20 | 1.00 | 1.00 | 1.01 |
|  | 21 | 1.00 | 1.00 | 1.00 | 0-21 | 1.00 | 1.00 | 1.01 |

Note. Statistically significant (*p* < 0.05) were labeled in bold font; RR: relative risk; UCI: upper confidence interval; LCI: lower confidence interval; DHI: diffuse horizontal irradiance; DNI: direct normal irradiance; GHI: global horizontal irradiance.

**Supplementary Table 2.** Lag-specific relative risks and cumulative risks in outpatient visits for meibomian gland dysfunction associated with GHI, DHI, and DNI in different gender.

|  | Single-day lag RR (95%CI) | | | | | | | Cumulative -day lag RR (95%CI) | | | | | | |
| --- | --- | --- | --- | --- | --- | --- | --- | --- | --- | --- | --- | --- | --- | --- |
|  |  | Female | | | Male | | |  | Female | | | Male | | |
|  | Lag days | RR | LCI | UCI | RR | LCI | UCI | Lag days | RR | LCI | UCI | RR | LCI | UCI |
| GHI | 0 | 1.02 | 1.00 | 1.04 | 1.02 | 1.00 | 1.05 | 0-0 | 1.02 | 1.00 | 1.04 | 1.02 | 1.00 | 1.05 |
|  | 1 | **1.01** | **1.00** | **1.03** | 1.01 | 1.00 | 1.03 | 0-1 | 1.03 | 1.00 | 1.07 | 1.03 | 1.00 | 1.07 |
|  | 2 | **1.01** | **1.00** | **1.02** | 1.01 | 1.00 | 1.02 | 0-2 | **1.04** | **1.00** | **1.08** | 1.04 | 1.00 | 1.08 |
|  | 3 | 1.01 | 1.00 | 1.01 | 1.00 | 0.99 | 1.01 | 0-3 | **1.05** | **1.01** | **1.09** | 1.04 | 1.00 | 1.09 |
|  | 4 | 1.01 | 1.00 | 1.01 | 1.00 | 0.99 | 1.01 | 0-4 | **1.05** | **1.01** | **1.10** | 1.04 | 0.99 | 1.10 |
|  | 5 | 1.00 | 1.00 | 1.01 | 1.00 | 0.99 | 1.01 | 0-5 | **1.06** | **1.01** | **1.11** | 1.05 | 0.99 | 1.10 |
|  | 6 | 1.00 | 1.00 | 1.01 | 1.00 | 1.00 | 1.01 | 0-6 | **1.06** | **1.01** | **1.11** | 1.05 | 0.99 | 1.10 |
|  | 7 | 1.00 | 1.00 | 1.01 | 1.00 | 1.00 | 1.01 | 0-7 | **1.06** | **1.01** | **1.12** | 1.05 | 1.00 | 1.11 |
|  | 8 | 1.00 | 1.00 | 1.01 | 1.01 | 1.00 | 1.01 | 0-8 | **1.07** | **1.01** | **1.12** | 1.06 | 1.00 | 1.12 |
|  | 9 | 1.00 | 1.00 | 1.01 | **1.01** | **1.00** | **1.01** | 0-9 | **1.07** | **1.01** | **1.13** | **1.06** | **1.00** | **1.13** |
|  | 10 | 1.00 | 1.00 | 1.01 | **1.01** | **1.00** | **1.01** | 0-10 | **1.07** | **1.01** | **1.13** | **1.07** | **1.01** | **1.14** |
|  | 11 | 1.00 | 1.00 | 1.01 | **1.01** | **1.00** | **1.01** | 0-11 | **1.07** | **1.01** | **1.14** | **1.08** | **1.01** | **1.15** |
|  | 12 | 1.00 | 1.00 | 1.01 | 1.01 | 1.00 | 1.01 | 0-12 | **1.07** | **1.01** | **1.14** | **1.08** | **1.01** | **1.16** |
|  | 13 | 1.00 | 1.00 | 1.01 | 1.01 | 1.00 | 1.01 | 0-13 | **1.07** | **1.01** | **1.15** | **1.09** | **1.01** | **1.17** |
|  | 14 | 1.00 | 0.99 | 1.01 | 1.00 | 1.00 | 1.01 | 0-14 | **1.07** | **1.00** | **1.15** | **1.09** | **1.01** | **1.18** |
|  | 15 | 1.00 | 0.99 | 1.01 | 1.00 | 0.99 | 1.01 | 0-15 | **1.07** | **1.00** | **1.15** | **1.09** | **1.01** | **1.18** |
|  | 16 | 1.00 | 0.99 | 1.01 | 1.00 | 0.99 | 1.01 | 0-16 | 1.07 | 1.00 | 1.15 | **1.09** | **1.01** | **1.18** |
|  | 17 | 1.00 | 0.99 | 1.01 | 1.00 | 0.99 | 1.01 | 0-17 | 1.07 | 0.99 | 1.15 | **1.09** | **1.00** | **1.18** |
|  | 18 | 1.00 | 0.99 | 1.00 | 1.00 | 0.99 | 1.01 | 0-18 | 1.07 | 0.99 | 1.15 | 1.09 | 1.00 | 1.18 |
|  | 19 | 1.00 | 0.99 | 1.00 | 1.00 | 0.99 | 1.01 | 0-19 | 1.06 | 0.98 | 1.15 | 1.08 | 0.99 | 1.18 |
|  | 20 | 1.00 | 0.99 | 1.01 | 1.00 | 0.99 | 1.01 | 0-20 | 1.06 | 0.98 | 1.15 | 1.09 | 0.99 | 1.19 |
|  | 21 | 1.00 | 0.98 | 1.01 | 1.01 | 0.99 | 1.03 | 0-21 | 1.06 | 0.97 | 1.15 | 1.10 | 1.00 | 1.21 |
| DHI | 0 | 1.01 | 0.96 | 1.06 | 1.04 | 0.98 | 1.10 | 0-0 | 1.01 | 0.96 | 1.06 | 1.04 | 0.98 | 1.10 |
|  | 1 | 1.01 | 0.97 | 1.04 | 1.02 | 0.99 | 1.06 | 0-1 | 1.02 | 0.94 | 1.10 | 1.06 | 0.97 | 1.16 |
|  | 2 | 1.00 | 0.98 | 1.03 | 1.01 | 0.98 | 1.04 | 0-2 | 1.02 | 0.93 | 1.13 | 1.07 | 0.96 | 1.20 |
|  | 3 | 1.01 | 0.98 | 1.03 | 1.01 | 0.98 | 1.04 | 0-3 | 1.03 | 0.92 | 1.15 | 1.08 | 0.95 | 1.22 |
|  | 4 | 1.01 | 0.99 | 1.04 | 1.01 | 0.98 | 1.04 | 0-4 | 1.04 | 0.92 | 1.18 | 1.09 | 0.95 | 1.25 |
|  | 5 | 1.02 | 0.99 | 1.04 | 1.01 | 0.98 | 1.04 | 0-5 | 1.06 | 0.92 | 1.21 | 1.10 | 0.94 | 1.28 |
|  | 6 | 1.02 | 1.00 | 1.05 | 1.01 | 0.99 | 1.04 | 0-6 | 1.08 | 0.93 | 1.25 | 1.12 | 0.94 | 1.32 |
|  | 7 | **1.03** | **1.00** | **1.05** | 1.02 | 0.99 | 1.04 | 0-7 | 1.11 | 0.94 | 1.30 | 1.14 | 0.95 | 1.36 |
|  | 8 | **1.03** | **1.01** | **1.05** | 1.02 | 1.00 | 1.05 | 0-8 | 1.14 | 0.96 | 1.36 | 1.16 | 0.96 | 1.41 |
|  | 9 | **1.03** | **1.01** | **1.06** | **1.03** | **1.00** | **1.05** | 0-9 | 1.18 | 0.98 | 1.42 | 1.19 | 0.97 | 1.46 |
|  | 10 | **1.03** | **1.01** | **1.06** | **1.03** | **1.00** | **1.05** | 0-10 | 1.22 | 1.00 | 1.48 | 1.22 | 0.98 | 1.52 |
|  | 11 | **1.03** | **1.01** | **1.06** | **1.03** | **1.00** | **1.05** | 0-11 | 1.26 | 1.02 | 1.55 | 1.25 | 1.00 | 1.58 |
|  | 12 | **1.03** | **1.01** | **1.05** | 1.02 | 1.00 | 1.05 | 0-12 | **1.29** | **1.03** | **1.62** | **1.28** | **1.01** | **1.64** |
|  | 13 | **1.03** | **1.00** | **1.05** | 1.02 | 1.00 | 1.05 | 0-13 | **1.33** | **1.05** | **1.68** | **1.31** | **1.01** | **1.69** |
|  | 14 | 1.02 | 1.00 | 1.04 | 1.01 | 0.99 | 1.04 | 0-14 | **1.35** | **1.06** | **1.73** | **1.33** | **1.01** | **1.74** |
|  | 15 | 1.02 | 0.99 | 1.04 | 1.01 | 0.98 | 1.03 | 0-15 | **1.37** | **1.06** | **1.78** | **1.34** | **1.01** | **1.77** |
|  | 16 | 1.01 | 0.98 | 1.03 | 1.00 | 0.98 | 1.03 | 0-16 | **1.39** | **1.06** | **1.81** | **1.34** | **1.00** | **1.79** |
|  | 17 | 1.00 | 0.98 | 1.03 | 1.00 | 0.97 | 1.03 | 0-17 | **1.39** | **1.05** | **1.83** | 1.34 | 0.99 | 1.81 |
|  | 18 | 1.00 | 0.97 | 1.02 | 0.99 | 0.97 | 1.02 | 0-18 | **1.39** | **1.04** | **1.85** | 1.33 | 0.97 | 1.82 |
|  | 19 | 1.00 | 0.97 | 1.02 | 1.00 | 0.97 | 1.02 | 0-19 | **1.38** | **1.02** | **1.86** | 1.32 | 0.96 | 1.83 |
|  | 20 | 1.00 | 0.97 | 1.03 | 1.00 | 0.97 | 1.03 | 0-20 | **1.38** | **1.01** | **1.87** | 1.32 | 0.95 | 1.85 |
|  | 21 | 1.00 | 0.96 | 1.05 | 1.01 | 0.96 | 1.07 | 0-21 | 1.38 | 1.00 | 1.90 | 1.34 | 0.94 | 1.91 |
| DNI | 0 | **1.01** | **1.00** | **1.03** | 1.01 | 1.00 | 1.03 | 0-0 | **1.01** | **1.00** | **1.03** | 1.01 | 1.00 | 1.03 |
|  | 1 | **1.01** | **1.00** | **1.02** | 1.01 | 1.00 | 1.02 | 0-1 | **1.02** | **1.00** | **1.04** | 1.02 | 1.00 | 1.04 |
|  | 2 | **1.01** | **1.00** | **1.01** | 1.00 | 1.00 | 1.01 | 0-2 | **1.03** | **1.01** | **1.05** | 1.02 | 1.00 | 1.05 |
|  | 3 | 1.00 | 1.00 | 1.01 | 1.00 | 1.00 | 1.01 | 0-3 | **1.03** | **1.01** | **1.06** | 1.02 | 1.00 | 1.05 |
|  | 4 | 1.00 | 1.00 | 1.01 | 1.00 | 1.00 | 1.01 | 0-4 | **1.04** | **1.01** | **1.06** | 1.02 | 0.99 | 1.05 |
|  | 5 | 1.00 | 1.00 | 1.01 | 1.00 | 1.00 | 1.01 | 0-5 | **1.04** | **1.01** | **1.07** | 1.02 | 0.99 | 1.06 |
|  | 6 | 1.00 | 1.00 | 1.01 | 1.00 | 1.00 | 1.01 | 0-6 | **1.04** | **1.01** | **1.07** | 1.03 | 0.99 | 1.06 |
|  | 7 | 1.00 | 1.00 | 1.00 | 1.00 | 1.00 | 1.01 | 0-7 | **1.04** | **1.01** | **1.07** | 1.03 | 0.99 | 1.06 |
|  | 8 | 1.00 | 1.00 | 1.00 | 1.00 | 1.00 | 1.01 | 0-8 | **1.04** | **1.01** | **1.07** | 1.03 | 0.99 | 1.07 |
|  | 9 | 1.00 | 1.00 | 1.00 | 1.00 | 1.00 | 1.01 | 0-9 | **1.04** | **1.01** | **1.08** | 1.03 | 1.00 | 1.07 |
|  | 10 | 1.00 | 1.00 | 1.00 | 1.00 | 1.00 | 1.01 | 0-10 | **1.04** | **1.01** | **1.08** | 1.04 | 1.00 | 1.08 |
|  | 11 | 1.00 | 1.00 | 1.00 | 1.00 | 1.00 | 1.01 | 0-11 | **1.04** | **1.00** | **1.08** | 1.04 | 1.00 | 1.08 |
|  | 12 | 1.00 | 1.00 | 1.00 | 1.00 | 1.00 | 1.01 | 0-12 | **1.04** | **1.00** | **1.08** | 1.04 | 1.00 | 1.09 |
|  | 13 | 1.00 | 1.00 | 1.00 | 1.00 | 1.00 | 1.01 | 0-13 | **1.04** | **1.00** | **1.08** | 1.04 | 1.00 | 1.09 |
|  | 14 | 1.00 | 1.00 | 1.00 | 1.00 | 1.00 | 1.00 | 0-14 | 1.04 | 1.00 | 1.08 | 1.05 | 1.00 | 1.09 |
|  | 15 | 1.00 | 1.00 | 1.00 | 1.00 | 1.00 | 1.00 | 0-15 | 1.04 | 1.00 | 1.09 | 1.04 | 1.00 | 1.10 |
|  | 16 | 1.00 | 0.99 | 1.00 | 1.00 | 0.99 | 1.00 | 0-16 | 1.04 | 0.99 | 1.08 | 1.04 | 0.99 | 1.10 |
|  | 17 | 1.00 | 0.99 | 1.00 | 1.00 | 0.99 | 1.00 | 0-17 | 1.04 | 0.99 | 1.08 | 1.04 | 0.99 | 1.09 |
|  | 18 | 1.00 | 0.99 | 1.00 | 1.00 | 0.99 | 1.00 | 0-18 | 1.03 | 0.99 | 1.08 | 1.04 | 0.99 | 1.09 |
|  | 19 | 1.00 | 0.99 | 1.00 | 1.00 | 1.00 | 1.00 | 0-19 | 1.03 | 0.98 | 1.08 | 1.04 | 0.99 | 1.10 |
|  | 20 | 1.00 | 0.99 | 1.01 | 1.00 | 1.00 | 1.01 | 0-20 | 1.03 | 0.98 | 1.08 | 1.04 | 0.99 | 1.10 |
|  | 21 | 1.00 | 0.99 | 1.01 | 1.01 | 1.00 | 1.02 | 0-21 | 1.03 | 0.98 | 1.09 | 1.05 | 0.99 | 1.11 |

Note. Statistically significant (*p* < 0.05) were labeled in bold font; RR: relative risk; UCI: upper confidence interval; LCI: lower confidence interval; DHI: diffuse horizontal irradiance; DNI: direct normal irradiance; GHI: global horizontal irradiance.

**Supplementary Table 3.** Lag-specific relative risks and cumulative risks in outpatient visits for meibomian gland dysfunction associated with GHI, DHI, and DNI in different age.

|  | Single-day lag RR (95%CI) | | | | | | | | | | Cumulative -day lag RR (95%CI) | | | | | | | | | |
| --- | --- | --- | --- | --- | --- | --- | --- | --- | --- | --- | --- | --- | --- | --- | --- | --- | --- | --- | --- | --- |
|  |  | <18 | | | 18-59 | | | >59 | | |  | <18 | | | 18-59 | | | >59 | | |
|  | Lag days | RR | LCI | UCI | RR | LCI | UCI | RR | LCI | UCI | Lag days | RR | LCI | UCI | RR | LCI | UCI | RR | LCI | UCI |
| GHI | 0 | 1.03 | 0.96 | 1.11 | 1.03 | 1.00 | 1.05 | 1.01 | 0.98 | 1.03 | 0-0 | 1.03 | 0.96 | 1.11 | **1.03** | **1.00** | **1.05** | 1.01 | 0.98 | 1.03 |
|  | 1 | 1.02 | 0.98 | 1.06 | **1.02** | **1.00** | **1.03** | 1.00 | 0.99 | 1.02 | 0-1 | 1.05 | 0.93 | 1.17 | **1.04** | **1.01** | **1.08** | 1.01 | 0.97 | 1.05 |
|  | 2 | 1.01 | 0.98 | 1.04 | **1.01** | **1.00** | **1.02** | 1.00 | 0.99 | 1.01 | 0-2 | 1.06 | 0.92 | 1.22 | **1.05** | **1.01** | **1.09** | 1.01 | 0.97 | 1.06 |
|  | 3 | 1.01 | 0.98 | 1.04 | **1.01** | **1.00** | **1.01** | 1.00 | 0.99 | 1.01 | 0-3 | 1.07 | 0.92 | 1.24 | **1.06** | **1.02** | **1.11** | 1.01 | 0.97 | 1.06 |
|  | 4 | 1.01 | 0.99 | 1.04 | 1.01 | 1.00 | 1.01 | 1.00 | 0.99 | 1.01 | 0-4 | 1.08 | 0.93 | 1.27 | **1.07** | **1.02** | **1.11** | 1.01 | 0.96 | 1.07 |
|  | 5 | 1.01 | 0.99 | 1.04 | 1.00 | 1.00 | 1.01 | 1.00 | 0.99 | 1.01 | 0-5 | 1.10 | 0.93 | 1.30 | **1.07** | **1.02** | **1.12** | 1.01 | 0.96 | 1.07 |
|  | 6 | 1.02 | 0.99 | 1.04 | 1.00 | 1.00 | 1.01 | 1.00 | 0.99 | 1.01 | 0-6 | 1.12 | 0.94 | 1.33 | **1.07** | **1.02** | **1.13** | 1.01 | 0.96 | 1.07 |
|  | 7 | 1.02 | 1.00 | 1.04 | 1.00 | 1.00 | 1.01 | 1.00 | 0.99 | 1.01 | 0-7 | 1.14 | 0.95 | 1.37 | **1.08** | **1.03** | **1.13** | 1.01 | 0.96 | 1.07 |
|  | 8 | **1.02** | **1.00** | **1.05** | 1.01 | 1.00 | 1.01 | 1.00 | 0.99 | 1.01 | 0-8 | 1.17 | 0.97 | 1.41 | **1.08** | **1.03** | **1.14** | 1.01 | 0.95 | 1.07 |
|  | 9 | **1.02** | **1.00** | **1.05** | 1.01 | 1.00 | 1.01 | 1.00 | 0.99 | 1.01 | 0-9 | 1.20 | 0.98 | 1.46 | **1.09** | **1.03** | **1.15** | 1.01 | 0.95 | 1.08 |
|  | 10 | **1.02** | **1.00** | **1.05** | 1.01 | 1.00 | 1.01 | 1.00 | 0.99 | 1.01 | 0-10 | 1.23 | 1.00 | 1.51 | **1.10** | **1.04** | **1.16** | 1.01 | 0.95 | 1.08 |
|  | 11 | 1.02 | 1.00 | 1.05 | 1.01 | 1.00 | 1.01 | 1.00 | 0.99 | 1.00 | 0-11 | **1.25** | **1.01** | **1.56** | **1.10** | **1.04** | **1.17** | 1.01 | 0.94 | 1.08 |
|  | 12 | 1.02 | 1.00 | 1.04 | 1.01 | 1.00 | 1.01 | 1.00 | 0.99 | 1.00 | 0-12 | **1.28** | **1.02** | **1.60** | **1.11** | **1.04** | **1.18** | 1.00 | 0.93 | 1.08 |
|  | 13 | 1.02 | 0.99 | 1.04 | 1.01 | 1.00 | 1.01 | 1.00 | 0.99 | 1.00 | 0-13 | **1.30** | **1.03** | **1.64** | **1.11** | **1.04** | **1.19** | 1.00 | 0.93 | 1.08 |
|  | 14 | 1.01 | 0.99 | 1.03 | 1.00 | 1.00 | 1.01 | 1.00 | 0.99 | 1.00 | 0-14 | **1.31** | **1.03** | **1.68** | **1.12** | **1.04** | **1.19** | 0.99 | 0.92 | 1.07 |
|  | 15 | 1.01 | 0.98 | 1.03 | 1.00 | 1.00 | 1.01 | 1.00 | 0.99 | 1.00 | 0-15 | **1.32** | **1.02** | **1.70** | **1.12** | **1.04** | **1.20** | 0.99 | 0.91 | 1.07 |
|  | 16 | 1.00 | 0.97 | 1.02 | 1.00 | 0.99 | 1.01 | 0.99 | 0.99 | 1.00 | 0-16 | **1.32** | **1.02** | **1.71** | **1.12** | **1.04** | **1.20** | 0.98 | 0.90 | 1.07 |
|  | 17 | 0.99 | 0.97 | 1.02 | 1.00 | 0.99 | 1.01 | 0.99 | 0.99 | 1.00 | 0-17 | **1.31** | **1.00** | **1.71** | **1.12** | **1.04** | **1.20** | 0.98 | 0.90 | 1.06 |
|  | 18 | 0.99 | 0.97 | 1.02 | 1.00 | 0.99 | 1.01 | 0.99 | 0.99 | 1.00 | 0-18 | 1.30 | 0.98 | 1.71 | **1.12** | **1.04** | **1.21** | 0.97 | 0.89 | 1.06 |
|  | 19 | 0.99 | 0.96 | 1.02 | 1.00 | 0.99 | 1.01 | 1.00 | 0.99 | 1.00 | 0-19 | 1.28 | 0.97 | 1.70 | **1.12** | **1.03** | **1.21** | 0.97 | 0.88 | 1.06 |
|  | 20 | 0.99 | 0.96 | 1.02 | 1.00 | 0.99 | 1.01 | 1.00 | 0.99 | 1.01 | 0-20 | 1.27 | 0.95 | 1.70 | **1.11** | **1.03** | **1.21** | 0.96 | 0.88 | 1.06 |
|  | 21 | 1.00 | 0.95 | 1.06 | 1.00 | 0.99 | 1.02 | 1.00 | 0.98 | 1.02 | 0-21 | 1.27 | 0.94 | 1.73 | **1.12** | **1.02** | **1.22** | 0.96 | 0.87 | 1.06 |
| DHI | 0 | 1.09 | 0.90 | 1.32 | 1.04 | 0.99 | 1.09 | 0.98 | 0.93 | 1.04 | 0-0 | 1.09 | 0.90 | 1.32 | 1.04 | 0.99 | 1.09 | 0.98 | 0.93 | 1.04 |
|  | 1 | 1.04 | 0.93 | 1.17 | 1.02 | 0.99 | 1.06 | 0.99 | 0.95 | 1.02 | 0-1 | 1.14 | 0.84 | 1.53 | 1.06 | 0.98 | 1.15 | 0.97 | 0.88 | 1.06 |
|  | 2 | 1.00 | 0.92 | 1.10 | 1.02 | 0.99 | 1.04 | 0.99 | 0.96 | 1.02 | 0-2 | 1.14 | 0.79 | 1.64 | 1.08 | 0.97 | 1.19 | 0.96 | 0.86 | 1.07 |
|  | 3 | 0.98 | 0.90 | 1.07 | 1.01 | 0.99 | 1.04 | 1.00 | 0.97 | 1.02 | 0-3 | 1.12 | 0.74 | 1.69 | 1.09 | 0.97 | 1.22 | 0.95 | 0.84 | 1.08 |
|  | 4 | 0.97 | 0.88 | 1.06 | 1.02 | 0.99 | 1.04 | 1.00 | 0.97 | 1.03 | 0-4 | 1.08 | 0.68 | 1.70 | 1.11 | 0.98 | 1.25 | 0.95 | 0.83 | 1.10 |
|  | 5 | 0.96 | 0.88 | 1.05 | 1.02 | 1.00 | 1.05 | 1.00 | 0.98 | 1.03 | 0-5 | 1.04 | 0.63 | 1.71 | 1.13 | 0.98 | 1.29 | 0.96 | 0.82 | 1.12 |
|  | 6 | 0.96 | 0.89 | 1.04 | **1.03** | **1.00** | **1.05** | 1.01 | 0.98 | 1.03 | 0-6 | 1.00 | 0.58 | 1.71 | 1.16 | 1.00 | 1.35 | 0.96 | 0.81 | 1.14 |
|  | 7 | 0.97 | 0.90 | 1.05 | **1.03** | **1.01** | **1.06** | 1.01 | 0.98 | 1.03 | 0-7 | 0.97 | 0.54 | 1.73 | **1.20** | **1.02** | **1.40** | 0.97 | 0.81 | 1.16 |
|  | 8 | 0.98 | 0.91 | 1.06 | **1.04** | **1.01** | **1.06** | 1.01 | 0.99 | 1.04 | 0-8 | 0.95 | 0.51 | 1.77 | **1.24** | **1.04** | **1.47** | 0.98 | 0.80 | 1.19 |
|  | 9 | 0.99 | 0.92 | 1.08 | **1.04** | **1.02** | **1.06** | 1.01 | 0.98 | 1.04 | 0-9 | 0.94 | 0.48 | 1.84 | **1.29** | **1.07** | **1.55** | 0.99 | 0.80 | 1.22 |
|  | 10 | 1.01 | 0.93 | 1.10 | **1.04** | **1.02** | **1.07** | 1.01 | 0.98 | 1.04 | 0-10 | 0.95 | 0.47 | 1.94 | **1.34** | **1.10** | **1.63** | 1.00 | 0.80 | 1.25 |
|  | 11 | 1.03 | 0.94 | 1.11 | **1.04** | **1.02** | **1.07** | 1.01 | 0.98 | 1.04 | 0-11 | 0.97 | 0.46 | 2.08 | **1.40** | **1.13** | **1.72** | 1.01 | 0.79 | 1.28 |
|  | 12 | 1.04 | 0.96 | 1.13 | **1.04** | **1.01** | **1.06** | 1.01 | 0.98 | 1.04 | 0-12 | 1.01 | 0.45 | 2.27 | **1.45** | **1.16** | **1.81** | 1.02 | 0.79 | 1.31 |
|  | 13 | 1.05 | 0.97 | 1.14 | **1.03** | **1.01** | **1.06** | 1.01 | 0.98 | 1.03 | 0-13 | 1.07 | 0.46 | 2.49 | **1.50** | **1.18** | **1.89** | 1.02 | 0.78 | 1.33 |
|  | 14 | 1.06 | 0.98 | 1.15 | **1.03** | **1.00** | **1.05** | 1.00 | 0.98 | 1.03 | 0-14 | 1.13 | 0.47 | 2.75 | **1.53** | **1.20** | **1.95** | 1.03 | 0.78 | 1.35 |
|  | 15 | 1.06 | 0.98 | 1.16 | 1.02 | 0.99 | 1.04 | 1.00 | 0.98 | 1.03 | 0-15 | 1.21 | 0.48 | 3.05 | **1.56** | **1.21** | **2.01** | 1.03 | 0.77 | 1.37 |
|  | 16 | 1.06 | 0.97 | 1.16 | 1.01 | 0.98 | 1.03 | 1.00 | 0.97 | 1.03 | 0-16 | 1.28 | 0.49 | 3.36 | **1.57** | **1.21** | **2.04** | 1.03 | 0.76 | 1.39 |
|  | 17 | 1.05 | 0.96 | 1.15 | 1.00 | 0.98 | 1.03 | 1.00 | 0.97 | 1.03 | 0-17 | 1.34 | 0.49 | 3.66 | **1.57** | **1.20** | **2.07** | 1.03 | 0.75 | 1.40 |
|  | 18 | 1.03 | 0.94 | 1.13 | 1.00 | 0.97 | 1.02 | 1.00 | 0.97 | 1.03 | 0-18 | 1.38 | 0.49 | 3.92 | **1.57** | **1.18** | **2.08** | 1.02 | 0.74 | 1.41 |
|  | 19 | 1.00 | 0.91 | 1.09 | 1.00 | 0.97 | 1.02 | 1.00 | 0.97 | 1.03 | 0-19 | 1.38 | 0.47 | 4.07 | **1.56** | **1.16** | **2.09** | 1.02 | 0.73 | 1.43 |
|  | 20 | 0.96 | 0.86 | 1.07 | 1.00 | 0.97 | 1.03 | 1.00 | 0.97 | 1.03 | 0-20 | 1.32 | 0.43 | 4.06 | **1.56** | **1.15** | **2.11** | 1.02 | 0.72 | 1.44 |
|  | 21 | 0.91 | 0.77 | 1.08 | 1.01 | 0.96 | 1.06 | 1.00 | 0.95 | 1.06 | 0-21 | 1.21 | 0.38 | 3.89 | **1.57** | **1.14** | **2.16** | 1.02 | 0.71 | 1.46 |
| DNI | 0 | 1.01 | 0.97 | 1.06 | **1.02** | **1.00** | **1.03** | 1.01 | 0.99 | 1.02 | 0-0 | 1.01 | 0.97 | 1.06 | **1.02** | **1.00** | **1.03** | 1.01 | 0.99 | 1.02 |
|  | 1 | 1.01 | 0.98 | 1.04 | **1.01** | **1.00** | **1.02** | 1.01 | 1.00 | 1.01 | 0-1 | 1.02 | 0.95 | 1.10 | **1.02** | **1.01** | **1.05** | 1.01 | 0.99 | 1.04 |
|  | 2 | 1.01 | 0.99 | 1.03 | **1.01** | **1.00** | **1.01** | 1.00 | 1.00 | 1.01 | 0-2 | 1.03 | 0.95 | 1.12 | **1.03** | **1.01** | **1.06** | 1.02 | 0.99 | 1.04 |
|  | 3 | 1.01 | 0.99 | 1.03 | 1.00 | 1.00 | 1.01 | 1.00 | 1.00 | 1.01 | 0-3 | 1.04 | 0.95 | 1.14 | **1.04** | **1.01** | **1.06** | 1.02 | 0.99 | 1.05 |
|  | 4 | 1.01 | 1.00 | 1.03 | 1.00 | 1.00 | 1.01 | 1.00 | 1.00 | 1.01 | 0-4 | 1.06 | 0.96 | 1.16 | **1.04** | **1.01** | **1.07** | 1.02 | 0.99 | 1.05 |
|  | 5 | 1.01 | 1.00 | 1.03 | 1.00 | 1.00 | 1.01 | 1.00 | 0.99 | 1.00 | 0-5 | 1.07 | 0.97 | 1.18 | **1.04** | **1.01** | **1.07** | 1.02 | 0.98 | 1.05 |
|  | 6 | **1.02** | **1.00** | **1.03** | 1.00 | 1.00 | 1.01 | 1.00 | 1.00 | 1.00 | 0-6 | 1.09 | 0.98 | 1.21 | **1.04** | **1.01** | **1.07** | 1.02 | 0.98 | 1.05 |
|  | 7 | **1.02** | **1.00** | **1.03** | 1.00 | 1.00 | 1.01 | 1.00 | 1.00 | 1.00 | 0-7 | 1.11 | 0.99 | 1.23 | **1.04** | **1.01** | **1.08** | 1.02 | 0.98 | 1.05 |
|  | 8 | **1.02** | **1.00** | **1.03** | 1.00 | 1.00 | 1.01 | 1.00 | 1.00 | 1.00 | 0-8 | **1.12** | **1.00** | **1.26** | **1.05** | **1.01** | **1.08** | 1.01 | 0.98 | 1.05 |
|  | 9 | **1.02** | **1.00** | **1.03** | 1.00 | 1.00 | 1.01 | 1.00 | 1.00 | 1.00 | 0-9 | **1.14** | **1.01** | **1.29** | **1.05** | **1.01** | **1.08** | 1.01 | 0.97 | 1.05 |
|  | 10 | **1.02** | **1.00** | **1.03** | 1.00 | 1.00 | 1.01 | 1.00 | 0.99 | 1.00 | 0-10 | **1.16** | **1.02** | **1.32** | **1.05** | **1.01** | **1.09** | 1.01 | 0.97 | 1.05 |
|  | 11 | **1.01** | **1.00** | **1.03** | 1.00 | 1.00 | 1.01 | 1.00 | 0.99 | 1.00 | 0-11 | **1.18** | **1.03** | **1.34** | **1.05** | **1.01** | **1.09** | 1.01 | 0.97 | 1.05 |
|  | 12 | 1.01 | 1.00 | 1.03 | 1.00 | 1.00 | 1.01 | 1.00 | 0.99 | 1.00 | 0-12 | **1.19** | **1.04** | **1.37** | **1.05** | **1.01** | **1.09** | 1.01 | 0.96 | 1.05 |
|  | 13 | 1.01 | 1.00 | 1.02 | 1.00 | 1.00 | 1.01 | 1.00 | 0.99 | 1.00 | 0-13 | **1.20** | **1.04** | **1.38** | **1.06** | **1.01** | **1.10** | 1.00 | 0.96 | 1.05 |
|  | 14 | 1.00 | 0.99 | 1.02 | 1.00 | 1.00 | 1.00 | 1.00 | 0.99 | 1.00 | 0-14 | **1.20** | **1.04** | **1.40** | **1.06** | **1.01** | **1.10** | 1.00 | 0.95 | 1.05 |
|  | 15 | 1.00 | 0.99 | 1.02 | 1.00 | 1.00 | 1.00 | 1.00 | 0.99 | 1.00 | 0-15 | **1.21** | **1.03** | **1.40** | **1.06** | **1.01** | **1.10** | 1.00 | 0.95 | 1.05 |
|  | 16 | 1.00 | 0.98 | 1.01 | 1.00 | 1.00 | 1.00 | 1.00 | 0.99 | 1.00 | 0-16 | **1.20** | **1.03** | **1.41** | **1.06** | **1.01** | **1.10** | 0.99 | 0.94 | 1.04 |
|  | 17 | 1.00 | 0.98 | 1.01 | 1.00 | 1.00 | 1.00 | 1.00 | 0.99 | 1.00 | 0-17 | **1.20** | **1.02** | **1.41** | **1.06** | **1.01** | **1.10** | 0.99 | 0.94 | 1.04 |
|  | 18 | 1.00 | 0.98 | 1.01 | 1.00 | 1.00 | 1.00 | 1.00 | 0.99 | 1.00 | 0-18 | **1.19** | **1.01** | **1.41** | **1.06** | **1.01** | **1.10** | 0.98 | 0.93 | 1.04 |
|  | 19 | 1.00 | 0.98 | 1.01 | 1.00 | 1.00 | 1.00 | 1.00 | 0.99 | 1.00 | 0-19 | 1.19 | 1.00 | 1.41 | **1.05** | **1.01** | **1.11** | 0.98 | 0.93 | 1.04 |
|  | 20 | 1.00 | 0.98 | 1.02 | 1.00 | 1.00 | 1.01 | 1.00 | 0.99 | 1.01 | 0-20 | 1.19 | 0.99 | 1.41 | **1.06** | **1.00** | **1.11** | 0.98 | 0.92 | 1.04 |
|  | 21 | 1.01 | 0.97 | 1.04 | 1.00 | 0.99 | 1.01 | 1.00 | 0.99 | 1.01 | 0-21 | 1.19 | 0.99 | 1.44 | **1.06** | **1.00** | **1.12** | 0.98 | 0.92 | 1.04 |

Note. Statistically significant (*p* < 0.05) were labeled in bold font; RR: relative risk; UCI: upper confidence interval; LCI: lower confidence interval; DHI: diffuse horizontal irradiance; DNI: direct normal irradiance; GHI: global horizontal irradiance.

**Supplementary Table 4.** Lag-specific relative risks and cumulative risks in outpatient visits for meibomian gland dysfunction associated with GHI, DHI, and DNI in cold and warm seasons.

|  | Single-day lag RR (95%CI) | | | | | | | Cumulative -day lag RR (95%CI) | | | | | | |
| --- | --- | --- | --- | --- | --- | --- | --- | --- | --- | --- | --- | --- | --- | --- |
|  |  | Cold season | | | Warm season | | |  | Cold season | | | Warm season | | |
|  | Lag days | RR | LCI | UCI | RR | LCI | UCI | Lag days | RR | LCI | UCI | RR | LCI | UCI |
| GHI | 0 | 1.00 | 0.97 | 1.04 | 0.99 | 0.95 | 1.04 | 0-0 | 1.00 | 0.97 | 1.04 | 0.99 | 0.95 | 1.04 |
|  | 1 | 1.00 | 0.98 | 1.02 | 1.00 | 0.97 | 1.03 | 0-1 | 1.00 | 0.96 | 1.05 | 0.99 | 0.92 | 1.06 |
|  | 2 | 0.99 | 0.98 | 1.01 | 1.00 | 0.98 | 1.02 | 0-2 | 1.00 | 0.94 | 1.05 | 0.99 | 0.90 | 1.08 |
|  | 3 | 0.99 | 0.98 | 1.01 | 1.01 | 0.99 | 1.03 | 0-3 | 0.99 | 0.93 | 1.05 | 0.99 | 0.90 | 1.10 |
|  | 4 | 0.99 | 0.98 | 1.01 | 1.01 | 0.99 | 1.03 | 0-4 | 0.98 | 0.92 | 1.05 | 1.00 | 0.90 | 1.11 |
|  | 5 | 1.00 | 0.98 | 1.01 | 1.01 | 0.99 | 1.03 | 0-5 | 0.98 | 0.92 | 1.05 | 1.01 | 0.90 | 1.13 |
|  | 6 | 1.00 | 0.99 | 1.01 | 1.01 | 0.99 | 1.03 | 0-6 | 0.98 | 0.91 | 1.05 | 1.01 | 0.89 | 1.15 |
|  | 7 | 1.00 | 0.99 | 1.01 | 1.01 | 0.99 | 1.02 | 0-7 | 0.98 | 0.91 | 1.05 | 1.02 | 0.89 | 1.16 |
|  | 8 | 1.00 | 0.99 | 1.01 | 1.00 | 0.99 | 1.02 | 0-8 | 0.98 | 0.90 | 1.06 | 1.02 | 0.88 | 1.18 |
|  | 9 | 1.00 | 0.99 | 1.01 | 1.00 | 0.98 | 1.02 | 0-9 | 0.98 | 0.90 | 1.06 | 1.02 | 0.87 | 1.19 |
|  | 10 | 1.00 | 0.99 | 1.01 | 1.00 | 0.98 | 1.02 | 0-10 | 0.98 | 0.90 | 1.07 | 1.02 | 0.86 | 1.20 |
|  | 11 | 1.00 | 0.99 | 1.01 | 1.00 | 0.98 | 1.02 | 0-11 | 0.98 | 0.89 | 1.07 | 1.01 | 0.85 | 1.21 |
|  | 12 | 1.00 | 0.99 | 1.01 | 0.99 | 0.98 | 1.01 | 0-12 | 0.98 | 0.88 | 1.08 | 1.01 | 0.83 | 1.22 |
|  | 13 | 0.99 | 0.99 | 1.00 | 0.99 | 0.97 | 1.01 | 0-13 | 0.97 | 0.87 | 1.07 | 1.00 | 0.81 | 1.23 |
|  | 14 | 0.99 | 0.98 | 1.00 | 0.99 | 0.97 | 1.01 | 0-14 | 0.96 | 0.86 | 1.07 | 0.99 | 0.80 | 1.23 |
|  | 15 | 0.99 | 0.98 | 1.00 | 0.99 | 0.97 | 1.01 | 0-15 | 0.94 | 0.85 | 1.05 | 0.98 | 0.78 | 1.23 |
|  | 16 | 0.98 | 0.97 | 0.99 | 0.99 | 0.97 | 1.01 | 0-16 | 0.93 | 0.83 | 1.04 | 0.96 | 0.76 | 1.22 |
|  | 17 | 0.98 | 0.97 | 0.99 | 0.99 | 0.97 | 1.01 | 0-17 | 0.91 | 0.81 | 1.02 | 0.95 | 0.74 | 1.22 |
|  | 18 | 0.98 | 0.97 | 0.99 | 0.99 | 0.97 | 1.01 | 0-18 | 0.89 | 0.79 | 1.01 | 0.94 | 0.72 | 1.21 |
|  | 19 | 0.98 | 0.97 | 0.99 | 0.99 | 0.97 | 1.01 | 0-19 | 0.88 | 0.77 | 1.00 | 0.92 | 0.70 | 1.21 |
|  | 20 | 0.99 | 0.98 | 1.00 | 0.99 | 0.96 | 1.01 | 0-20 | 0.87 | 0.76 | 0.99 | 0.91 | 0.68 | 1.21 |
|  | 21 | 1.00 | 0.98 | 1.03 | 0.99 | 0.95 | 1.03 | 0-21 | 0.87 | 0.75 | 1.00 | 0.90 | 0.67 | 1.21 |
| DHI | 0 | **1.11** | **1.02** | **1.22** | 0.95 | 0.84 | 1.07 | 0-0 | **1.11** | **1.02** | **1.22** | 0.95 | 0.84 | 1.07 |
|  | 1 | **1.08** | **1.01** | **1.14** | 0.96 | 0.89 | 1.03 | 0-1 | **1.20** | **1.04** | **1.39** | 0.91 | 0.75 | 1.10 |
|  | 2 | 1.05 | 1.00 | 1.11 | 0.97 | 0.92 | 1.03 | 0-2 | **1.26** | **1.05** | **1.52** | 0.88 | 0.70 | 1.11 |
|  | 3 | 1.04 | 0.98 | 1.10 | 0.99 | 0.93 | 1.05 | 0-3 | **1.31** | **1.05** | **1.63** | 0.87 | 0.67 | 1.13 |
|  | 4 | 1.03 | 0.97 | 1.09 | 1.00 | 0.94 | 1.06 | 0-4 | **1.34** | **1.03** | **1.74** | 0.87 | 0.66 | 1.16 |
|  | 5 | 1.02 | 0.97 | 1.08 | 1.01 | 0.95 | 1.07 | 0-5 | **1.37** | **1.02** | **1.84** | 0.88 | 0.64 | 1.20 |
|  | 6 | 1.02 | 0.97 | 1.07 | 1.02 | 0.97 | 1.07 | 0-6 | **1.40** | **1.01** | **1.95** | 0.89 | 0.64 | 1.26 |
|  | 7 | 1.02 | 0.98 | 1.07 | 1.02 | 0.97 | 1.08 | 0-7 | 1.43 | 1.00 | 2.06 | 0.92 | 0.63 | 1.32 |
|  | 8 | 1.02 | 0.98 | 1.07 | 1.03 | 0.97 | 1.08 | 0-8 | 1.47 | 0.99 | 2.17 | 0.94 | 0.63 | 1.40 |
|  | 9 | 1.03 | 0.98 | 1.08 | 1.03 | 0.97 | 1.08 | 0-9 | 1.50 | 0.98 | 2.29 | 0.97 | 0.63 | 1.48 |
|  | 10 | 1.02 | 0.98 | 1.08 | 1.03 | 0.97 | 1.08 | 0-10 | 1.54 | 0.98 | 2.42 | 0.99 | 0.63 | 1.56 |
|  | 11 | 1.02 | 0.97 | 1.08 | 1.02 | 0.96 | 1.08 | 0-11 | 1.57 | 0.97 | 2.55 | 1.01 | 0.62 | 1.64 |
|  | 12 | 1.02 | 0.97 | 1.07 | 1.01 | 0.96 | 1.07 | 0-12 | 1.60 | 0.96 | 2.69 | 1.02 | 0.61 | 1.71 |
|  | 13 | 1.01 | 0.97 | 1.06 | 1.00 | 0.95 | 1.06 | 0-13 | 1.63 | 0.94 | 2.81 | 1.03 | 0.60 | 1.76 |
|  | 14 | 1.01 | 0.96 | 1.06 | 0.99 | 0.94 | 1.05 | 0-14 | 1.64 | 0.92 | 2.93 | 1.02 | 0.58 | 1.80 |
|  | 15 | 1.00 | 0.95 | 1.05 | 0.99 | 0.93 | 1.04 | 0-15 | 1.64 | 0.89 | 3.02 | 1.00 | 0.56 | 1.81 |
|  | 16 | 0.99 | 0.94 | 1.04 | 0.98 | 0.92 | 1.03 | 0-16 | 1.62 | 0.85 | 3.09 | 0.98 | 0.53 | 1.81 |
|  | 17 | 0.98 | 0.93 | 1.04 | 0.97 | 0.91 | 1.03 | 0-17 | 1.60 | 0.81 | 3.14 | 0.95 | 0.50 | 1.79 |
|  | 18 | 0.98 | 0.93 | 1.03 | 0.96 | 0.91 | 1.03 | 0-18 | 1.56 | 0.77 | 3.18 | 0.92 | 0.47 | 1.77 |
|  | 19 | 0.98 | 0.93 | 1.03 | 0.96 | 0.91 | 1.03 | 0-19 | 1.53 | 0.73 | 3.20 | 0.88 | 0.44 | 1.76 |
|  | 20 | 0.98 | 0.93 | 1.04 | 0.97 | 0.90 | 1.04 | 0-20 | 1.50 | 0.69 | 3.23 | 0.86 | 0.42 | 1.76 |
|  | 21 | 0.99 | 0.91 | 1.08 | 0.98 | 0.87 | 1.10 | 0-21 | 1.48 | 0.67 | 3.28 | 0.84 | 0.39 | 1.80 |
| DNI | 0 | 1.00 | 0.99 | 1.02 | 1.00 | 0.97 | 1.04 | 0-0 | 1.00 | 0.99 | 1.02 | 1.00 | 0.97 | 1.04 |
|  | 1 | 1.00 | 0.99 | 1.01 | 1.00 | 0.98 | 1.03 | 0-1 | 1.00 | 0.98 | 1.02 | 1.00 | 0.95 | 1.06 |
|  | 2 | 1.00 | 0.99 | 1.00 | 1.01 | 0.99 | 1.02 | 0-2 | 1.00 | 0.97 | 1.02 | 1.01 | 0.94 | 1.08 |
|  | 3 | 1.00 | 0.99 | 1.00 | 1.01 | 0.99 | 1.02 | 0-3 | 0.99 | 0.96 | 1.02 | 1.02 | 0.94 | 1.10 |
|  | 4 | 1.00 | 0.99 | 1.00 | 1.01 | 0.99 | 1.02 | 0-4 | 0.99 | 0.96 | 1.02 | 1.02 | 0.94 | 1.11 |
|  | 5 | 1.00 | 0.99 | 1.00 | 1.01 | 0.99 | 1.02 | 0-5 | 0.99 | 0.95 | 1.02 | 1.03 | 0.93 | 1.13 |
|  | 6 | 1.00 | 0.99 | 1.00 | 1.00 | 0.99 | 1.02 | 0-6 | 0.98 | 0.95 | 1.02 | 1.03 | 0.93 | 1.14 |
|  | 7 | 1.00 | 0.99 | 1.00 | 1.00 | 0.99 | 1.02 | 0-7 | 0.98 | 0.95 | 1.02 | 1.03 | 0.92 | 1.15 |
|  | 8 | 1.00 | 0.99 | 1.00 | 1.00 | 0.99 | 1.01 | 0-8 | 0.98 | 0.94 | 1.02 | 1.03 | 0.92 | 1.16 |
|  | 9 | 1.00 | 0.99 | 1.00 | 1.00 | 0.98 | 1.01 | 0-9 | 0.98 | 0.94 | 1.02 | 1.03 | 0.91 | 1.17 |
|  | 10 | 1.00 | 0.99 | 1.00 | 1.00 | 0.98 | 1.01 | 0-10 | 0.98 | 0.94 | 1.02 | 1.02 | 0.89 | 1.17 |
|  | 11 | 1.00 | 0.99 | 1.00 | 0.99 | 0.98 | 1.01 | 0-11 | 0.98 | 0.93 | 1.02 | 1.02 | 0.88 | 1.18 |
|  | 12 | 1.00 | 0.99 | 1.00 | 0.99 | 0.98 | 1.01 | 0-12 | 0.97 | 0.93 | 1.02 | 1.01 | 0.86 | 1.18 |
|  | 13 | 1.00 | 0.99 | 1.00 | 0.99 | 0.98 | 1.01 | 0-13 | 0.97 | 0.92 | 1.02 | 1.00 | 0.85 | 1.18 |
|  | 14 | 0.99 | 0.99 | 1.00 | 0.99 | 0.98 | 1.00 | 0-14 | 0.96 | 0.91 | 1.02 | 0.99 | 0.83 | 1.18 |
|  | 15 | 0.99 | 0.99 | 1.00 | 0.99 | 0.97 | 1.00 | 0-15 | 0.96 | 0.90 | 1.01 | 0.98 | 0.81 | 1.18 |
|  | 16 | 0.99 | 0.99 | 1.00 | 0.99 | 0.97 | 1.00 | 0-16 | 0.95 | 0.89 | 1.00 | 0.97 | 0.80 | 1.18 |
|  | 17 | 0.99 | 0.99 | 1.00 | 0.99 | 0.97 | 1.00 | 0-17 | **0.94** | **0.88** | **1.00** | 0.96 | 0.78 | 1.17 |
|  | 18 | 0.99 | 0.99 | 1.00 | 0.99 | 0.97 | 1.00 | 0-18 | **0.93** | **0.87** | **0.99** | 0.95 | 0.76 | 1.17 |
|  | 19 | 0.99 | 0.99 | 1.00 | 0.99 | 0.97 | 1.01 | 0-19 | **0.92** | **0.86** | **0.98** | 0.94 | 0.75 | 1.17 |
|  | 20 | 1.00 | 0.99 | 1.00 | 0.99 | 0.97 | 1.01 | 0-20 | **0.92** | **0.86** | **0.98** | 0.93 | 0.73 | 1.17 |
|  | 21 | 1.00 | 0.99 | 1.01 | 0.99 | 0.96 | 1.02 | 0-21 | **0.92** | **0.85** | **0.98** | 0.92 | 0.72 | 1.17 |

Note. Statistically significant (*p* < 0.05) were labeled in bold font; RR: relative risk; UCI: upper confidence interval; LCI: lower confidence interval; DHI: diffuse horizontal irradiance; DNI: direct normal irradiance; GHI: global horizontal irradiance.

**Supplementary Table 5.** The sensitivity analysis of changing df for time (6–8) demonstrated the impacts of solar radiation on outpatient visits for meibomian gland dysfunction.

|  | Single-day lag RR (95%CI) | | | | | | | | | | Cumulative-day lag RR (95%CI) | | | | | | | | | |
| --- | --- | --- | --- | --- | --- | --- | --- | --- | --- | --- | --- | --- | --- | --- | --- | --- | --- | --- | --- | --- |
|  |  | Time df = 6 | | | Time df = 7 | | | Time df = 8 | | |  | Time df = 6 | | | Time df = 7 | | | Time df = 8 | | |
|  | Lag days | RR | LCI | UCI | RR | LCI | UCI | RR | LCI | UCI | Lag days | RR | LCI | UCI | RR | LCI | UCI | RR | LCI | UCI |
| GHI | 0 | 1.01 | 0.99 | 1.03 | 1.02 | 1.00 | 1.04 | 1.01 | 1.00 | 1.03 | 0-0 | 1.01 | 0.99 | 1.03 | 1.02 | 1.00 | 1.04 | 1.01 | 1.00 | 1.03 |
|  | 1 | 1.01 | 1.00 | 1.02 | 1.01 | 1.00 | 1.02 | 1.01 | 1.00 | 1.02 | 0-1 | 1.02 | 0.99 | 1.05 | 1.03 | 1.00 | 1.06 | 1.03 | 1.00 | 1.06 |
|  | 2 | 1.01 | 1.00 | 1.01 | 1.01 | 1.00 | 1.02 | 1.01 | 1.00 | 1.01 | 0-2 | 1.03 | 0.99 | 1.07 | 1.04 | 1.00 | 1.07 | 1.03 | 1.00 | 1.07 |
|  | 3 | 1.01 | 1.00 | 1.01 | 1.01 | 1.00 | 1.01 | 1.00 | 1.00 | 1.01 | 0-3 | 1.03 | 0.99 | 1.08 | 1.04 | 1.00 | 1.08 | 1.04 | 1.00 | 1.08 |
|  | 4 | 1.00 | 1.00 | 1.01 | 1.00 | 1.00 | 1.01 | 1.00 | 1.00 | 1.01 | 0-4 | 1.04 | 1.00 | 1.08 | 1.05 | 1.01 | 1.09 | 1.04 | 1.00 | 1.08 |
|  | 5 | 1.00 | 1.00 | 1.01 | 1.00 | 1.00 | 1.01 | 1.00 | 1.00 | 1.01 | 0-5 | 1.04 | 1.00 | 1.09 | 1.05 | 1.01 | 1.09 | 1.04 | 1.00 | 1.08 |
|  | 6 | 1.00 | 1.00 | 1.01 | 1.00 | 1.00 | 1.01 | 1.00 | 1.00 | 1.01 | 0-6 | 1.05 | 1.00 | 1.09 | 1.05 | 1.01 | 1.10 | 1.04 | 1.00 | 1.09 |
|  | 7 | 1.00 | 1.00 | 1.01 | 1.00 | 1.00 | 1.01 | 1.00 | 1.00 | 1.01 | 0-7 | 1.05 | 1.00 | 1.10 | 1.05 | 1.01 | 1.10 | 1.04 | 1.00 | 1.09 |
|  | 8 | 1.00 | 1.00 | 1.01 | 1.00 | 1.00 | 1.01 | 1.00 | 1.00 | 1.01 | 0-8 | 1.05 | 1.00 | 1.10 | 1.06 | 1.01 | 1.11 | 1.05 | 1.00 | 1.09 |
|  | 9 | 1.00 | 1.00 | 1.01 | 1.00 | 1.00 | 1.01 | 1.00 | 1.00 | 1.01 | 0-9 | 1.05 | 1.00 | 1.10 | 1.06 | 1.01 | 1.11 | 1.05 | 1.00 | 1.10 |
|  | 10 | 1.00 | 1.00 | 1.01 | 1.00 | 1.00 | 1.01 | 1.00 | 1.00 | 1.01 | 0-10 | 1.05 | 1.00 | 1.11 | 1.06 | 1.01 | 1.11 | 1.05 | 1.00 | 1.10 |
|  | 11 | 1.00 | 0.99 | 1.01 | 1.00 | 1.00 | 1.01 | 1.00 | 0.99 | 1.01 | 0-11 | 1.05 | 1.00 | 1.11 | 1.06 | 1.01 | 1.12 | 1.05 | 0.99 | 1.10 |
|  | 12 | 1.00 | 0.99 | 1.01 | 1.00 | 1.00 | 1.01 | 1.00 | 0.99 | 1.01 | 0-12 | 1.05 | 0.99 | 1.11 | 1.06 | 1.01 | 1.12 | 1.05 | 0.99 | 1.11 |
|  | 13 | 1.00 | 0.99 | 1.00 | 1.00 | 1.00 | 1.01 | 1.00 | 0.99 | 1.00 | 0-13 | 1.05 | 0.99 | 1.11 | 1.06 | 1.00 | 1.13 | 1.05 | 0.99 | 1.11 |
|  | 14 | 1.00 | 0.99 | 1.00 | 1.00 | 0.99 | 1.00 | 1.00 | 0.99 | 1.00 | 0-14 | 1.05 | 0.99 | 1.11 | 1.06 | 1.00 | 1.13 | 1.04 | 0.98 | 1.11 |
|  | 15 | 1.00 | 0.99 | 1.00 | 1.00 | 0.99 | 1.00 | 1.00 | 0.99 | 1.00 | 0-15 | 1.05 | 0.98 | 1.11 | 1.06 | 1.00 | 1.13 | 1.04 | 0.98 | 1.11 |
|  | 16 | 1.00 | 0.99 | 1.00 | 1.00 | 0.99 | 1.00 | 1.00 | 0.99 | 1.00 | 0-16 | 1.04 | 0.98 | 1.11 | 1.06 | 0.99 | 1.13 | 1.04 | 0.97 | 1.11 |
|  | 17 | 1.00 | 0.99 | 1.00 | 1.00 | 0.99 | 1.00 | 1.00 | 0.99 | 1.00 | 0-17 | 1.04 | 0.97 | 1.11 | 1.06 | 0.99 | 1.13 | 1.03 | 0.97 | 1.10 |
|  | 18 | 1.00 | 0.99 | 1.00 | 1.00 | 0.99 | 1.00 | 0.99 | 0.99 | 1.00 | 0-18 | 1.03 | 0.97 | 1.11 | 1.05 | 0.98 | 1.13 | 1.03 | 0.96 | 1.10 |
|  | 19 | 1.00 | 0.99 | 1.00 | 1.00 | 0.99 | 1.00 | 0.99 | 0.99 | 1.00 | 0-19 | 1.03 | 0.96 | 1.11 | 1.05 | 0.98 | 1.13 | 1.02 | 0.95 | 1.10 |
|  | 20 | 1.00 | 0.99 | 1.01 | 1.00 | 0.99 | 1.01 | 1.00 | 0.99 | 1.00 | 0-20 | 1.03 | 0.96 | 1.11 | 1.05 | 0.97 | 1.13 | 1.02 | 0.95 | 1.09 |
|  | 21 | 1.00 | 0.99 | 1.02 | 1.00 | 0.99 | 1.01 | 1.00 | 0.98 | 1.01 | 0-21 | 1.03 | 0.95 | 1.11 | 1.05 | 0.97 | 1.13 | 1.01 | 0.94 | 1.10 |
| DHI | 0 | 1.03 | 0.98 | 1.08 | 1.02 | 0.98 | 1.07 | 1.02 | 0.97 | 1.06 | 0-0 | 1.03 | 0.98 | 1.08 | 1.02 | 0.98 | 1.07 | 1.02 | 0.97 | 1.06 |
|  | 1 | 1.02 | 0.99 | 1.05 | 1.01 | 0.98 | 1.04 | 1.00 | 0.98 | 1.03 | 0-1 | 1.04 | 0.97 | 1.12 | 1.03 | 0.96 | 1.11 | 1.02 | 0.95 | 1.09 |
|  | 2 | 1.01 | 0.99 | 1.04 | 1.00 | 0.98 | 1.02 | 1.00 | 0.98 | 1.02 | 0-2 | 1.06 | 0.96 | 1.16 | 1.03 | 0.94 | 1.13 | 1.02 | 0.93 | 1.11 |
|  | 3 | 1.01 | 0.99 | 1.04 | 1.00 | 0.98 | 1.02 | 1.00 | 0.98 | 1.02 | 0-3 | 1.07 | 0.96 | 1.19 | 1.03 | 0.93 | 1.14 | 1.01 | 0.92 | 1.12 |
|  | 4 | 1.02 | 1.00 | 1.04 | 1.00 | 0.98 | 1.02 | 1.00 | 0.98 | 1.02 | 0-4 | 1.09 | 0.97 | 1.23 | 1.03 | 0.92 | 1.15 | 1.02 | 0.91 | 1.14 |
|  | 5 | 1.03 | 1.00 | 1.05 | 1.01 | 0.99 | 1.03 | 1.01 | 0.99 | 1.03 | 0-5 | 1.12 | 0.98 | 1.27 | 1.04 | 0.92 | 1.18 | 1.02 | 0.90 | 1.16 |
|  | 6 | 1.03 | 1.01 | 1.05 | 1.01 | 0.99 | 1.03 | 1.01 | 0.99 | 1.03 | 0-6 | 1.15 | 1.00 | 1.33 | 1.05 | 0.92 | 1.20 | 1.04 | 0.91 | 1.19 |
|  | 7 | 1.04 | 1.02 | 1.06 | 1.02 | 1.00 | 1.04 | 1.02 | 1.00 | 1.04 | 0-7 | 1.20 | 1.03 | 1.39 | 1.08 | 0.93 | 1.24 | 1.06 | 0.91 | 1.22 |
|  | 8 | 1.04 | 1.02 | 1.07 | 1.03 | 1.01 | 1.05 | 1.02 | 1.00 | 1.04 | 0-8 | 1.25 | 1.07 | 1.47 | 1.10 | 0.95 | 1.29 | 1.08 | 0.92 | 1.26 |
|  | 9 | 1.05 | 1.03 | 1.07 | 1.03 | 1.01 | 1.05 | 1.03 | 1.01 | 1.05 | 0-9 | 1.31 | 1.11 | 1.56 | 1.14 | 0.96 | 1.34 | 1.11 | 0.94 | 1.31 |
|  | 10 | 1.05 | 1.03 | 1.07 | 1.03 | 1.01 | 1.05 | 1.03 | 1.01 | 1.05 | 0-10 | 1.38 | 1.15 | 1.65 | 1.17 | 0.98 | 1.40 | 1.14 | 0.96 | 1.37 |
|  | 11 | 1.05 | 1.03 | 1.07 | 1.03 | 1.01 | 1.05 | 1.03 | 1.01 | 1.05 | 0-11 | 1.45 | 1.19 | 1.75 | 1.21 | 1.00 | 1.46 | 1.18 | 0.97 | 1.42 |
|  | 12 | 1.05 | 1.02 | 1.07 | 1.03 | 1.01 | 1.05 | 1.03 | 1.00 | 1.05 | 0-12 | 1.51 | 1.24 | 1.85 | 1.24 | 1.02 | 1.52 | 1.21 | 0.99 | 1.47 |
|  | 13 | 1.04 | 1.02 | 1.06 | 1.02 | 1.00 | 1.04 | 1.02 | 1.00 | 1.04 | 0-13 | 1.57 | 1.27 | 1.95 | 1.27 | 1.03 | 1.57 | 1.23 | 1.00 | 1.52 |
|  | 14 | 1.03 | 1.01 | 1.06 | 1.02 | 1.00 | 1.04 | 1.01 | 0.99 | 1.03 | 0-14 | 1.63 | 1.31 | 2.03 | 1.29 | 1.04 | 1.61 | 1.25 | 1.00 | 1.56 |
|  | 15 | 1.03 | 1.01 | 1.05 | 1.01 | 0.99 | 1.03 | 1.01 | 0.99 | 1.03 | 0-15 | 1.67 | 1.33 | 2.11 | 1.31 | 1.04 | 1.64 | 1.26 | 1.00 | 1.58 |
|  | 16 | 1.02 | 1.00 | 1.04 | 1.00 | 0.98 | 1.02 | 1.00 | 0.98 | 1.02 | 0-16 | 1.71 | 1.35 | 2.17 | 1.31 | 1.04 | 1.66 | 1.26 | 0.99 | 1.60 |
|  | 17 | 1.01 | 0.99 | 1.04 | 1.00 | 0.97 | 1.02 | 1.00 | 0.97 | 1.02 | 0-17 | 1.73 | 1.35 | 2.22 | 1.31 | 1.02 | 1.67 | 1.25 | 0.98 | 1.61 |
|  | 18 | 1.01 | 0.99 | 1.04 | 0.99 | 0.97 | 1.02 | 0.99 | 0.97 | 1.01 | 0-18 | 1.75 | 1.36 | 2.26 | 1.30 | 1.01 | 1.67 | 1.24 | 0.96 | 1.61 |
|  | 19 | 1.01 | 0.99 | 1.04 | 1.00 | 0.97 | 1.02 | 0.99 | 0.97 | 1.02 | 0-19 | 1.78 | 1.36 | 2.31 | 1.29 | 1.00 | 1.68 | 1.23 | 0.94 | 1.61 |
|  | 20 | 1.02 | 0.99 | 1.05 | 1.00 | 0.98 | 1.03 | 1.00 | 0.97 | 1.03 | 0-20 | 1.81 | 1.38 | 2.38 | 1.30 | 0.99 | 1.70 | 1.23 | 0.93 | 1.63 |
|  | 21 | 1.04 | 0.99 | 1.08 | 1.02 | 0.98 | 1.07 | 1.01 | 0.97 | 1.06 | 0-21 | 1.88 | 1.41 | 2.50 | 1.33 | 1.00 | 1.76 | 1.25 | 0.93 | 1.67 |
| DNI | 0 | 1.01 | 1.00 | 1.02 | 1.01 | 1.00 | 1.02 | 1.01 | 1.00 | 1.02 | 0-0 | 1.01 | 1.00 | 1.02 | 1.01 | 1.00 | 1.02 | 1.01 | 1.00 | 1.02 |
|  | 1 | 1.01 | 1.00 | 1.01 | 1.01 | 1.00 | 1.01 | 1.01 | 1.00 | 1.01 | 0-1 | 1.01 | 0.99 | 1.03 | 1.02 | 1.00 | 1.04 | 1.02 | 1.00 | 1.03 |
|  | 2 | 1.00 | 1.00 | 1.01 | 1.00 | 1.00 | 1.01 | 1.00 | 1.00 | 1.01 | 0-2 | 1.02 | 0.99 | 1.04 | 1.02 | 1.00 | 1.04 | 1.02 | 1.00 | 1.04 |
|  | 3 | 1.00 | 1.00 | 1.01 | 1.00 | 1.00 | 1.01 | 1.00 | 1.00 | 1.01 | 0-3 | 1.02 | 1.00 | 1.04 | 1.03 | 1.00 | 1.05 | 1.02 | 1.00 | 1.05 |
|  | 4 | 1.00 | 1.00 | 1.01 | 1.00 | 1.00 | 1.01 | 1.00 | 1.00 | 1.01 | 0-4 | 1.02 | 1.00 | 1.05 | 1.03 | 1.00 | 1.05 | 1.02 | 1.00 | 1.05 |
|  | 5 | 1.00 | 1.00 | 1.00 | 1.00 | 1.00 | 1.01 | 1.00 | 1.00 | 1.00 | 0-5 | 1.02 | 0.99 | 1.05 | 1.03 | 1.00 | 1.06 | 1.03 | 1.00 | 1.05 |
|  | 6 | 1.00 | 1.00 | 1.00 | 1.00 | 1.00 | 1.00 | 1.00 | 1.00 | 1.00 | 0-6 | 1.02 | 0.99 | 1.05 | 1.03 | 1.00 | 1.06 | 1.03 | 1.00 | 1.05 |
|  | 7 | 1.00 | 1.00 | 1.00 | 1.00 | 1.00 | 1.00 | 1.00 | 1.00 | 1.00 | 0-7 | 1.02 | 0.99 | 1.05 | 1.03 | 1.00 | 1.06 | 1.03 | 1.00 | 1.05 |
|  | 8 | 1.00 | 1.00 | 1.00 | 1.00 | 1.00 | 1.00 | 1.00 | 1.00 | 1.00 | 0-8 | 1.02 | 0.99 | 1.05 | 1.03 | 1.00 | 1.06 | 1.03 | 1.00 | 1.06 |
|  | 9 | 1.00 | 1.00 | 1.00 | 1.00 | 1.00 | 1.00 | 1.00 | 1.00 | 1.00 | 0-9 | 1.02 | 0.99 | 1.05 | 1.03 | 1.00 | 1.06 | 1.03 | 0.99 | 1.06 |
|  | 10 | 1.00 | 0.99 | 1.00 | 1.00 | 1.00 | 1.00 | 1.00 | 1.00 | 1.00 | 0-10 | 1.02 | 0.98 | 1.05 | 1.03 | 1.00 | 1.06 | 1.02 | 0.99 | 1.06 |
|  | 11 | 1.00 | 0.99 | 1.00 | 1.00 | 1.00 | 1.00 | 1.00 | 1.00 | 1.00 | 0-11 | 1.01 | 0.98 | 1.05 | 1.03 | 1.00 | 1.06 | 1.02 | 0.99 | 1.06 |
|  | 12 | 1.00 | 0.99 | 1.00 | 1.00 | 1.00 | 1.00 | 1.00 | 1.00 | 1.00 | 0-12 | 1.01 | 0.98 | 1.05 | 1.03 | 0.99 | 1.07 | 1.02 | 0.99 | 1.06 |
|  | 13 | 1.00 | 0.99 | 1.00 | 1.00 | 1.00 | 1.00 | 1.00 | 1.00 | 1.00 | 0-13 | 1.01 | 0.97 | 1.05 | 1.03 | 0.99 | 1.07 | 1.02 | 0.98 | 1.06 |
|  | 14 | 1.00 | 0.99 | 1.00 | 1.00 | 1.00 | 1.00 | 1.00 | 1.00 | 1.00 | 0-14 | 1.01 | 0.97 | 1.05 | 1.03 | 0.99 | 1.07 | 1.02 | 0.98 | 1.06 |
|  | 15 | 1.00 | 0.99 | 1.00 | 1.00 | 1.00 | 1.00 | 1.00 | 0.99 | 1.00 | 0-15 | 1.00 | 0.96 | 1.04 | 1.03 | 0.99 | 1.07 | 1.02 | 0.98 | 1.06 |
|  | 16 | 1.00 | 0.99 | 1.00 | 1.00 | 0.99 | 1.00 | 1.00 | 0.99 | 1.00 | 0-16 | 1.00 | 0.96 | 1.04 | 1.02 | 0.98 | 1.07 | 1.01 | 0.97 | 1.06 |
|  | 17 | 1.00 | 0.99 | 1.00 | 1.00 | 0.99 | 1.00 | 1.00 | 0.99 | 1.00 | 0-17 | 1.00 | 0.96 | 1.04 | 1.02 | 0.98 | 1.06 | 1.01 | 0.97 | 1.05 |
|  | 18 | 1.00 | 0.99 | 1.00 | 1.00 | 0.99 | 1.00 | 1.00 | 0.99 | 1.00 | 0-18 | 0.99 | 0.95 | 1.04 | 1.02 | 0.98 | 1.06 | 1.01 | 0.97 | 1.05 |
|  | 19 | 1.00 | 0.99 | 1.00 | 1.00 | 0.99 | 1.00 | 1.00 | 0.99 | 1.00 | 0-19 | 0.99 | 0.95 | 1.04 | 1.02 | 0.98 | 1.06 | 1.01 | 0.96 | 1.05 |
|  | 20 | 1.00 | 0.99 | 1.00 | 1.00 | 0.99 | 1.00 | 1.00 | 0.99 | 1.00 | 0-20 | 0.99 | 0.94 | 1.03 | 1.02 | 0.97 | 1.06 | 1.00 | 0.96 | 1.05 |
|  | 21 | 1.00 | 0.99 | 1.01 | 1.00 | 0.99 | 1.01 | 1.00 | 0.99 | 1.01 | 0-21 | 0.99 | 0.94 | 1.04 | 1.02 | 0.97 | 1.07 | 1.00 | 0.95 | 1.05 |

Note. Statistically significant (*p* < 0.05) were labeled in bold font; RR: relative risk; UCI: upper confidence interval; LCI: lower confidence interval; DHI: diffuse horizontal irradiance; DNI: direct normal irradiance; GHI: global horizontal irradiance; df: degrees of freedom.

**Supplementary Table 6.** The sensitivity analysis of changing df for meteorological factors (2–4) demonstrated the impacts of solar radiation on outpatient visits for meibomian gland dysfunction.

|  | Single-day lag RR (95%CI) | | | | | | | | | | Cumulative-day lag RR (95%CI) | | | | | | | | | |
| --- | --- | --- | --- | --- | --- | --- | --- | --- | --- | --- | --- | --- | --- | --- | --- | --- | --- | --- | --- | --- |
|  |  | Meteorological factors df = 2 | | | Meteorological factors df = 3 | | | Meteorological factors df = 4 | | |  | Meteorological factors df = 2 | | | Meteorological factors df = 3 | | | Meteorological factors df = 4 | | |
|  | Lag days | RR | LCI | UCI | RR | LCI | UCI | RR | LCI | UCI | Lag days | RR | LCI | UCI | RR | LCI | UCI | RR | LCI | UCI |
| GHI | 0 | 1.02 | 1.00 | 1.04 | 1.02 | 1.00 | 1.04 | 1.02 | 1.00 | 1.04 | 0-0 | 1.02 | 1.00 | 1.04 | 1.02 | 1.00 | 1.04 | 1.02 | 1.00 | 1.04 |
|  | 1 | 1.01 | 1.00 | 1.02 | 1.01 | 1.00 | 1.02 | 1.01 | 1.00 | 1.02 | 0-1 | 1.03 | 1.00 | 1.06 | 1.03 | 1.00 | 1.06 | 1.03 | 1.00 | 1.06 |
|  | 2 | 1.01 | 1.00 | 1.02 | 1.01 | 1.00 | 1.02 | 1.01 | 1.00 | 1.02 | 0-2 | 1.04 | 1.00 | 1.07 | 1.04 | 1.00 | 1.07 | 1.04 | 1.00 | 1.07 |
|  | 3 | 1.01 | 1.00 | 1.01 | 1.01 | 1.00 | 1.01 | 1.01 | 1.00 | 1.01 | 0-3 | 1.04 | 1.01 | 1.08 | 1.04 | 1.00 | 1.08 | 1.04 | 1.00 | 1.08 |
|  | 4 | 1.00 | 1.00 | 1.01 | 1.00 | 1.00 | 1.01 | 1.00 | 1.00 | 1.01 | 0-4 | 1.05 | 1.01 | 1.09 | 1.05 | 1.01 | 1.09 | 1.05 | 1.01 | 1.09 |
|  | 5 | 1.00 | 1.00 | 1.01 | 1.00 | 1.00 | 1.01 | 1.00 | 1.00 | 1.01 | 0-5 | 1.05 | 1.01 | 1.10 | 1.05 | 1.01 | 1.09 | 1.05 | 1.01 | 1.09 |
|  | 6 | 1.00 | 1.00 | 1.01 | 1.00 | 1.00 | 1.01 | 1.00 | 1.00 | 1.01 | 0-6 | 1.06 | 1.01 | 1.10 | 1.05 | 1.01 | 1.10 | 1.05 | 1.01 | 1.10 |
|  | 7 | 1.00 | 1.00 | 1.01 | 1.00 | 1.00 | 1.01 | 1.00 | 1.00 | 1.01 | 0-7 | 1.06 | 1.01 | 1.11 | 1.05 | 1.01 | 1.10 | 1.05 | 1.01 | 1.10 |
|  | 8 | 1.00 | 1.00 | 1.01 | 1.00 | 1.00 | 1.01 | 1.00 | 1.00 | 1.01 | 0-8 | 1.06 | 1.01 | 1.11 | 1.06 | 1.01 | 1.11 | 1.06 | 1.01 | 1.11 |
|  | 9 | 1.00 | 1.00 | 1.01 | 1.00 | 1.00 | 1.01 | 1.00 | 1.00 | 1.01 | 0-9 | 1.06 | 1.01 | 1.11 | 1.06 | 1.01 | 1.11 | 1.06 | 1.01 | 1.11 |
|  | 10 | 1.00 | 1.00 | 1.01 | 1.00 | 1.00 | 1.01 | 1.00 | 1.00 | 1.01 | 0-10 | 1.06 | 1.01 | 1.12 | 1.06 | 1.01 | 1.11 | 1.06 | 1.01 | 1.12 |
|  | 11 | 1.00 | 1.00 | 1.01 | 1.00 | 1.00 | 1.01 | 1.00 | 1.00 | 1.01 | 0-11 | 1.07 | 1.01 | 1.12 | 1.06 | 1.01 | 1.12 | 1.06 | 1.01 | 1.12 |
|  | 12 | 1.00 | 1.00 | 1.01 | 1.00 | 1.00 | 1.01 | 1.00 | 1.00 | 1.01 | 0-12 | 1.07 | 1.01 | 1.13 | 1.06 | 1.01 | 1.12 | 1.06 | 1.00 | 1.12 |
|  | 13 | 1.00 | 1.00 | 1.01 | 1.00 | 1.00 | 1.01 | 1.00 | 0.99 | 1.01 | 0-13 | 1.07 | 1.01 | 1.13 | 1.06 | 1.00 | 1.13 | 1.06 | 1.00 | 1.13 |
|  | 14 | 1.00 | 0.99 | 1.01 | 1.00 | 0.99 | 1.00 | 1.00 | 0.99 | 1.00 | 0-14 | 1.07 | 1.01 | 1.14 | 1.06 | 1.00 | 1.13 | 1.06 | 1.00 | 1.13 |
|  | 15 | 1.00 | 0.99 | 1.00 | 1.00 | 0.99 | 1.00 | 1.00 | 0.99 | 1.00 | 0-15 | 1.07 | 1.00 | 1.14 | 1.06 | 1.00 | 1.13 | 1.06 | 1.00 | 1.13 |
|  | 16 | 1.00 | 0.99 | 1.00 | 1.00 | 0.99 | 1.00 | 1.00 | 0.99 | 1.00 | 0-16 | 1.07 | 1.00 | 1.14 | 1.06 | 0.99 | 1.13 | 1.06 | 0.99 | 1.13 |
|  | 17 | 1.00 | 0.99 | 1.00 | 1.00 | 0.99 | 1.00 | 1.00 | 0.99 | 1.00 | 0-17 | 1.06 | 1.00 | 1.14 | 1.06 | 0.99 | 1.13 | 1.06 | 0.99 | 1.13 |
|  | 18 | 1.00 | 0.99 | 1.00 | 1.00 | 0.99 | 1.00 | 1.00 | 0.99 | 1.00 | 0-18 | 1.06 | 0.99 | 1.14 | 1.05 | 0.98 | 1.13 | 1.05 | 0.98 | 1.13 |
|  | 19 | 1.00 | 0.99 | 1.00 | 1.00 | 0.99 | 1.00 | 1.00 | 0.99 | 1.00 | 0-19 | 1.06 | 0.99 | 1.14 | 1.05 | 0.98 | 1.13 | 1.05 | 0.98 | 1.13 |
|  | 20 | 1.00 | 0.99 | 1.01 | 1.00 | 0.99 | 1.01 | 1.00 | 0.99 | 1.01 | 0-20 | 1.06 | 0.98 | 1.14 | 1.05 | 0.97 | 1.13 | 1.05 | 0.97 | 1.12 |
|  | 21 | 1.00 | 0.99 | 1.01 | 1.00 | 0.99 | 1.01 | 1.00 | 0.98 | 1.01 | 0-21 | 1.05 | 0.98 | 1.14 | 1.05 | 0.97 | 1.13 | 1.04 | 0.97 | 1.13 |
| DHI | 0 | 1.02 | 0.98 | 1.07 | 1.02 | 0.98 | 1.07 | 1.03 | 0.98 | 1.07 | 0-0 | 1.02 | 0.98 | 1.07 | 1.02 | 0.98 | 1.07 | 1.03 | 0.98 | 1.07 |
|  | 1 | 1.01 | 0.98 | 1.03 | 1.01 | 0.98 | 1.04 | 1.01 | 0.98 | 1.04 | 0-1 | 1.03 | 0.96 | 1.10 | 1.03 | 0.96 | 1.11 | 1.04 | 0.96 | 1.11 |
|  | 2 | 1.00 | 0.98 | 1.02 | 1.00 | 0.98 | 1.02 | 1.00 | 0.98 | 1.02 | 0-2 | 1.03 | 0.94 | 1.12 | 1.03 | 0.94 | 1.13 | 1.04 | 0.95 | 1.13 |
|  | 3 | 1.00 | 0.98 | 1.02 | 1.00 | 0.98 | 1.02 | 1.00 | 0.98 | 1.02 | 0-3 | 1.03 | 0.93 | 1.13 | 1.03 | 0.93 | 1.14 | 1.04 | 0.94 | 1.15 |
|  | 4 | 1.00 | 0.98 | 1.02 | 1.00 | 0.98 | 1.02 | 1.00 | 0.98 | 1.02 | 0-4 | 1.03 | 0.92 | 1.15 | 1.03 | 0.92 | 1.15 | 1.04 | 0.93 | 1.16 |
|  | 5 | 1.01 | 0.99 | 1.03 | 1.01 | 0.99 | 1.03 | 1.01 | 0.99 | 1.03 | 0-5 | 1.04 | 0.92 | 1.17 | 1.04 | 0.92 | 1.18 | 1.05 | 0.92 | 1.18 |
|  | 6 | 1.02 | 1.00 | 1.04 | 1.01 | 0.99 | 1.03 | 1.01 | 0.99 | 1.03 | 0-6 | 1.05 | 0.92 | 1.20 | 1.05 | 0.92 | 1.20 | 1.06 | 0.93 | 1.22 |
|  | 7 | 1.02 | 1.00 | 1.04 | 1.02 | 1.00 | 1.04 | 1.02 | 1.00 | 1.04 | 0-7 | 1.08 | 0.93 | 1.24 | 1.08 | 0.93 | 1.24 | 1.08 | 0.94 | 1.25 |
|  | 8 | 1.03 | 1.01 | 1.05 | 1.03 | 1.01 | 1.05 | 1.03 | 1.01 | 1.05 | 0-8 | 1.11 | 0.95 | 1.29 | 1.10 | 0.95 | 1.29 | 1.11 | 0.95 | 1.30 |
|  | 9 | 1.03 | 1.01 | 1.05 | 1.03 | 1.01 | 1.05 | 1.03 | 1.01 | 1.05 | 0-9 | 1.15 | 0.97 | 1.35 | 1.14 | 0.96 | 1.34 | 1.15 | 0.97 | 1.35 |
|  | 10 | 1.04 | 1.01 | 1.06 | 1.03 | 1.01 | 1.05 | 1.03 | 1.01 | 1.05 | 0-10 | 1.19 | 1.00 | 1.41 | 1.17 | 0.98 | 1.40 | 1.18 | 0.99 | 1.41 |
|  | 11 | 1.03 | 1.01 | 1.06 | 1.03 | 1.01 | 1.05 | 1.03 | 1.01 | 1.05 | 0-11 | 1.23 | 1.02 | 1.48 | 1.21 | 1.00 | 1.46 | 1.22 | 1.01 | 1.48 |
|  | 12 | 1.03 | 1.01 | 1.05 | 1.03 | 1.01 | 1.05 | 1.03 | 1.01 | 1.05 | 0-12 | 1.27 | 1.04 | 1.54 | 1.24 | 1.02 | 1.52 | 1.26 | 1.03 | 1.53 |
|  | 13 | 1.03 | 1.01 | 1.05 | 1.02 | 1.00 | 1.04 | 1.02 | 1.00 | 1.04 | 0-13 | 1.30 | 1.06 | 1.60 | 1.27 | 1.03 | 1.57 | 1.29 | 1.05 | 1.59 |
|  | 14 | 1.02 | 1.00 | 1.04 | 1.02 | 1.00 | 1.04 | 1.02 | 1.00 | 1.04 | 0-14 | 1.32 | 1.07 | 1.64 | 1.29 | 1.04 | 1.61 | 1.31 | 1.05 | 1.63 |
|  | 15 | 1.01 | 0.99 | 1.03 | 1.01 | 0.99 | 1.03 | 1.01 | 0.99 | 1.03 | 0-15 | 1.34 | 1.07 | 1.67 | 1.31 | 1.04 | 1.64 | 1.32 | 1.05 | 1.66 |
|  | 16 | 1.00 | 0.98 | 1.02 | 1.00 | 0.98 | 1.02 | 1.00 | 0.98 | 1.02 | 0-16 | 1.34 | 1.06 | 1.69 | 1.31 | 1.04 | 1.66 | 1.33 | 1.05 | 1.68 |
|  | 17 | 1.00 | 0.97 | 1.02 | 1.00 | 0.97 | 1.02 | 1.00 | 0.97 | 1.02 | 0-17 | 1.34 | 1.05 | 1.70 | 1.31 | 1.02 | 1.67 | 1.32 | 1.03 | 1.69 |
|  | 18 | 0.99 | 0.97 | 1.02 | 0.99 | 0.97 | 1.02 | 0.99 | 0.97 | 1.02 | 0-18 | 1.33 | 1.03 | 1.71 | 1.30 | 1.01 | 1.67 | 1.31 | 1.02 | 1.69 |
|  | 19 | 1.00 | 0.97 | 1.02 | 1.00 | 0.97 | 1.02 | 1.00 | 0.97 | 1.02 | 0-19 | 1.32 | 1.02 | 1.72 | 1.29 | 1.00 | 1.68 | 1.31 | 1.01 | 1.70 |
|  | 20 | 1.01 | 0.98 | 1.03 | 1.00 | 0.98 | 1.03 | 1.01 | 0.98 | 1.03 | 0-20 | 1.33 | 1.02 | 1.74 | 1.30 | 0.99 | 1.70 | 1.32 | 1.00 | 1.73 |
|  | 21 | 1.03 | 0.98 | 1.07 | 1.02 | 0.98 | 1.07 | 1.02 | 0.98 | 1.07 | 0-21 | 1.37 | 1.03 | 1.81 | 1.33 | 1.00 | 1.76 | 1.35 | 1.01 | 1.79 |
| DNI | 0 | 1.01 | 1.00 | 1.02 | 1.01 | 1.00 | 1.02 | 1.01 | 1.00 | 1.02 | 0-0 | 1.01 | 1.00 | 1.02 | 1.01 | 1.00 | 1.02 | 1.01 | 1.00 | 1.02 |
|  | 1 | 1.01 | 1.00 | 1.01 | 1.01 | 1.00 | 1.01 | 1.01 | 1.00 | 1.01 | 0-1 | 1.02 | 1.00 | 1.04 | 1.02 | 1.00 | 1.04 | 1.02 | 1.00 | 1.04 |
|  | 2 | 1.01 | 1.00 | 1.01 | 1.00 | 1.00 | 1.01 | 1.00 | 1.00 | 1.01 | 0-2 | 1.02 | 1.00 | 1.05 | 1.02 | 1.00 | 1.04 | 1.02 | 1.00 | 1.04 |
|  | 3 | 1.00 | 1.00 | 1.01 | 1.00 | 1.00 | 1.01 | 1.00 | 1.00 | 1.01 | 0-3 | 1.03 | 1.00 | 1.05 | 1.03 | 1.00 | 1.05 | 1.03 | 1.00 | 1.05 |
|  | 4 | 1.00 | 1.00 | 1.01 | 1.00 | 1.00 | 1.01 | 1.00 | 1.00 | 1.01 | 0-4 | 1.03 | 1.01 | 1.06 | 1.03 | 1.00 | 1.05 | 1.03 | 1.00 | 1.05 |
|  | 5 | 1.00 | 1.00 | 1.01 | 1.00 | 1.00 | 1.01 | 1.00 | 1.00 | 1.01 | 0-5 | 1.03 | 1.01 | 1.06 | 1.03 | 1.00 | 1.06 | 1.03 | 1.00 | 1.06 |
|  | 6 | 1.00 | 1.00 | 1.00 | 1.00 | 1.00 | 1.00 | 1.00 | 1.00 | 1.00 | 0-6 | 1.03 | 1.01 | 1.06 | 1.03 | 1.00 | 1.06 | 1.03 | 1.00 | 1.06 |
|  | 7 | 1.00 | 1.00 | 1.00 | 1.00 | 1.00 | 1.00 | 1.00 | 1.00 | 1.00 | 0-7 | 1.03 | 1.01 | 1.06 | 1.03 | 1.00 | 1.06 | 1.03 | 1.00 | 1.06 |
|  | 8 | 1.00 | 1.00 | 1.00 | 1.00 | 1.00 | 1.00 | 1.00 | 1.00 | 1.00 | 0-8 | 1.03 | 1.00 | 1.06 | 1.03 | 1.00 | 1.06 | 1.03 | 1.00 | 1.06 |
|  | 9 | 1.00 | 1.00 | 1.00 | 1.00 | 1.00 | 1.00 | 1.00 | 1.00 | 1.00 | 0-9 | 1.03 | 1.00 | 1.07 | 1.03 | 1.00 | 1.06 | 1.03 | 1.00 | 1.06 |
|  | 10 | 1.00 | 1.00 | 1.00 | 1.00 | 1.00 | 1.00 | 1.00 | 1.00 | 1.00 | 0-10 | 1.03 | 1.00 | 1.07 | 1.03 | 1.00 | 1.06 | 1.03 | 1.00 | 1.06 |
|  | 11 | 1.00 | 1.00 | 1.00 | 1.00 | 1.00 | 1.00 | 1.00 | 1.00 | 1.00 | 0-11 | 1.03 | 1.00 | 1.07 | 1.03 | 1.00 | 1.06 | 1.03 | 1.00 | 1.06 |
|  | 12 | 1.00 | 1.00 | 1.00 | 1.00 | 1.00 | 1.00 | 1.00 | 1.00 | 1.00 | 0-12 | 1.03 | 1.00 | 1.07 | 1.03 | 0.99 | 1.07 | 1.03 | 0.99 | 1.06 |
|  | 13 | 1.00 | 1.00 | 1.00 | 1.00 | 1.00 | 1.00 | 1.00 | 1.00 | 1.00 | 0-13 | 1.03 | 1.00 | 1.07 | 1.03 | 0.99 | 1.07 | 1.03 | 0.99 | 1.07 |
|  | 14 | 1.00 | 1.00 | 1.00 | 1.00 | 1.00 | 1.00 | 1.00 | 1.00 | 1.00 | 0-14 | 1.03 | 0.99 | 1.07 | 1.03 | 0.99 | 1.07 | 1.03 | 0.99 | 1.06 |
|  | 15 | 1.00 | 1.00 | 1.00 | 1.00 | 1.00 | 1.00 | 1.00 | 1.00 | 1.00 | 0-15 | 1.03 | 0.99 | 1.07 | 1.03 | 0.99 | 1.07 | 1.02 | 0.99 | 1.06 |
|  | 16 | 1.00 | 0.99 | 1.00 | 1.00 | 0.99 | 1.00 | 1.00 | 0.99 | 1.00 | 0-16 | 1.03 | 0.99 | 1.07 | 1.02 | 0.98 | 1.07 | 1.02 | 0.98 | 1.06 |
|  | 17 | 1.00 | 0.99 | 1.00 | 1.00 | 0.99 | 1.00 | 1.00 | 0.99 | 1.00 | 0-17 | 1.03 | 0.99 | 1.07 | 1.02 | 0.98 | 1.06 | 1.02 | 0.98 | 1.06 |
|  | 18 | 1.00 | 0.99 | 1.00 | 1.00 | 0.99 | 1.00 | 1.00 | 0.99 | 1.00 | 0-18 | 1.02 | 0.98 | 1.07 | 1.02 | 0.98 | 1.06 | 1.02 | 0.98 | 1.06 |
|  | 19 | 1.00 | 0.99 | 1.00 | 1.00 | 0.99 | 1.00 | 1.00 | 0.99 | 1.00 | 0-19 | 1.02 | 0.98 | 1.07 | 1.02 | 0.98 | 1.06 | 1.02 | 0.97 | 1.06 |
|  | 20 | 1.00 | 0.99 | 1.00 | 1.00 | 0.99 | 1.00 | 1.00 | 0.99 | 1.00 | 0-20 | 1.02 | 0.98 | 1.07 | 1.02 | 0.97 | 1.06 | 1.02 | 0.97 | 1.06 |
|  | 21 | 1.00 | 0.99 | 1.01 | 1.00 | 0.99 | 1.01 | 1.00 | 0.99 | 1.01 | 0-21 | 1.02 | 0.97 | 1.07 | 1.02 | 0.97 | 1.07 | 1.01 | 0.97 | 1.06 |

Note. Statistically significant (*p* < 0.05) were labeled in bold font; RR: relative risk; UCI: upper confidence interval; LCI: lower confidence interval; DHI: diffuse horizontal irradiance; DNI: direct normal irradiance; GHI: global horizontal irradiance; df: degrees of freedom.

**Supplementary Table 7.** The sensitivity analysis of changing df for air pollutants (2–4) demonstrated the impacts of solar radiation on outpatient visits for meibomian gland dysfunction.

|  | Single-day lag RR (95%CI) | | | | | | | | | | Cumulative-day lag RR (95%CI) | | | | | | | | | |
| --- | --- | --- | --- | --- | --- | --- | --- | --- | --- | --- | --- | --- | --- | --- | --- | --- | --- | --- | --- | --- |
|  |  | Air pollutants df = 2 | | | Air pollutants df = 3 | | | Air pollutants df = 4 | | |  | Air pollutants df = 2 | | | Air pollutants df = 3 | | | Air pollutants df = 4 | | |
|  | Lag days | RR | LCI | UCI | RR | LCI | UCI | RR | LCI | UCI | Lag days | RR | LCI | UCI | RR | LCI | UCI | RR | LCI | UCI |
| GHI | 0 | 1.02 | 1.00 | 1.04 | 1.02 | 1.00 | 1.04 | 1.02 | 1.00 | 1.04 | 0-0 | 1.02 | 1.00 | 1.04 | 1.02 | 1.00 | 1.04 | 1.02 | 1.00 | 1.04 |
|  | 1 | 1.01 | 1.00 | 1.02 | 1.01 | 1.00 | 1.02 | 1.01 | 1.00 | 1.02 | 0-1 | 1.03 | 1.00 | 1.06 | 1.03 | 1.00 | 1.06 | 1.03 | 1.00 | 1.06 |
|  | 2 | 1.01 | 1.00 | 1.02 | 1.01 | 1.00 | 1.02 | 1.01 | 1.00 | 1.02 | 0-2 | 1.04 | 1.00 | 1.07 | 1.04 | 1.00 | 1.07 | 1.04 | 1.00 | 1.07 |
|  | 3 | 1.01 | 1.00 | 1.01 | 1.01 | 1.00 | 1.01 | 1.01 | 1.00 | 1.01 | 0-3 | 1.04 | 1.01 | 1.08 | 1.04 | 1.00 | 1.08 | 1.04 | 1.00 | 1.08 |
|  | 4 | 1.00 | 1.00 | 1.01 | 1.00 | 1.00 | 1.01 | 1.00 | 1.00 | 1.01 | 0-4 | 1.05 | 1.01 | 1.09 | 1.05 | 1.01 | 1.09 | 1.05 | 1.01 | 1.09 |
|  | 5 | 1.00 | 1.00 | 1.01 | 1.00 | 1.00 | 1.01 | 1.00 | 1.00 | 1.01 | 0-5 | 1.05 | 1.01 | 1.09 | 1.05 | 1.01 | 1.09 | 1.05 | 1.01 | 1.09 |
|  | 6 | 1.00 | 1.00 | 1.01 | 1.00 | 1.00 | 1.01 | 1.00 | 1.00 | 1.01 | 0-6 | 1.05 | 1.01 | 1.10 | 1.05 | 1.01 | 1.10 | 1.05 | 1.01 | 1.10 |
|  | 7 | 1.00 | 1.00 | 1.01 | 1.00 | 1.00 | 1.01 | 1.00 | 1.00 | 1.01 | 0-7 | 1.05 | 1.01 | 1.10 | 1.05 | 1.01 | 1.10 | 1.06 | 1.01 | 1.10 |
|  | 8 | 1.00 | 1.00 | 1.01 | 1.00 | 1.00 | 1.01 | 1.00 | 1.00 | 1.01 | 0-8 | 1.06 | 1.01 | 1.11 | 1.06 | 1.01 | 1.11 | 1.06 | 1.01 | 1.11 |
|  | 9 | 1.00 | 1.00 | 1.01 | 1.00 | 1.00 | 1.01 | 1.00 | 1.00 | 1.01 | 0-9 | 1.06 | 1.01 | 1.11 | 1.06 | 1.01 | 1.11 | 1.06 | 1.01 | 1.11 |
|  | 10 | 1.00 | 1.00 | 1.01 | 1.00 | 1.00 | 1.01 | 1.00 | 1.00 | 1.01 | 0-10 | 1.06 | 1.01 | 1.12 | 1.06 | 1.01 | 1.11 | 1.06 | 1.01 | 1.12 |
|  | 11 | 1.00 | 1.00 | 1.01 | 1.00 | 1.00 | 1.01 | 1.00 | 1.00 | 1.01 | 0-11 | 1.06 | 1.01 | 1.12 | 1.06 | 1.01 | 1.12 | 1.06 | 1.01 | 1.12 |
|  | 12 | 1.00 | 1.00 | 1.01 | 1.00 | 1.00 | 1.01 | 1.00 | 1.00 | 1.01 | 0-12 | 1.06 | 1.01 | 1.12 | 1.06 | 1.01 | 1.12 | 1.07 | 1.01 | 1.13 |
|  | 13 | 1.00 | 1.00 | 1.01 | 1.00 | 1.00 | 1.01 | 1.00 | 0.99 | 1.01 | 0-13 | 1.06 | 1.00 | 1.13 | 1.06 | 1.00 | 1.13 | 1.07 | 1.01 | 1.13 |
|  | 14 | 1.00 | 0.99 | 1.00 | 1.00 | 0.99 | 1.00 | 1.00 | 0.99 | 1.00 | 0-14 | 1.06 | 1.00 | 1.13 | 1.06 | 1.00 | 1.13 | 1.07 | 1.00 | 1.13 |
|  | 15 | 1.00 | 0.99 | 1.00 | 1.00 | 0.99 | 1.00 | 1.00 | 0.99 | 1.00 | 0-15 | 1.06 | 1.00 | 1.13 | 1.06 | 1.00 | 1.13 | 1.06 | 1.00 | 1.13 |
|  | 16 | 1.00 | 0.99 | 1.00 | 1.00 | 0.99 | 1.00 | 1.00 | 0.99 | 1.00 | 0-16 | 1.06 | 0.99 | 1.13 | 1.06 | 0.99 | 1.13 | 1.06 | 0.99 | 1.13 |
|  | 17 | 1.00 | 0.99 | 1.00 | 1.00 | 0.99 | 1.00 | 1.00 | 0.99 | 1.00 | 0-17 | 1.06 | 0.99 | 1.13 | 1.06 | 0.99 | 1.13 | 1.06 | 0.99 | 1.13 |
|  | 18 | 1.00 | 0.99 | 1.00 | 1.00 | 0.99 | 1.00 | 1.00 | 0.99 | 1.00 | 0-18 | 1.05 | 0.99 | 1.13 | 1.05 | 0.98 | 1.13 | 1.05 | 0.98 | 1.13 |
|  | 19 | 1.00 | 0.99 | 1.00 | 1.00 | 0.99 | 1.00 | 1.00 | 0.99 | 1.00 | 0-19 | 1.05 | 0.98 | 1.13 | 1.05 | 0.98 | 1.13 | 1.05 | 0.98 | 1.13 |
|  | 20 | 1.00 | 0.99 | 1.01 | 1.00 | 0.99 | 1.01 | 1.00 | 0.99 | 1.00 | 0-20 | 1.05 | 0.98 | 1.13 | 1.05 | 0.97 | 1.13 | 1.04 | 0.97 | 1.12 |
|  | 21 | 1.00 | 0.99 | 1.01 | 1.00 | 0.99 | 1.01 | 1.00 | 0.98 | 1.01 | 0-21 | 1.05 | 0.97 | 1.13 | 1.05 | 0.97 | 1.13 | 1.04 | 0.97 | 1.13 |
| DHI | 0 | 1.02 | 0.98 | 1.07 | 1.02 | 0.98 | 1.07 | 1.02 | 0.98 | 1.07 | 0-0 | 1.02 | 0.98 | 1.07 | 1.02 | 0.98 | 1.07 | 1.02 | 0.98 | 1.07 |
|  | 1 | 1.01 | 0.98 | 1.04 | 1.01 | 0.98 | 1.04 | 1.01 | 0.98 | 1.04 | 0-1 | 1.03 | 0.96 | 1.10 | 1.03 | 0.96 | 1.11 | 1.03 | 0.96 | 1.11 |
|  | 2 | 1.00 | 0.98 | 1.02 | 1.00 | 0.98 | 1.02 | 1.00 | 0.98 | 1.02 | 0-2 | 1.03 | 0.94 | 1.12 | 1.03 | 0.94 | 1.13 | 1.03 | 0.95 | 1.13 |
|  | 3 | 1.00 | 0.98 | 1.02 | 1.00 | 0.98 | 1.02 | 1.00 | 0.98 | 1.02 | 0-3 | 1.03 | 0.93 | 1.14 | 1.03 | 0.93 | 1.14 | 1.03 | 0.93 | 1.14 |
|  | 4 | 1.00 | 0.98 | 1.03 | 1.00 | 0.98 | 1.02 | 1.00 | 0.98 | 1.03 | 0-4 | 1.03 | 0.92 | 1.15 | 1.03 | 0.92 | 1.15 | 1.04 | 0.93 | 1.16 |
|  | 5 | 1.01 | 0.99 | 1.03 | 1.01 | 0.99 | 1.03 | 1.01 | 0.99 | 1.03 | 0-5 | 1.04 | 0.92 | 1.18 | 1.04 | 0.92 | 1.18 | 1.04 | 0.92 | 1.18 |
|  | 6 | 1.01 | 0.99 | 1.03 | 1.01 | 0.99 | 1.03 | 1.01 | 0.99 | 1.04 | 0-6 | 1.06 | 0.92 | 1.21 | 1.05 | 0.92 | 1.20 | 1.06 | 0.93 | 1.21 |
|  | 7 | 1.02 | 1.00 | 1.04 | 1.02 | 1.00 | 1.04 | 1.02 | 1.00 | 1.04 | 0-7 | 1.08 | 0.93 | 1.24 | 1.08 | 0.93 | 1.24 | 1.08 | 0.94 | 1.25 |
|  | 8 | 1.03 | 1.01 | 1.05 | 1.03 | 1.01 | 1.05 | 1.03 | 1.01 | 1.05 | 0-8 | 1.10 | 0.95 | 1.29 | 1.10 | 0.95 | 1.29 | 1.11 | 0.95 | 1.30 |
|  | 9 | 1.03 | 1.01 | 1.05 | 1.03 | 1.01 | 1.05 | 1.03 | 1.01 | 1.05 | 0-9 | 1.14 | 0.96 | 1.34 | 1.14 | 0.96 | 1.34 | 1.15 | 0.97 | 1.35 |
|  | 10 | 1.03 | 1.01 | 1.05 | 1.03 | 1.01 | 1.05 | 1.03 | 1.01 | 1.05 | 0-10 | 1.17 | 0.98 | 1.40 | 1.17 | 0.98 | 1.40 | 1.18 | 0.99 | 1.41 |
|  | 11 | 1.03 | 1.01 | 1.05 | 1.03 | 1.01 | 1.05 | 1.03 | 1.01 | 1.05 | 0-11 | 1.21 | 1.00 | 1.45 | 1.21 | 1.00 | 1.46 | 1.22 | 1.01 | 1.47 |
|  | 12 | 1.03 | 1.01 | 1.05 | 1.03 | 1.01 | 1.05 | 1.03 | 1.01 | 1.05 | 0-12 | 1.24 | 1.02 | 1.51 | 1.24 | 1.02 | 1.52 | 1.26 | 1.03 | 1.53 |
|  | 13 | 1.02 | 1.00 | 1.04 | 1.02 | 1.00 | 1.04 | 1.02 | 1.00 | 1.04 | 0-13 | 1.27 | 1.03 | 1.56 | 1.27 | 1.03 | 1.57 | 1.29 | 1.05 | 1.58 |
|  | 14 | 1.02 | 1.00 | 1.04 | 1.02 | 1.00 | 1.04 | 1.02 | 1.00 | 1.04 | 0-14 | 1.29 | 1.04 | 1.60 | 1.29 | 1.04 | 1.61 | 1.31 | 1.05 | 1.63 |
|  | 15 | 1.01 | 0.99 | 1.03 | 1.01 | 0.99 | 1.03 | 1.01 | 0.99 | 1.03 | 0-15 | 1.30 | 1.04 | 1.63 | 1.31 | 1.04 | 1.64 | 1.32 | 1.05 | 1.66 |
|  | 16 | 1.00 | 0.98 | 1.03 | 1.00 | 0.98 | 1.02 | 1.00 | 0.98 | 1.02 | 0-16 | 1.31 | 1.04 | 1.65 | 1.31 | 1.04 | 1.66 | 1.32 | 1.05 | 1.67 |
|  | 17 | 1.00 | 0.98 | 1.02 | 1.00 | 0.97 | 1.02 | 1.00 | 0.97 | 1.02 | 0-17 | 1.31 | 1.03 | 1.67 | 1.31 | 1.02 | 1.67 | 1.32 | 1.03 | 1.68 |
|  | 18 | 1.00 | 0.98 | 1.02 | 0.99 | 0.97 | 1.02 | 0.99 | 0.97 | 1.02 | 0-18 | 1.31 | 1.02 | 1.68 | 1.30 | 1.01 | 1.67 | 1.31 | 1.02 | 1.69 |
|  | 19 | 1.00 | 0.98 | 1.02 | 1.00 | 0.97 | 1.02 | 1.00 | 0.97 | 1.02 | 0-19 | 1.31 | 1.01 | 1.69 | 1.29 | 1.00 | 1.68 | 1.30 | 1.00 | 1.69 |
|  | 20 | 1.01 | 0.98 | 1.03 | 1.00 | 0.98 | 1.03 | 1.00 | 0.98 | 1.03 | 0-20 | 1.32 | 1.01 | 1.72 | 1.30 | 0.99 | 1.70 | 1.31 | 1.00 | 1.72 |
|  | 21 | 1.02 | 0.98 | 1.07 | 1.02 | 0.98 | 1.07 | 1.02 | 0.98 | 1.07 | 0-21 | 1.35 | 1.02 | 1.79 | 1.33 | 1.00 | 1.76 | 1.34 | 1.01 | 1.78 |
| DNI | 0 | 1.01 | 1.00 | 1.02 | 1.01 | 1.00 | 1.02 | 1.01 | 1.00 | 1.02 | 0-0 | 1.01 | 1.00 | 1.02 | 1.01 | 1.00 | 1.02 | 1.01 | 1.00 | 1.02 |
|  | 1 | 1.01 | 1.00 | 1.01 | 1.01 | 1.00 | 1.01 | 1.01 | 1.00 | 1.01 | 0-1 | 1.02 | 1.00 | 1.04 | 1.02 | 1.00 | 1.04 | 1.02 | 1.00 | 1.04 |
|  | 2 | 1.01 | 1.00 | 1.01 | 1.00 | 1.00 | 1.01 | 1.01 | 1.00 | 1.01 | 0-2 | 1.02 | 1.00 | 1.04 | 1.02 | 1.00 | 1.04 | 1.02 | 1.00 | 1.04 |
|  | 3 | 1.00 | 1.00 | 1.01 | 1.00 | 1.00 | 1.01 | 1.00 | 1.00 | 1.01 | 0-3 | 1.03 | 1.00 | 1.05 | 1.03 | 1.00 | 1.05 | 1.03 | 1.00 | 1.05 |
|  | 4 | 1.00 | 1.00 | 1.01 | 1.00 | 1.00 | 1.01 | 1.00 | 1.00 | 1.01 | 0-4 | 1.03 | 1.00 | 1.05 | 1.03 | 1.00 | 1.05 | 1.03 | 1.00 | 1.05 |
|  | 5 | 1.00 | 1.00 | 1.01 | 1.00 | 1.00 | 1.01 | 1.00 | 1.00 | 1.01 | 0-5 | 1.03 | 1.00 | 1.06 | 1.03 | 1.00 | 1.06 | 1.03 | 1.00 | 1.06 |
|  | 6 | 1.00 | 1.00 | 1.00 | 1.00 | 1.00 | 1.00 | 1.00 | 1.00 | 1.00 | 0-6 | 1.03 | 1.00 | 1.06 | 1.03 | 1.00 | 1.06 | 1.03 | 1.00 | 1.06 |
|  | 7 | 1.00 | 1.00 | 1.00 | 1.00 | 1.00 | 1.00 | 1.00 | 1.00 | 1.00 | 0-7 | 1.03 | 1.00 | 1.06 | 1.03 | 1.00 | 1.06 | 1.03 | 1.00 | 1.06 |
|  | 8 | 1.00 | 1.00 | 1.00 | 1.00 | 1.00 | 1.00 | 1.00 | 1.00 | 1.00 | 0-8 | 1.03 | 1.00 | 1.06 | 1.03 | 1.00 | 1.06 | 1.03 | 1.00 | 1.06 |
|  | 9 | 1.00 | 1.00 | 1.00 | 1.00 | 1.00 | 1.00 | 1.00 | 1.00 | 1.00 | 0-9 | 1.03 | 1.00 | 1.06 | 1.03 | 1.00 | 1.06 | 1.03 | 1.00 | 1.06 |
|  | 10 | 1.00 | 1.00 | 1.00 | 1.00 | 1.00 | 1.00 | 1.00 | 1.00 | 1.00 | 0-10 | 1.03 | 1.00 | 1.06 | 1.03 | 1.00 | 1.06 | 1.03 | 1.00 | 1.07 |
|  | 11 | 1.00 | 1.00 | 1.00 | 1.00 | 1.00 | 1.00 | 1.00 | 1.00 | 1.00 | 0-11 | 1.03 | 1.00 | 1.07 | 1.03 | 1.00 | 1.06 | 1.03 | 1.00 | 1.07 |
|  | 12 | 1.00 | 1.00 | 1.00 | 1.00 | 1.00 | 1.00 | 1.00 | 1.00 | 1.00 | 0-12 | 1.03 | 1.00 | 1.07 | 1.03 | 0.99 | 1.07 | 1.03 | 1.00 | 1.07 |
|  | 13 | 1.00 | 1.00 | 1.00 | 1.00 | 1.00 | 1.00 | 1.00 | 1.00 | 1.00 | 0-13 | 1.03 | 0.99 | 1.07 | 1.03 | 0.99 | 1.07 | 1.03 | 0.99 | 1.07 |
|  | 14 | 1.00 | 1.00 | 1.00 | 1.00 | 1.00 | 1.00 | 1.00 | 1.00 | 1.00 | 0-14 | 1.03 | 0.99 | 1.07 | 1.03 | 0.99 | 1.07 | 1.03 | 0.99 | 1.07 |
|  | 15 | 1.00 | 1.00 | 1.00 | 1.00 | 1.00 | 1.00 | 1.00 | 0.99 | 1.00 | 0-15 | 1.03 | 0.99 | 1.07 | 1.03 | 0.99 | 1.07 | 1.03 | 0.99 | 1.07 |
|  | 16 | 1.00 | 0.99 | 1.00 | 1.00 | 0.99 | 1.00 | 1.00 | 0.99 | 1.00 | 0-16 | 1.02 | 0.99 | 1.07 | 1.02 | 0.98 | 1.07 | 1.02 | 0.99 | 1.07 |
|  | 17 | 1.00 | 0.99 | 1.00 | 1.00 | 0.99 | 1.00 | 1.00 | 0.99 | 1.00 | 0-17 | 1.02 | 0.98 | 1.06 | 1.02 | 0.98 | 1.06 | 1.02 | 0.98 | 1.06 |
|  | 18 | 1.00 | 0.99 | 1.00 | 1.00 | 0.99 | 1.00 | 1.00 | 0.99 | 1.00 | 0-18 | 1.02 | 0.98 | 1.06 | 1.02 | 0.98 | 1.06 | 1.02 | 0.98 | 1.06 |
|  | 19 | 1.00 | 0.99 | 1.00 | 1.00 | 0.99 | 1.00 | 1.00 | 0.99 | 1.00 | 0-19 | 1.02 | 0.98 | 1.06 | 1.02 | 0.98 | 1.06 | 1.02 | 0.97 | 1.06 |
|  | 20 | 1.00 | 0.99 | 1.00 | 1.00 | 0.99 | 1.00 | 1.00 | 0.99 | 1.00 | 0-20 | 1.02 | 0.97 | 1.06 | 1.02 | 0.97 | 1.06 | 1.02 | 0.97 | 1.06 |
|  | 21 | 1.00 | 0.99 | 1.01 | 1.00 | 0.99 | 1.01 | 1.00 | 0.99 | 1.01 | 0-21 | 1.02 | 0.97 | 1.07 | 1.02 | 0.97 | 1.07 | 1.02 | 0.97 | 1.06 |

Note. Statistically significant (*p* < 0.05) were labeled in bold font; RR: relative risk; UCI: upper confidence interval; LCI: lower confidence interval; DHI: diffuse horizontal irradiance; DNI: direct normal irradiance; GHI: global horizontal irradiance; df: degrees of freedom.
